# A Review of Vagus Nerve Stimulation for Disease: Comprehensive Theory and Evidence for Mechanisms of Action

**DOI:** 10.1002/cph4.70109

**Published:** 2026-03-04

**Authors:** Yifeng Bu, Alex Liang, Benjamin U. Hoffman, Dawn M. Schiehser, Oliver Case, Alan Simmons, Ruth Klaming, Andres Gottfried‐Blackmore, Ravinder K. Mittal, Christopher Puleo, Hubert Lim, Imanuel Lerman

**Affiliations:** ^1^ InflammaSense Incorporated La Jolla California USA; ^2^ Qualcomm Institute University of California San Diego La Jolla California USA; ^3^ Department of Anesthesiology, Center for Pain Medicine University of California San Diego School of Medicine La Jolla California USA; ^4^ Division of Cardiovascular Medicine University of California San Diego School of Medicine La Jolla California USA; ^5^ Department of Psychiatry University of California, San Diego La Jolla California USA; ^6^ VA Center of Excellence for Stress and Mental Health VA San Diego Healthcare System La Jolla California USA; ^7^ VA San Diego Healthcare System La Jolla California USA; ^8^ Department of Clinical and Health Psychology University of Florida Florida USA; ^9^ British Heart Foundation Centre of Research Excellence, Institute of Cardiovascular Science Institute of Cognitive Neuroscience Queen Square Institute of Neurology University College London London UK; ^10^ Department of Pharmacology University of California San Diego School of Medicine La Jolla California USA; ^11^ Division of Gastroenterology, Department of Medicine University of California San Diego School of Medicine La Jolla California USA; ^12^ Department of Biomedical Engineering, Center for Biotechnology and Interdisciplinary Studies Rensselaer Polytechnic Institute Troy New York USA; ^13^ Departments of Biomedical Engineering and Otolaryngology‐Head & Neck Surgery University of Minnesota Minneapolis Minnesota USA; ^14^ SecondWave System Incorporated Saint Paul Minnesota USA

**Keywords:** bioelectronic medicine, brain modulation, inflammatory reflex, neuromodulation, vagus nerve stimulation

## Abstract

Vagus nerve stimulation (VNS) is an established neuromodulatory therapy approved for epilepsy, depression, obesity, stroke rehabilitation, rheumatoid arthritis, migraine, and cluster headaches. Its therapeutic potential has expanded dramatically, with growing evidence supporting its efficacy across a wide spectrum of neurological, psychiatric, cardiovascular, immunological, metabolic, and gastrointestinal disorders. Despite this progress, the field has lacked a comprehensive synthesis that unifies mechanistic insights with translational applications across organ systems. This review addresses that gap by systematically integrating current knowledge in the multifactorial mechanisms through which VNS modulates central and peripheral functions, including neuromodulator release, synaptic plasticity, autonomic regulation, neuroimmune control, and endocrine integration. In addition, this review identifies key limitations of VNS, including biological heterogeneity, technical constraints, and methodological variability, and proposes future innovations such as selective fiber targeting, closed‐loop systems, and artificial intelligence‐guided personalization. By providing a rigorous, system‐level overview of VNS mechanisms and their translational relevance, this article serves as a foundational resource for advancing the science and clinical deployment and helping illustrate future directions for precision neuromodulation and bioelectronic medicine.

## Graphical abstract

Vagus nerve stimulation (VNS) effects span central and peripheral organ systems through diverse mechanistic pathways. This comprehensive review provides a unified synthesis of these mechanisms across neurological, cardiovascular, immunological, metabolic, and gastrointestinal domains, filling a critical gap and serving as a foundational resource for bioelectronic medicine.

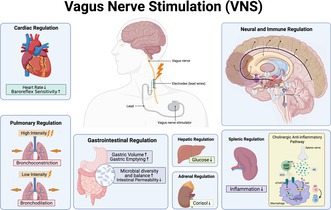

**Abbreviations**
5‐HT: 5‐hydroxytryptamine, serotonin
α7nAChR: α7 nicotinic acetylcholine receptor
ABVN: auricular branch of the vagus nerve
ACC: anterior cingulate cortex
ACh: acetylcholine
ACTH: adrenocorticotropic hormone
AD: Alzheimer's disease
ADAS‐cog: Alzheimer's Disease Assessment Scale‐Cognitive Subscale (ADAS‐Cog)
ADHD: attention‐deficit/hyperactivity disorder
AF: atrial fibrillation
AI: artificial intelligence
AKI: acute kidney injury
AMPK: AMP‐activated protein kinase
ARDS: acute respiratory distress syndrome
ASD: autism spectrum disorder
AV: atrioventricular
BAT: Brown adipose tissue
BBB: blood–brain barrier
BDNF: brain‐derived neurotrophic factor
CAP: cholinergic anti‐inflammatory pathway
CCK: cholecystokinin
CKD: chronic kidney disease
CNS: central nervous system
CRH: corticotropin‐releasing hormone
CRP: C‐reactive protein
CRS‐R: Revised Coma Recovery Scale
DA: dopamine
DAS: descending antinociceptive systems
DH: dorsal horn
DMV: dorsal motor nucleus of the vagus
DNIC: diffuse noxious inhibitory control
DoC: disorders of consciousness
DRN: dorsal raphe nucleus
EEG: electroencephalogram
EGC: enteric glial cells
FDA: Food and Drug Administration
fMRI: functional magnetic resonance imaging
FUS: focused ultrasound stimulation
GABA: γ‐aminobutyric acid
GAD: generalized anxiety disorder
GI: gastrointestinal
HFpEF: heart failure with preserved ejection fraction
HFrEF: heart failure with reduced ejection fraction
HPA: hypothalamic–pituitary–adrenal
HR: heart rate
HRV: heart rate variability
I/R: ischemia–reperfusion
IBD: inflammatory bowel disease
IBS: irritable bowel syndrome
IL‐10: interleukin‐10
IL‐1β: interleukin‐1 beta
IL‐4: interleukin‐4
IL‐6: interleukin‐6
IL‐8: interleukin‐8
iVNS: invasive vagus nerve stimulation
LC: locus coeruleus
LTD: long‐term depression
LTP: long‐term potentiation
LVEF: left ventricular ejection fraction
MADRS: Montgomery‐Åsberg Depression Rating Scale
MI: myocardial infarction
MMSE: Mini‐Mental State Examination
mPFC: medial PFC
mVNS: magnetic vagus nerve stimulation
NE: norepinephrine
NO: nitric oxide
NOS: nitric oxide synthase
NTS: nucleus tractus solitarius
NYHA: New York Heart Association
OCD: obsessive‐compulsive disorder
oVNS: optogenetic vagus nerve stimulation
PAG: periaqueductal gray
paVNS: percutaneous auricular vagus nerve stimulation
PBN: parabrachial nucleus
PD: Parkinson's disease
PET: positron emission tomography
PFC: prefrontal cortex
pSS: primary Sjögren's syndrome
PTSD: posttraumatic stress disorder
PVN: paraventricular nucleus
RA: rheumatoid arthritis
RAAS: renin‐angiotensin‐aldosterone system
RCT: randomized controlled trial
ROS: reactive oxygen species
RSA: respiratory sinus arrhythmia
RVM: rostral ventromedial medulla
SA: sinoatrial
SLE: systemic lupus erythematosus
SSc: systemic sclerosis
taVNS: transcutaneous auricular vagus nerve stimulation
TBI: traumatic brain injury
tcVNS: transcutaneous cervical vagus nerve stimulation
TNF‐α: tumor necrosis factor alpha
vBloc: vagal blocking
VIP: vasoactive intestinal polypeptide
vmPFC: ventromedial prefrontal cortex
VN: vagus nerve
VNS: vagus nerve stimulation
VTA: ventral tegmental area

## 1 Introduction

Vagus nerve stimulation (VNS) is a neuromodulation therapy that involves delivering mild electrical pulses to the vagus nerve (VN), a key communication highway between the brain and many vital organs. The concept of stimulating the vagus to treat disease is over a century old: as early as the late 19th century, physicians experimented with vagal stimulation for epilepsy (Badran and Austelle 2022). Modern VNS, however, took shape in the 1980s when researchers like Jacob Zabara reintroduced it as a potential treatment for refractory epilepsy. In 1988, the first human patient received an implanted VNS device, demonstrating a reduction in seizure frequency. This milestone was followed by U.S. Food and Drug Administration (FDA) approval of VNS in 1997 as an adjunct therapy for drug‐resistant epilepsy, with subsequent approvals for treatment‐resistant depression in 2005, for obesity in 2015, for stroke rehabilitation in 2021, and most recently for rheumatoid arthritis (RA) in 2025 (Badran and Austelle 2022). These historical milestones established VNS as a viable clinical intervention and set the stage for its expansion into other areas.

Over the past two decades, VNS has gained importance as an innovative treatment across numerous fields of medicine and neuroscience. Initially developed for neurological disorders, it is now recognized as a unique and potent intervention in both neurology and psychiatry (Austelle et al. 2024). Clinicians and researchers have extended the use of VNS beyond epilepsy to disorders such as major depression, and ongoing studies are exploring its benefits in conditions ranging from stroke rehabilitation to chronic inflammatory diseases and cardiovascular diseases (Austelle et al. 2024). The VN's extensive reach, interfacing with the brainstem and influencing the heart, lungs, spleen, stomach, intestine, and kidney, indicates that proximal cervical VNS stimulation modulates a wide array of body systems. As a result, VNS has become increasingly relevant not only for controlling seizures or improving mood, but also for regulating immune responses and visceral function (Redgrave, Day, et al. 2018). This broad therapeutic footprint underlines the growing enthusiasm for VNS in medical research, bridging disciplines such as neurology, psychiatry, immunology, and cardiology in the search for new treatments.

Given the expanding scope of VNS applications and technologies, a comprehensive review of this field is timely. There is a clear need to consolidate the growing body of findings across VNS approaches, so that clinicians and researchers have a unified understanding of what is generally accepted to date. Such a review can highlight the key therapeutic mechanisms by which VNS exerts its effects and help distinguish established benefits from experimental uses. Indeed, with increasing interest in VNS for myriad conditions, additional medical indications are likely on the horizon. Accordingly, this review article aims to synthesize current knowledge across VNS techniques, underscore the mechanisms of action that underlie its therapeutic effects, limitations, and outline future research directions.

## 2 Vagus Nerve Anatomy

The VN arises from the medulla oblongata as a series of rootlets along the postolivary sulcus, between the inferior olive and the inferior cerebellar peduncle. These rootlets converge and exit the cranium via the jugular foramen, inferior to the glossopharyngeal nerve and superior to the cranial root of the accessory nerve. Just distal to the foramen, the VN gives rise to two sensory ganglia: the superior (jugular) ganglion, containing somatic afferent cell bodies, and the inferior (nodose) ganglion, which houses the visceral afferent neurons that project centrally to the nucleus tractus solitarius (NTS) and peripherally to thoracic and abdominal viscera (Berthoud and Neuhuber 2000; Mazzone and Undem 2016).

The VN descends vertically within the carotid sheath in the neck, positioned posterolaterally to the internal carotid artery and medial to the internal jugular vein. Along its cervical course, it gives rise to several key branches. The auricular branch (Arnold's nerve), derived from the superior ganglion, traverses the mastoid canaliculus to provide sensory innervation to the external auditory canal and portions of the auricle (Brodal 2010). At the level of the nodose ganglion, pharyngeal branches emerge and contribute to the pharyngeal plexus, which also receives fibers from the glossopharyngeal nerve and the superior cervical sympathetic ganglia (Kenny and Bordoni 2019).

The superior laryngeal nerve branches from the vagus at the level of the hyoid bone and divides into internal and external branches. The internal branch penetrates the thyrohyoid membrane to innervate the mucosa of the pharynx and supraglottic larynx, while the external branch innervates the cricothyroid muscle, a key regulator of vocal tension (Soriano et al. 2024). Cervical cardiac branches emerge during the nerve descent and extend into the thoracic cavity to contribute parasympathetic fibers to the cardiac plexus, where they modulate the heart rate (HR).

In the thorax, the vagal pathways diverge asymmetrically. The right VN crosses anterior to the subclavian artery and gives off the right recurrent laryngeal nerve, which loops posteriorly around the artery and ascends alongside the tracheoesophageal groove to the larynx. The left VN enters the mediastinum between the left common carotid and subclavian arteries, giving rise to the left recurrent laryngeal nerve at the inferior margin of the aortic arch (Kenny and Bordoni 2019). Both VNs continue posterior to the hilum of the lungs, giving rise to branches that contribute to the pulmonary plexuses. These plexuses provide parasympathetic innervation to the bronchi, pulmonary vessels, and visceral pleura. The nerves then converge to the esophagus, where they form the esophageal plexus. Below this plexus, the fibers coalesce into two major trunks: the anterior vagal trunk, primarily composed of fibers from the left VN, and the posterior vagal trunk, primarily from the right (Kenny and Bordoni 2019; Baquiran and Bordoni 2023).

These trunks traverse the esophageal hiatus of the diaphragm at the T10 vertebral level and enter the abdominal cavity. From there, the vagus distributes preganglionic parasympathetic fibers via the celiac, hepatic, gastric, renal, and superior mesenteric plexuses. The anterior trunk innervates the liver, gallbladder, and anterior stomach, while the posterior trunk sends branches to the posterior gastric wall, pancreas, kidneys, adrenal glands, and intestines up to the mid‐transverse colon. These anatomical connections underpin the VN's critical role in regulating cardiovascular, respiratory, gastrointestinal, hepatic, and renal functions (Berthoud and Neuhuber 2000; Breit et al. 2018).

### 2.1 Afferent and Efferent Pathways

Structurally, the VN is composed of approximately 80% afferent and 20% efferent fibers, encompassing three principal fiber types: A‐, B‐, and C‐fibers. Functionally, Aα‐fibers (*efferent only*) are the largest diameter myelinated axons within the nerve, consisting of α‐motoneurons that innervate pharyngeal and laryngeal muscles (e.g., motor control of swallowing) (Ottaviani and Macefield 2022; Ottaviani et al. 2022). The thinner diameter myelinated Aβ fibers (*both afferent and efferent*) provide somatic sensation to the pharyngeal and laryngeal mucosa (Ottaviani and Macefield 2022), mediate low‐threshold mechano‐sensation in the intrapulmonary airways through slowly adapting receptors (SARs) and rapidly adapting receptors (RARs) that respond to lung inflation and deflation (Mazzone and Undem 2016), and drive the efferent laryngeal bundle controlling laryngeal muscles (Olshansky et al. 2008). Some studies suggest that the VNS therapeutic effects for epileptic seizure control are mediated by activation of Aβ fiber afferents (Hilz 2022; Ellrich 2019; Safi et al. 2016). Additionally, VNS stimulates the laryngeal motor fibers traveling to the recurrent laryngeal nerve via large, myelinated A‐fibers, resulting in side effects such as hoarseness (Ardesch et al. 2010). Finite element modeling indicates that Aβ fibers, which are low‐threshold and superficially located within the VN, are the primary mediator of this side effect (Arle et al. 2016). Aδ‐fibers (*afferents only*) are thin myelinated nociceptive afferents that project from autonomic sensors in the viscera to the nodose and jugular ganglia; they mediate visceral nociception, and are activated by mechanical and chemical irritants in the upper airways, transmitting to the brainstem nuclei and central nervous system (CNS) (Mazzone and Undem 2016).

Smaller myelinated B‐fibers (*both afferent and efferent*) include both baroreceptive afferents and parasympathetic projection efferents. They primarily project preganglionic parasympathetic efferents originating from the dorsal motor nucleus and nucleus ambiguus, synapsing to ganglia in the thoracic and abdominal viscera, thereby modulating cardiac output, bronchomotor tone, and gastrointestinal (GI) motility (Berthoud and Neuhuber 2000; Yuan and Silberstein 2016a). Emerging evidence from Arle et al., supported by simulated finite element modeling, identified that there are “fast” (afferent) B‐fibers associated with aortic baroreceptors and “slow” (efferent) B‐fibers associated with pulmonary side effects (irregular breathing cycle) (Arle et al. 2016). In dogs, electrical stimulation of the VN also revealed two distinct B‐type subtypes with different conduction velocities (fast: 18.0 ± 4.7 m/s, slow: 10.5 ± 1.9 m/s), consistent with Arle et al.'s finite element modeling (Yoo et al. 2013).

C‐fibers (*afferent only*), are the most abundant afferent type, are unmyelinated axons; they are slowly conducting, projecting from visceral afferents that transmit nociceptive, chemosensory, and interoceptive signals from thoracic and abdominal organs to the NTS (Berthoud and Neuhuber 2000; Yuan and Silberstein 2016a). They function as chemosensors and nociceptors, detecting inflammatory mediators, irritants, and tissue damage (Mazzone and Undem 2016; Ottaviani and Macefield 2022; Yuan and Silberstein 2016a).

The cell bodies of vagal afferent fibers reside in two ganglia: the nodose ganglion and the jugular ganglion of the VN. The nodose ganglion contains the majority of the visceral afferent neurons, whereas the smaller jugular ganglion mainly houses somatic afferent (e.g., innervating the external ear via Arnold's nerve and meninges) (Yuan and Silberstein 2016a; Bonaz et al. 2016). Central processes of vagal afferents enter the brainstem and terminate predominantly in the NTS (van Weperen and Vaseghi 2023). Within the NTS, vagal afferent inputs are viscerotopically organized, meaning projections from different organs terminate in specific subregions and are anatomically distinguishable (van Weperen and Vaseghi 2023). In addition, jugular vagal afferents form a distinct somatosensory vagal circuit. These neurons, which innervate somatic structures, project to a region of the dorsolateral medulla known as the paratrigeminal nucleus, located adjacent to the spinal trigeminal nucleus (Kupari et al. 2019; Driessen 2019).

Efferent fibers arise from two nuclei in the medulla: the dorsal motor nucleus of the vagus (DMV) and the nucleus ambiguus. The DMV is the primary source of parasympathetic efferent fibers, projecting to thoracic and abdominal organs. These projections maintain baseline parasympathetic tone and coordinate both excitatory and inhibitory control over visceral functions (Travagli et al. 2003). The nucleus ambiguus contains motor neurons that innervate the pharynx, larynx, and upper esophagus, coordinating swallowing and vocalization via brachial motor fibers of the VN. It also houses cardioinhibitory vagal neurons that project to the heart, contributing to parasympathetic regulation of HR alongside the DMV (Petko and Tadi 2019).

## 3 Stimulation Approaches

VNS is clinically implemented through diverse approaches that can be broadly categorized as invasive and noninvasive methodologies. Invasive VNS (iVNS) employs direct surgical intervention, with the conventional approach involving the implantation of a pulse generator with helical electrodes that directly contact the VN at the cervical level. An alternative invasive technique, vagal‐blocking (vBloc) therapy, utilizes laparoscopically implanted electrodes positioned around the anterior and posterior vagal trunks at the esophagogastric junction, delivering high‐frequency electrical pulses to provide an electrical block (Camilleri et al. 2008). To overcome surgical barriers, several noninvasive and minimally invasive approaches have been developed. These include (a) transcutaneous cervical VNS (tcVNS) targeting the cervical VN, (b) transcutaneous auricular VNS (taVNS) focusing on the auricular branch of the VN, and (c) percutaneous auricular VNS (paVNS) employing minimally invasive needle electrodes near auricular afferents (Farmer et al. 2021).

Beyond these well‐established electrical approaches, alternative modalities have shown growing promise. Focused ultrasound stimulation (FUS) uses acoustic pressure waves to noninvasively activate end organs of the vagus cholinergic anti‐inflammatory pathway (CAP) at the level of the spleen via mechanical interaction with spleen cells (Graham et al. 2020; Zachs et al. 2019). Magnetic VNS (mVNS) employs time‐varying magnetic fields, delivered via implantable coils or magnetothermal nanoparticles, to induce neural excitation without direct electrode contact (Jeong et al. 2022). Furthermore, optogenetic VNS (oVNS) offers a precise experimental approach, where genetically encoded vagal fibers express light‐sensitive ion channels, allowing for fiber‐specific neuromodulation through target photo‐stimulation (Booth et al. 2021).

### 3.1 Electrical Stimulation Approaches

#### 3.1.1 Invasive VNS

**FIGURE 1:**
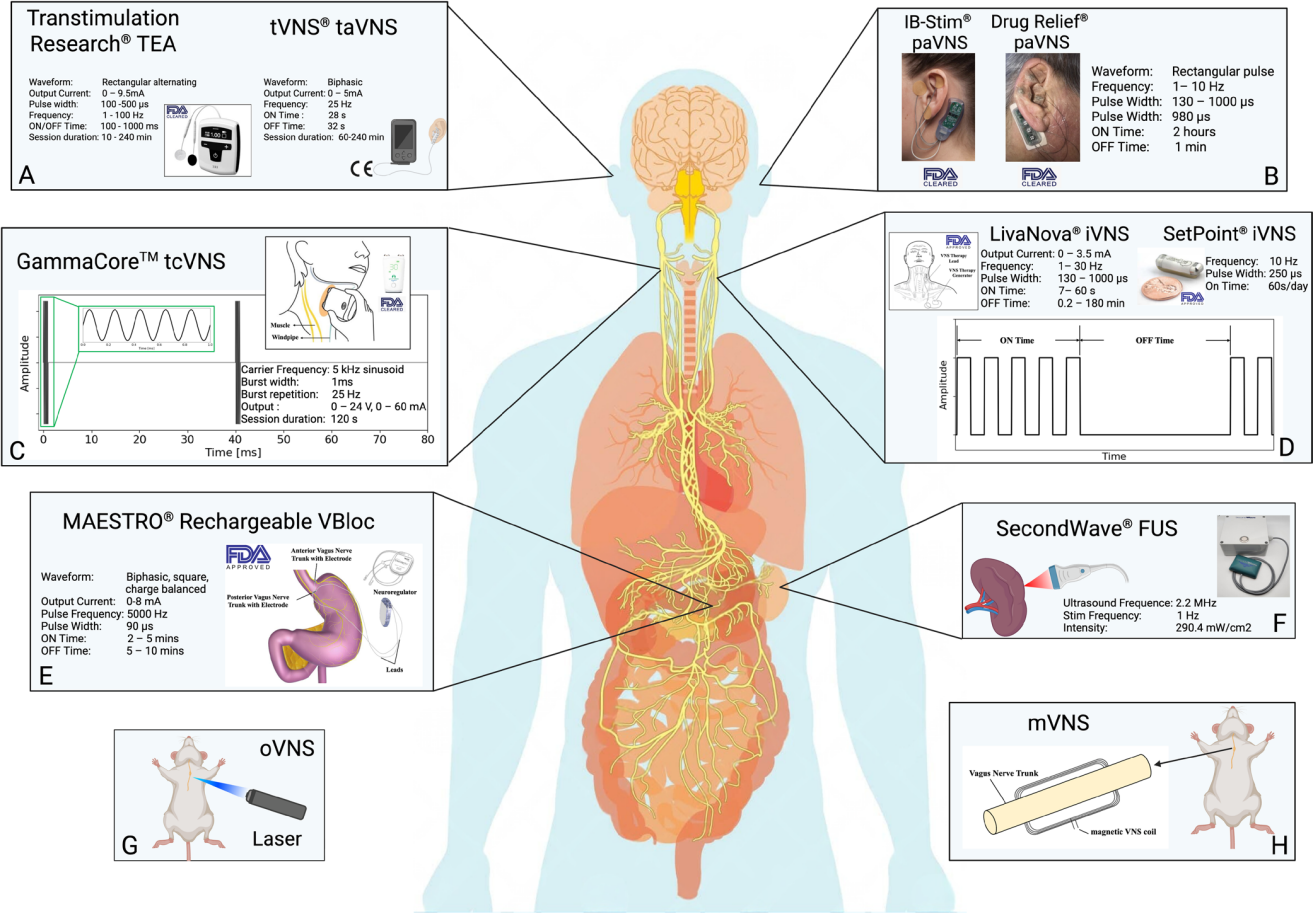
Vagus nerve pathway from the brain through various organs and various vagus nerve stimulation (VNS) technologies. (A) Transcutaneous auricular VNS (taVNS) by Transtimulation Research (FDA‐cleared) and tVNS technologies (CE mark Approved) and typical settings of stimulation parameters. (B) Percutaneous auricular VNS (paVNS) devices and stimulation parameters, Drug Relief (FDA‐cleared) by DyAnsys and IB‐STIM (FDA‐cleared) by Innovative Health Solutions (now NeurAxis). (C) Transcutaneous cervical VNS (tcVNS) by GammaCore (FDA‐cleared) and its stimulation waveforms. (D) Invasive (iVNS) by LivaNova (FDA‐approved) and by SetPoint Medical (FDA‐approved) with programmable parameters. (E) Vagal‐blocking (vBloc) therapy by the MAESTRO Rechargeable system (FDA‐approved) and stimulation parameters. The device is discontinued in the U.S. market. (F) Focused ultrasound stimulation (FUS) by SecondWave Systems for noninvasive stimulation of anti‐inflammatory cellular pathways within the spleen and via feedback circuits to the brain for modulating immune function. (G) Optogenetic VNS (oVNS) uses laser light to activate genetically encoded light‐sensitive ion channels in preclinical studies. (H) Magnetic VNS (mVNS) induces an electric current in the vagus nerve trunk via electromagnetic induction.

iVNS involves the surgical implantation of a stimulation system designed to deliver electrical pulses to the cervical VN. The procedure entails encircling the left VN trunk with a bipolar helical cuff electrode, typically placed at the mid‐cervical level, and routing a subcutaneous lead to a pulse generator implanted in the ipsilateral infraclavicular region (Howland 2014). This dual‐stage approach introduces significant procedural complexity and necessitates long‐term device maintenance, often requiring re‐intervention for battery replacement, depending on stimulation parameters and duration of use (Farmer et al. 2021). Several FDA‐approved iVNS devices are in clinical practice across distinct therapeutic domains. The *VNS Therapy System* (LivaNova, formerly Cyberonics, Figure 1D) was the first such device, receiving FDA approval in 1997 (FDA ref.: P970003) as an adjunct therapy for refractory epilepsy and in 2005 (FDA ref.: 970003/S050) for treatment‐resistant depression (Nemeroff et al. 2006). The system enables patient‐specific optimization through programmable stimulation settings, allowing clinicians to titrate output current, stimulation frequency, pulse width, and duty cycle timing based on individual therapeutic response and tolerability. More recently, the FDA approved two notable VNS applications: the *Vivistim System* (MicroTransponder Inc., FDA ref.: P210007) in 2021 as an adjunct to rehabilitation for improving upper‐limb motor function after ischemic stroke, and the *SetPoint System* (SetPoint Medical, FDA ref.: P240039), a neuroimmune modulation system, in July 2025 for RA (Liu, Russin, et al. 2022).

#### 3.1.2 Transcutaneous Cervical VNS

The cervical VN can also be stimulated transcutaneously by delivering electrical pulses to the cervical VN through surface electrodes applied to the neck. *GammaCore* (ElectroCore Inc., Figure 1C) is a handheld tcVNS device for the acute treatment of migraine and episodic cluster headaches (Silberstein, Mechtler, et al. 2016) and received its FDA clearance initially in 2015 (FDA initial ref.: DEN150048). It was further developed for preventive treatment of cluster headache (FDA ref.: K182369) and migraine headaches in adults (FDA ref.: K191830) and in adolescent patients (FDA ref.: K203546), and most recently for treatment of hemicrania continua and paroxysmal hemicrania in adults (FDA ref.: K221856) in 2021. Two stainless steel round discs function as skin contact surfaces positioned over the cervical region to deliver burst‐patterned electrical stimulation to the underlying VN. The device generates a 5 kHz sinusoidal carrier frequency, delivered in a 1 ms duration, and a 25 Hz burst repetition frequency, providing programmable stimulation intensity of up to 60 mA. Each session lasts for 120 s and can be repeated multiple times per day. Functional magnetic resonance imaging (fMRI) evidence in healthy humans has confirmed that tcVNS activates the ipsilateral NTS and bilateral insula, thalamus, anterior cingulate cortex (ACC), and dorsal lateral prefrontal cortex (Frangos and Komisaruk 2017). To elucidate the mechanism of action, high‐resolution Finite Element Methods models based on MRI‐derived anatomy have been developed to simulate electrical field distribution and nerve action patterns during tcVNS (Mourdoukoutas et al. 2018). Of note, there has been some controversy on the depth of the VN when measured with ultrasound versus examination of cadavers (Staats and Poree 2021; Genovese et al. 2021). The carotid sheath encapsulated VN sits at a variable depth (dependent on transducer probe to skin pressure) of approximately 1.2–2.5 cm (at high‐ and low‐cervical transducer skin pressure due to jugular vein compression) as demonstrated by our group (Mourdoukoutas et al. 2018) and others (Ottaviani et al. 2020) when imaged with ultrasound and the jugular vein is compressed (i.e., the normal use of the *GammaCore* device is to push it down over the carotid artery). In confirmation of this work, our group built a deep learning‐based CNN classifiers to autonomously identify the VN when imaged with noninvasive ultrasound; across *N* = 8 subjects in total, with more than 30,000 scan images, we observed the Vagus depth within the above 1.2–2.5 cm range (Al‐Battal et al. 2021). Genovese and colleagues argued that in their recent trial, the VN is instead at a depth of 2.1–4.69 cm (Staats and Poree 2021; Genovese et al. 2021); while the upper range seems likely in obese patients, the lower range is likely derived from measures when the jugular vein is not compressed (Mourdoukoutas et al. 2018; Lerman et al. 2016). Collectively, tcVNS may contribute to multiple different afferent autonomic regulatory pathways that offer a promising and user‐friendly approach to VN modulation with growing utility across neuropsychiatric and pain‐related conditions.

#### 3.1.3 Transcutaneous Auricular VNS

The auricular branch of the VN (ABVN) provides sensory innervation to specific regions of the external ear, notably the cymba conchae, cavum concha, tragus, and nearby auditory canal skin (Kaniusas et al. 2019). This unique anatomical feature offers a gateway to modulate the VN noninvasively via the ear. Tract‐tracing animal studies and human fMRI confirm that the ABVN projects centrally to the NTS and activates vagal projection areas in the brainstem and forebrain when the cymba concha is stimulated (Frangos et al. 2015). In essence, auricular stimulation recruits vagal afferent pathways similar to cervical VNS, engaging the central autonomic network and neuromodulatory circuits.

taVNS is a completely noninvasive technique in which surface electrodes deliver electrical pulses to the ABVN‐rich area of the outer ear. Common stimulation sites include the tragus and the cymba concha, as these regions are innervated by the auricular vagus branch (Badran et al. 2019). taVNS is typically administered unilaterally using clip electrodes or adhesive gel electrodes attached to the external pulse generator. taVNS provides an accessible, non‐surgical, and affordable option for VN modulation that can be used in outpatient settings or at home (Kim et al. 2022). Over the past decade, the taVNS technique has been explored as a treatment modality in numerous conditions, including refractory depression, epilepsy, chronic pain, tinnitus, stroke rehabilitation, cognitive impairment, and inflammatory disorders (Straube et al. 2015; Verbanck et al. 2021). The *Transcutaneous Electrical Applicator* (TEA) device (Figure 1A; Model number: SNM‐FDC01; FDA ref.: K230526) by Transtimulation Research Inc. is an FDA‐cleared taVNS indicated for relieving functional abdominal pain associated with irritable bowel syndrome (IBS). The two TEA electrode pads were applied bilaterally at the auricular cymba concha (one electrode on each cymba concha). Notably, this device is also cleared to be used as a transcutaneous electrical acustimulator and placed on various acupuncture points to relieve pain associated with sore and aching muscles throughout the body. *Sparrow Therapy System* by Spark Biomedical Inc. is another FDA‐cleared therapy, indicated for reducing opioid withdrawal symptoms (FDA ref. K201873). Both manufacturers indicate that their devices target multiple cranial nerves beyond the VN, including the trigeminal, facial, and glossopharyngeal nerves.

Another device, the *NET‐2000* made by Auri‐Stim Medical Inc., was also cleared by the FDA for the treatment of anxiety, depression, and insomnia (FDA ref.: K060158), though it was labeled as a nerve stimulator rather than a taVNS device (Yap et al. 2020). In Europe, several taVNS devices have CE marks. *tVNS* (formerly *NEMOS*) by tVNS Technologies (Figure 1A) received CE approval for epilepsy and depression in 2010 and for pain in 2012 (Mertens et al. 2018), but it has not secured FDA marketing authorization for clinical use in the United States (Farmer et al. 2021).

#### 3.1.4 Percutaneous Auricular VNS

paVNS is a minimally invasive approach in which fine needle electrodes (~1–2 mm) are percutaneously inserted through the skin of the external ear to stimulate the vagal afferents. Typically, needles are placed in locations on the auricle that are innervated by the ABVN, such as specific points on the antihelix, cymba concha, or tragus region (Kaniusas et al. 2019; Ilfeld et al. 2022). Because the needles penetrate the epidermis, they directly interface with the nerve endings, potentially allowing more focal or efficacious stimulation of the VN fibers compared to the surface electrodes (Kaniusas et al. 2019). With a needle placement procedure performed by clinicians, paVNS is ideal for acute clinical settings (e.g., post‐operative pain control) and patients who struggle with self‐stimulation adherence (Ilfeld et al. 2022). Studies have shown that paVNS can activate parasympathetic outflow (e.g., via pupillary reflex measures) and reduce inflammatory cytokines in clinical scenarios, indicating true vagal‐mediated physiological responses (Kaniusas et al. 2019; Seitz et al. 2022). Several paVNS systems have received regulatory clearance. Innovative Health Solutions (now NeurAxis) obtained FDA De Novo clearance for two paVNS devices: the *NSS‐2 Bridge* for reducing symptoms of opioid withdrawal in patients with substance use disorder (FDA ref.: DEN170018) in 2017, and the *IB‐Stim* for functional abdominal pain associated with IBS (FDA ref.: DEN180057) in 2018. Building on the De Novo precedent, DyAnsys Inc. received 510(k) clearance for *Drug Relief* for opioid withdrawal symptoms (FDA ref.: K173861), followed by two pain‐focused devices: *First Relief* for abdominal pain associated with IBS (FDA ref.: K202940) and chronic, intractable pain from diabetic peripheral neuropathy (FDA ref.: K212859), and *Primary Relief* for post‐operative pain following cesarean section delivery (FDA ref.: K213188) and cardiac surgery (FDA ref.: K221425). Additionally, the *IB‐stim* device by NeurAxis further received FDA clearance for relieving functional abdominal pain associated with IBS for pediatric patients 8 years and older in 2025 (FDA ref.: 252024).

Needle designs are varied among these devices. The *NSS‐2 Bridge* and *IB‐Stim* devices employ a seven‐needle design (a “1‐1‐1‐4” configuration: 3 single‐needle active leads and 1 four‐needle ground array) attached to a small battery‐powered stimulator behind the ear. In practice, clinicians use a pen‐light to transilluminate the auricle and locate vascular landmarks and fine tweezers to place the needles at precise points where vagal fibers lie just beneath the skin (Ilfeld et al. 2022). As a comparison, paVNS devices manufactured by DyAnsys use a four‐needle design with three active stimulation needles and a common ground (Qureshi et al. 2020). Additionally, three‐needle layout through electrical stimulation of auricular acupuncture has also been explored (Sator‐Katzenschlager et al. 2004, 2003). In summary, paVNS provides prolonged and focal vagal stimulation through the ear with minimal invasiveness, bridging the gap between fully implanted VNS and transcutaneous methods.

#### 3.1.5 Vagal‐Blocking (vBloc) Therapy

vBloc therapy represents a distinct approach to vagal modulation that employs high‐frequency electrical stimulation to achieve vagal blockade, opposite to traditional nerve activation. *MAESTRO Rechargeable System* (EnteroMedics, now ReShape Lifesciences, FDA ref.: P130019) is an FDA‐approved invasive vBloc device that utilizes laparoscopic implantation of two bipolar electrodes positioned on the anterior and posterior vagal trunks at the gastroesophageal junction, with leads connecting to a rechargeable pulse generator implanted subcutaneously on the lateral chest wall (Camilleri et al. 2008) (Figure 1E). Unlike conventional VNS, which delivers low‐frequency stimulation to enhance neural activity, vBloc delivers high‐frequency biphasic pulses (typically 5000 Hz) in an intermittent pattern designed to block vagal transmission to the stomach temporarily. This vagal‐blocking device is indicated for weight loss therapy in patients with obesity (Apovian et al. 2017). However, according to *FDA Medical Devices for Weight Loss and Weight Management: What to Know*, the *MAESTRO Rechargeable System* is no longer marketed in the U.S. because of the company's decision. According to the company, removal from the U.S. market was not because of a safety issue (Administration 2022).

#### 3.1.6 Stimulation Parameters and Vagal Fiber Activation

**FIGURE 2:**
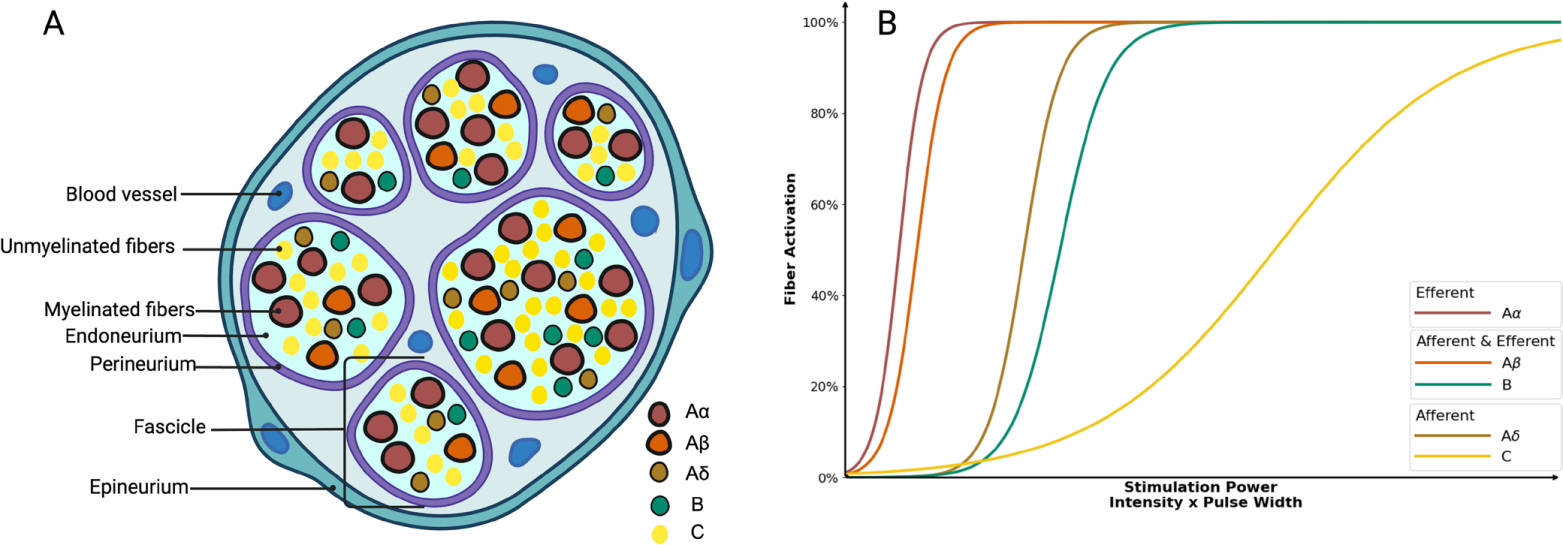
(A) Vagus nerve cross‐sectional composition. (B) Illustration of the relationship between stimulation power and fiber activation of different vagal fibers.

During electrical VNS, recruitment follows fiber size: large‐myelinated A‐fibers activate at the lowest charge density, followed by small‐myelinated B‐fibers at higher charge density, and finally non‐myelinated C‐fibers only at the highest charge density (Figure 2). Charge density is determined directly by current, pulse width, and inversely by electrode area. For example, in a canine iVNS study with 0.3 ms pulse width, vagal A‐fibers were excited at 0.37 mA, B‐fibers at 1.6 mA, and C‐fibers not until 17 mA (Yoo et al. 2016). In smaller animals such as a rat iVNS study with 0.1 ms pulse width, the stimulation intensity needs to be ~20 times the lowest A‐activation threshold to recruit B‐fibers and ~60–80 times the threshold to recruit C‐fibers (McAllen et al. 2018). The VN of humans and pigs has similar amounts of fibrous tissue and a comparable diameter (Settell et al. 2020). In domestic pig experiments using 0.2 ms biphasic pulses for invasive VNS, Nicolai et al. established fiber recruitment thresholds of 0.30 mA for Aα/Aβ fibers and 1.67 mA for Aδ/B‐fibers (Nicolai et al. 2020). Consistent with Berthon et al., both studies failed to recruit C‐fibers even at their maximum stimulation intensity (3 and 2.5 mA, respectively) (Berthon et al. 2024). However, in a human iVNS study involving two epilepsy patients, Macefield et al. found that a clinical iVNS device operating at < 1 mA could successfully activate C‐fibers. This discrepancy is likely attributable to their unique micro‐neurography technique, which demonstrates greater sensitivity by directly interfacing with axons, compared to the cuff electrodes that are positioned around the epineurium in animal studies (Patros et al. 2024). This finding advances our understanding that C‐fiber activation may be partially involved in the therapeutic mechanisms of iVNS treatment for epilepsy and potentially other neurological conditions.

The stimulation intensity needs to be carefully fine‐tuned to achieve therapeutic effects. For example, ensuring B‐fiber modulation to initiate bradycardia effects is crucial for some cardiovascular diseases (Qing et al. 2018; Ardell et al. 2017). Likewise, stimulation intensity above the C‐fiber activation threshold can disrupt normal respiratory patterns and potentially induce apnea (Chang et al. 2020). Fiber activation follows the strength‐duration curve; therefore, both stimulation intensity and pulse width are crucial parameters in maintaining stimulation within safe charge density while ensuring adequate neural recruitment for therapeutic efficacy. Importantly, depending on the individual and species, the activation amplitude required to produce a given nerve response varies widely, causing the application of the same or linearly scaled VNS parameters across species to produce highly variable responses, highlighting the need for accounting for these differences during VNS parameter selection and clinical translation (Musselman et al. 2025).

Additionally, stimulation frequency is another crucial parameter that drives the excitatory or inhibitory nature of the nerve activity. Electrical stimulation at very low frequency (1–5 Hz) activates vagal fibers in strict size order, and each pulse acts independently, except that the nociceptive C‐fibers have an activity‐dependent effect, such that their conduction velocities slow down with stimulation above 0.25 Hz (Serra et al. 1999). At conventional VNS rates (~20–30 Hz), recruitment remains intensity‐dependent in the same order. This frequency range may also provide better therapeutic benefits compared to low‐frequency stimulation for seizure control (Ben‐Menachem et al. 1994), possibly because of its consistent and repeated activation of locus coeruleus (LC) (Farrand et al. 2023); it is purported to lead to a higher level of norepinephrine (NE) release. This frequency‐dependent efficacy is further demonstrated by its bradycardia effect, while the low‐frequency VNS fails to reduce heart rate, even with successful B‐fiber activation (Ahmed et al. 2021). Suprathreshold 10–50 Hz stimulation blocks C‐fiber activity by perpetual induction of a refractory period (due to the fact that C‐fibers remain unable to entrain firing above 1–2 Hz); this approach may be leveraged to provide pain relief for various chronic pain conditions (Zhang, Chen, et al. 2024). In stark contrast, kilohertz (kHz)‐frequency stimulation induces conduction block by trapping sodium channels in refractory states across all fibers. Continuous kHz trains suppress A/B conduction and recruit C‐afferents via integrative charging at suprathreshold stimulation intensity (Chang et al. 2022). Conversely, subthreshold high‐frequency trains can generate action potentials via integrative properties, similar to temporal summation at the synaptic levels (Boulet et al. 2016). Current clinical use iVNS at 20–30 Hz primarily recruits A‐ and B‐fibers (Carron et al. 2023). When accessing the ABVN, it should be noted that it is composed of Aβ, Aδ, and C‐fibers; at the auricular site, VNS may selectively activate these fibers depending on the stimulation intensity (Wang, Li, et al. 2021). For tcVNS, results from computational studies indicate that when pressure is applied to the device over the carotid artery, tcVNS preferentially activates A‐ and B‐fibers within the cervical VN, while C‐fibers remain below the excitation threshold (Mourdoukoutas et al. 2018). In both cases (cervical or auricular), transcutaneous neural stimulation may result in multiple adjunctive or inhibitory neural circuit activation in addition to the on‐target vagal fiber activation, which produces diffuse stimulation fields that co‐stimulate adjacent non‐vagal structures for both tcVNS (Yap et al. 2020), and taVNS (Kaniusas et al. 2019). Additionally, auricular VNS targets the purely afferent ABVN, which contains five to six times fewer Aβ fibers compared to the cervical vagus trunk, and motor efferent fibers cannot be directly recruited through auricular stimulation (Butt et al. 2020). Thereby, by definition, all transcutaneous VNS cannot be considered classical dedicated vagal nerve stimulation as iVNS. Taken together, these findings underscore that optimal electrical VNS therapy depends on precise tuning of stimulation parameters to selectively engage A‐ and B‐fibers for therapeutic efficacy while minimizing C‐fiber‐mediated unwanted side effects (Krahl et al. 2001; Yang and Phi 2019).

#### 3.1.7 Safety, Tolerability, and Efficacy

For safety concerns, invasive VNS requires surgical implantation, with risks including infection, hematoma, cardiac events during surgery, and potential vocal cord paralysis lasting months. In contrast, transcutaneous approaches (tcVNS/taVNS) and percutaneous stimulation (paVNS) avoid these surgical dangers, with serious adverse events exceedingly rare (Redgrave, Day, et al. 2018). Percutaneous methods cause only minor local irritation at needle sites (Szeles et al. 2021).

Tolerability is another aspect to compare that relates to how comfortably patients can endure the therapy's side effects. iVNS commonly produces stimulation‐induced side effects like voice hoarseness, throat discomfort, coughing, shortness of breath, or tingling sensations during nerve activation (Ben‐Menachem et al. 2015). These effects are usually not dangerous but can be bothersome enough that stimulation parameters must be lowered or temporarily deactivated in some patients (Ben‐Menachem et al. 2015). By contrast, tcVNS and taVNS are generally well tolerated. A systematic review of tcVNS reported skin irritation at the electrode site as the most common side effect (18.2% of users) and found that only about 2.6% of participants discontinued treatment due to side effects (Redgrave, Day, et al. 2018). Other mild symptoms with tcVNS/taVNS can include a tingling or pressure sensation at the stimulation site, or slight dizziness, but serious or systemic side effects are uncommon (Redgrave, Day, et al. 2018). The paVNS technique is also reported to be well tolerated: any adverse events tend to be mild and transient (Szeles et al. 2021).

Efficacy, on the other hand, varies across these VNS modalities. iVNS has proven the most robust and well‐established benefits in certain disorders. In refractory epilepsy, about 45%–65% of patients achieve at least a 50% reduction in seizure frequency after months of iVNS therapy (Toffa et al. 2020). iVNS also yields approximately 25%–35% improvements in depression severity scores in treatment‐resistant depression over time (Toffa et al. 2020). In contrast, the noninvasive VNS modality shows promising but more variable results. For example, tcVNS has demonstrated clinical benefits in headache disorders (Li et al. 2022). Likewise, taVNS has been tested in epilepsy and depression: in one controlled trial, 41% of patients receiving taVNS had a seizure reduction at 8 weeks, compared to 27.5% in the sham group (Austelle et al. 2024); studies in depression have reported modest improvements on depression scales with taVNS versus placebo, though results have varied by outcome measure (Austelle et al. 2024). paVNS has also demonstrated therapeutic benefits: one clinical study in chronic pain reported that nearly 59% of patients achieved at least a 50% reduction in chronic back pain intensity after 6 weeks of paVNS treatment (Szeles et al. 2021). However, while these noninvasive and minimally invasive approaches clearly engage vagal pathways and have shown benefits in pilot studies, their overall efficacy is still being defined. To date, fewer large‐scale trials and inconsistent methodologies make it harder to quantify the success rates of tcVNS, taVNS, and paVNS with the same confidence as iVNS (Toffa et al. 2020). In summary, iVNS remains the gold standard with well‐documented efficacy in certain conditions, whereas the noninvasive modalities, though considerably safer, exhibit encouraging but as yet less proven efficacy and will benefit from further large trials to fully establish their therapeutic potential (Ben‐Menachem et al. 2015; Toffa et al. 2020).

### 3.2 Non‐Electrical Stimulation Approaches

#### 3.2.1 Focused Ultrasound Stimulation

FUS is an emerging VNS technique that does not rely on direct electrical currents. FUS delivers acoustic energy noninvasively through the skin to specific downstream targets of the vagal nerve using low‐intensity pulsed ultrasound. At low intensities, the mechanical effects of oscillating pressure waves produced by ultrasound pulses induce an acoustic radiation force. It is thought that this causes neuronal membrane deformation and activation of mechanosensitive ion channels that subsequently depolarize nerve fibers. Conversely, at higher intensities, the thermal effect of the applied ultrasound energy becomes more significant, where the energy of the waves increases the temperature within the ultrasound focus area, in turn dampening neural excitability by disabling ion channel function (Goyal et al. 2024). Recent clinical and preclinical investigations have demonstrated that FUS targeting of the spleen with low intensity and low‐frequency ultrasound can effectively modulate the CAP pathway focally at the splenic level (potentially without systemic activation of other vagal pathways) (Cotero et al. 2019) (Figure 1F). In a human study, healthy subjects receiving 3‐min splenic FUS showed a significant reduction in tumor necrosis factor alpha (TNF‐α) production upon ex vivo endotoxin challenge (Graham et al. 2020), while an arthritis mouse model demonstrated reduced disease severity after daily noninvasive ultrasound stimulation targeting the spleen (Zachs et al. 2019). FUS presumably excites nerves projecting within the spleen. However, many non‐neural cells have been shown to be readily activated by ultrasound stimulation, whereas direct excitation of nerves has required much higher and potentially damaging intensities for excitation (Rodríguez‐Meana et al. 2024; Guo et al. 2022; de Lucas et al. 2020; Uddin and Komatsu 2020).

Ultrasound neuromodulation at low intensity appears to be mediated via specific mechanically sensitive ion channels or cellular receptors, in which a variety of different types of cells within the spleen can contribute to the anti‐inflammatory response. Different ion channels in peripheral and central neurons appear to confer ultrasound sensitivity, while supporting cell types (such as astrocytes and glia) also appear to respond to ultrasound stimulation (Yoo et al. 2022; Cotero et al. 2022; Oh et al. 2019; O'Reilly 2024). These ultrasound‐sensitive channels have also been found on neuronal soma, as well as synaptic structures, suggesting location‐specific stimulation effects (Cotero et al. 2019, 2022, 2020; Tyler et al. 2008). However, many non‐neural cells have been shown to be readily activated with ultrasound stimulation, whereas direct excitation of nerves has required much higher and potentially damaging intensities for excitation (Rodríguez‐Meana et al. 2024; Guo et al. 2022; de Lucas et al. 2020; Uddin and Komatsu 2020). Furthermore, the main nerve bundle projecting into the spleen is located at the hilum or center of the end‐organ, whereas ultrasound stimulation of different locations across the spleen can drive equivalent anti‐inflammatory effects (Cotero et al. 2019; Zanos et al. 2023). Therefore, FUS likely drives anti‐inflammatory effects in the spleen via mechanical activation of non‐neural cells, such as immune cells and spleen glial cells.

Overall, low‐intensity FUS offers the advantage of noninvasive and potentially precise activation of different types of cells that can be leveraged to treat inflammatory disorders and other health conditions. The therapeutic effect of FUS has only recently been translated to initial clinical pilot studies and is still under investigation with a limited set of stimulation settings. Further research is needed to investigate a broader range of stimulation parameters for FUS that could drive differing physiological and immune effects in the body, depending on the type of cells activated for various clinical applications.

#### 3.2.2 Magnetic Stimulation

mVNS employs time‐varying magnetic fields to induce electric currents in vagal nerve fibers via electromagnetic induction. Multiple technical implementations of mVNS have been explored, encompassing both invasive and noninvasive approaches. For example, implantable microcoil stimulators can be placed adjacent to the nerve to deliver focal magnetic pulses, selectively activating vagal afferent fibers and producing neuromodulatory effects (Jeong et al. 2022) (Figure 1H). In addition, magnetothermal strategies employ injectable magnetic nanomaterials (e.g., iron‐oxide nanoparticles within a biocompatible hydrogel) that transduce an external oscillating magnetic field into localized thermal or mechanical stimuli, thereby remotely exciting the VN (Bao et al. 2023; Lu et al. 2023). In rats, mVNS has enabled focal magnetic stimulation of the cervical vagus, selectively recruiting afferent fibers and potentially mitigating bradycardia side effects (Jeong et al. 2022).

#### 3.2.3 Optogenetic Stimulation

oVNS is a neuromodulation technique that uses genetically encoded light‐sensitive ion channels (opsins) to precisely control vagal nerve activity. After allowing opsin expression in the vagal fibers, an implanted optical interface delivers light pulses to the nerve. Excitatory opsins will depolarize and fire action potentials in opsin‐expressing vagal fibers upon light stimulation, whereas inhibitory opsins can hyperpolarize those fibers and silence their activity (Cela and Sjöström 2019) (Figure 1G). This optogenetic approach enables fiber‐type selectivity and temporal precision far beyond conventional electrical VNS.

Selectivity is usually achieved through two strategies: anatomical targeting and genetic targeting. In anatomical targeting, viral vectors (e.g., Lentiviral vectors carrying PRSx8‐ChIEF promoters) are injected into specific brainstem nuclei, such as the DMV, which selectively transduces efferent vagal preganglionic neurons while leaving afferent vagal fibers unaffected (Booth et al. 2021). Genetic targeting uses Cre‐Lox technology to exploit the molecular identity of a specific neuronal population. By crossing Chat‐ires‐Cre mice with mice carrying Cre‐dependent channelrhodopsin‐2 (ChR2, a light‐activated protein), researchers can direct ChR2 expression to cholinergic efferent neurons, ensuring that only these fibers respond to light stimulation. Similarly, Vglu2‐ires‐Cre mice enable selective ChR2 expression in glutamatergic afferent only neurons (Tanaka et al. 2021). Further specificity can be achieved by targeting distinct afferent subtypes based on receptor expression. Npy2r (expressing the neuropeptide Y receptor Y2 protein) neurons are largely slow‐conducting C‐fibers, while P2yr1 (expressing the purinergic receptor P2Y1 protein) neurons are largely fast‐conducting A‐fibers. Selective optogenetic activation of these subtypes produces opposing respiratory effects: Npy2r + fiber stimulation triggers rapid shallow breathing, whereas P2ry1+ fiber stimulation induces apnea, demonstrating that oVNS can precisely control even functionally distinct subpopulations within the same sensory pathway (Chang et al. 2015).

Notably, this optogenetic finding contrasts with conventional electrical VNS observations. Yao‐Chuan et al. found that C‐fiber activation correlated with breathing interval change, such that weak C‐fiber activation was associated with slower breathing during electrical VNS, whereas strong C‐fiber activation produced apnea, opposite to the rapid breathing observed with selective Npy2r + (C‐fiber) optogenetic stimulation (Chang et al. 2020). This discrepancy likely arises because conventional electrical stimulation follows a strength‐duration recruitment principle: larger A‐fibers activate at lower intensities before smaller C‐fibers. Consequently, C‐fiber activation during electrical VNS most likely occurs in combination with substantial A‐fiber co‐activation rather than in isolation. Therefore, fiber‐selective approaches are essential for researchers to understand underlying mechanisms and control confounding factors in VNS therapy.

Both anatomic and genetic strategies allow subsequent light stimulation to activate only the transduced fiber population while leaving other vagal fibers unaffected. Furthermore, light can be pulsed on the millisecond scale, yielding tightly time‐locked neural responses (Booth et al. 2021; Chang et al. 2015). Collectively, the features of fiber selectivity and millisecond‐level temporal resolution enable oVNS to dissect the VN's complex architecture with a resolution impossible to achieve through electrical stimulation, making it an essential tool for understanding which specific vagal pathways mediate particular physiological and therapeutic effects.

#### 3.2.4 Summary of Non‐Electrical VNS Approaches

Non‐electrical VNS techniques represent an expanding frontier in neuromodulation that avoids direct electrical current delivery. Each of these modalities presents unique technical advantages, ranging from anatomical selectivity to wireless control and cell‐type precision, that may overcome the limitations of traditional electrical stimulation. While none of these approaches currently have FDA‐cleared devices for clinical use, their ongoing development highlights promising avenues for future VNS applications.

## 4 Mechanisms of Action

### 4.1 Central Nervous System

#### 4.1.1 Brainstem Activation and Neuromodulator Release

VNS excites vagal afferent fibers that project to the NTS in the brainstem. Neurons of the NTS relay visceral information from the VN upward through multiple pathways. The NTS sends broad projections within the brainstem and toward higher centers, including the parabrachial nucleus (PBN), raphe nuclei, LC, and ascending forebrain structures such as the amygdala, hypothalamus, thalamus, and cerebellum. These anatomical pathways link peripheral organ signals to the brain's arousal, emotion, and homeostatic network in a direct way. Through these connections, visceral inputs can rapidly influence central autonomic control, stress response, and emotional processing (Breit et al. 2018).

Crucially, vagal afferent activity engages key neuromodulatory systems in the brain. One major pathway is from the NTS to the LC. The LC is the brain's principal source of NE and broadcasts signals throughout the cortex, hippocampus, thalamus, and amygdala. VNS input via the NTS can activate LC neurons, leading to elevated NE release in widespread regions. This burst of NE enhances arousal and modulates memory and attention circuits (Manta et al. 2009; Gargus et al. 2025).

Vagal afferents also influence the brain's serotonergic system. The NTS projects to the dorsal raphe nucleus (DRN), the major source of serotonin (5‐HT) in the forebrain. Chronic VNS in rats has shown that extended NE production can indirectly regulate the release of 5‐HT in the DRN, ultimately enhancing 5‐HT transmission in the brain (Manta et al. 2009). Another important NTS projection connects to the Nucleus Basalis of Meynert (Gratwicke et al. 2013), a basal forebrain region responsible for producing acetylcholine (ACh) for the prefrontal cortex (PFC), hippocampus, and amygdala (Hulsey et al. 2016). The ACh release enhances cortical plasticity, attention, and memory processing (Gargus et al. 2025). Activation of vagal pathways has been shown to recruit basal forebrain cholinergic circuits, resulting in increased ACh release in cortical regions (Lehner et al. 2019).

Projection from the NTS also connects to the paraventricular nucleus (PVN), which plays a key role in regulating corticotropin‐releasing hormone (CRH) as part of the hypothalamic–pituitary–adrenal (HPA) axis (Pavlov et al. 2003). This link provides the VN a pathway to modulate the neuro‐hormonal anti‐inflammatory responses in the body (Gargus et al. 2025; Pavlov et al. 2003), even if bilateral efferent vagal pathways are interrupted (Rosas‐Ballina et al. 2008). Lastly, the VN can influence hippocampal functions and the production of brain‐derived neurotrophic factor (BDNF) via connections originating from the NTS, LC, and DRN (Gargus et al. 2025; Pavlov et al. 2003).

**FIGURE 3:**
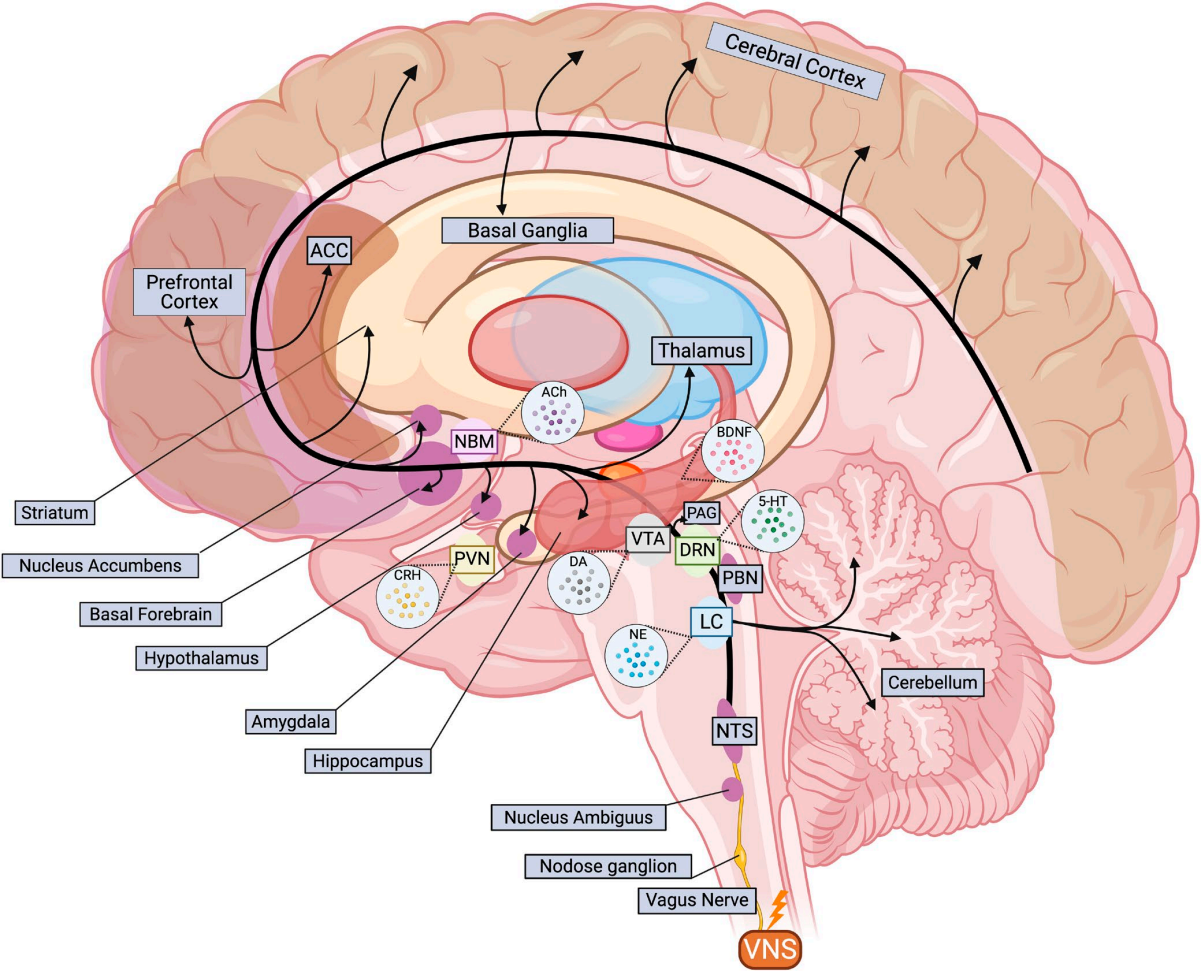
Schematic illustration of VN pathways and their neuromodulatory effects in the brain. VNS initiates ascending afferent signals from the nodose ganglion to the nucleus tractus solitarius (NTS), subsequently activating critical brainstem nuclei including locus coeruleus (LC), dorsal raphe nucleus (DRN), ventral tegmental area (VTA), hippocampus, paraventricular nucleus (PVN), and nucleus basalis of Meynert (NBM). These nuclei release key neuromodulators—norepinephrine (NE), serotonin (5‐HT), dopamine (DA), brain‐derived neurotrophic factor (BDNF), corticotropin‐releasing hormone (CRH), and acetylcholine (ACh). Major target structures include the cerebellum, the parabrachial nucleus (PBN), the periaqueductal gray (PAG), the thalamus, the amygdala, the hypothalamus, the basal forebrain, the nucleus accumbens, the basal ganglia, the striatum, the prefrontal cortex, the anterior cingulate cortex (ACC), and the cerebral cortex, collectively orchestrating therapeutic effects of VNS across diverse conditions.

Taken together, stimulation of the vagal afferent sets off a chain reaction: NTS activation leads to the release of NE from the LC, 5‐HT from DRN, ACh from basal forebrain cholinergic groups, CRH from PVN, and BDNF from the hippocampus, which collectively modulate widespread CNS activity (Figure 3) (Gargus et al. 2025; Hulsey et al. 2016; Olsen et al. 2023; Dorr and Debonnel 2006). This neurochemical mobilization underlies many of the arousal‐ and plasticity‐enhancing effects of VNS, providing a mechanistic link between peripheral vagal stimulation and the central neuromodulator network.

#### 4.1.2 Neural Plasticity and Synaptic Remodeling

VNS creates a pro‐plasticity environment in the brain by engaging the neuromodulatory systems described above. NE and ACh are well‐known facilitators of synaptic plasticity and cortical reorganization, and their VNS‐mediated release is a key driver of neural changes (Hays et al. 2013). Indeed, VNS has been shown to increase indicators of synaptic plasticity at the molecular and cellular levels (Olsen et al. 2023; Bowles et al. 2022).

Research has demonstrated that pairing VNS with motor rehabilitation or skill training enhances the reorganization of the sensorimotor cortex. In animal models, VNS delivered during rehabilitation exercises drives robust expansion of movement representation in the primary motor cortex (Hulsey et al. 2016). Rats receiving VNS paired with forelimb training developed significantly larger motor cortex maps for the trained forelimb, indicating a strengthening cortical representation of motor events (Hulsey et al. 2016; Porter et al. 2012). This VNS‐enhanced cortical plasticity depends on cholinergic activity: if the basal forebrain cholinergic neurons are lesioned, the VNS no longer boosts motor map reorganization (Hulsey et al. 2016), which supports the idea that VNS can directly amplify circuit remodeling of the damaged brain (Bowles et al. 2021).

In addition, VNS also promotes plasticity in circuits related to emotion and memory. A striking example is the enhancement of fear extinction by VNS. Extinction of conditioned fear requires plastic changes in the amygdala and medial PFC (mPFC). In rodent studies, pairing VNS with exposure to a feared cue substantially accelerates extinction, evidenced by a faster reduction in fear responses compared to controls (Peña et al. 2014). Neurophysiologically, VNS pairing transforms the plasticity in the infralimbic prefrontal‐amygdala pathway: typically, fear conditioning followed by extinction training induces long‐term depression (LTD) in this pathway, but if VNS is coupled with the extinction trials, it instead produces long‐term potentiation (LTP) between the infralimbic cortex and basolateral amygdala. Thus, VNS can change limbic circuits toward forming new, non‐fearful associations; it is a form of adaptive remodeling with therapeutic potential in anxiety, depression, or posttraumatic stress disorder (PTSD) (Peña et al. 2014). More broadly, studies in both animals and humans indicate that VNS paired with training enhances memory retention and cortical reorganization in cognitive circuits as well (Olsen et al. 2023). In our own work, VNS resulted in greater attention as measured by fMRI and faster reaction time when anticipating and viewing aversive imagery (Lerman et al. 2022), while we also observed improvement in recall post‐encoding (Klaming et al. 2022). This ability of VNS to promote targeted synaptic plasticity underlies many of its applications in neurorehabilitation and neuropsychiatry. Furthermore, by decreasing peripheral inflammation (see Section 4.2.1), VNS might improve neuroplasticity and neurotransmitter availability since proinflammatory cytokines are known to lower 5‐HT and glutamate function in the brain (Miller et al. 2013).

**FIGURE 4:**
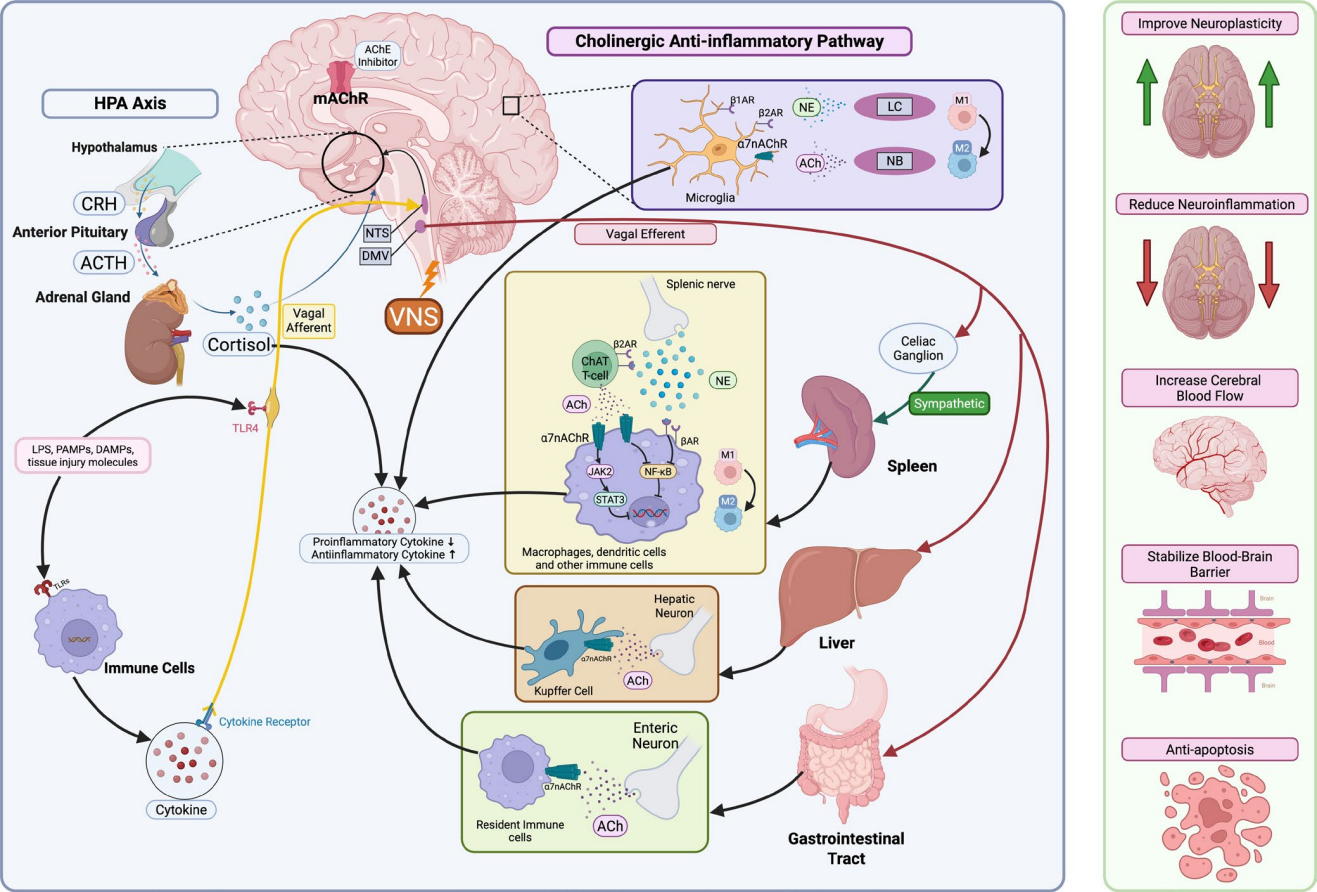
VNS‐mediated anti‐inflammatory and protective effects. Upon immune challenge, the activation of TLRs triggers activated macrophages and other immune cells to release inflammatory mediators, including cytokines. These cytokines subsequently activate vagal afferents, which modulate the hypothalamic–pituitary–adrenal (HPA) axis and stimulate cortisol release. This cascade ultimately suppresses proinflammatory cytokine production while promoting anti‐inflammatory cytokine secretion. Vagal efferent fibers exert anti‐inflammatory actions via the cholinergic anti‐inflammatory pathway. Within the spleen, norepinephrine (NE) released by the splenic nerve and acetylcholine (ACh) released by ChAT‐positive T cells inhibit macrophage activation via β‐adrenergic receptors (βAR) and α7 nicotinic acetylcholine receptors (α7nAChR), downregulating JAK2‐STAT3 and NF‐κB pathway, thereby shifting macrophages toward an anti‐inflammatory M2 phenotype. Hepatic and enteric neurons similarly reduce inflammation by ACh‐mediated modulation of Kupffer cells and resident immune cells. Additionally, VNS also directly influences microglia in the brain. NE and ACh release lead to activation of the cholinergic and noradrenergic anti‐inflammatory pathway, shifting microglia from proinflammatory M1 to neuroprotective M2 phenotype. The anti‐inflammatory effect of VNS exerts multifaceted effects, including improving neuroplasticity, reducing neuroinflammation, increasing cerebral blood flow, stabilizing the blood–brain barrier, and anti‐apoptosis. AChE, acetylcholinesterase; ACTH, adrenocorticotropic hormone; CRH, Corticotropin‐releasing hormone; DAMPs, damage‐associated molecular patterns; DMV, dorsal motor nucleus of the vagus; LPS, lipopolysaccharide; mAChR, muscarinic acetylcholine receptor; NTS, nucleus tractus solitarius; PAMPs, pathogen‐associated molecular patterns.

VN activity also indirectly influences resident immune cells of the CNS, particularly microglia. ACh release following VNS activates the cholinergic anti‐inflammatory pathway via α7nAChR, shifting microglia from proinflammatory M1 to neuroprotective M2 phenotype (Chen et al. 2022; Suzuki et al. 2006). Additionally, NE inhibits abnormal microglial activation and reduces inflammatory reaction via β‐adrenergic receptors (Zou et al. 2021; Heneka et al. 2010). Thus, VNS may engage both cholinergic and noradrenergic pathways to modulate microglial function, as hypothesized by Kaczmarczyk et al. (2018) (Figure 4). Preclinical studies demonstrate that vagal stimulation can suppress the brain production of cytokines such as TNF‐α, Interleukin‐1 beta (IL‐1β), and Interleukin‐6 (IL‐6) during systemic inflammatory challenges (Jiang et al. 2014). Importantly, proinflammatory cytokines can decrease blood–brain barrier (BBB) integrity by disrupting tight junction proteins that normally seal connections between brain endothelial cells (Gryka‐Marton et al. 2025). This leads to increased BBB permeability, allowing harmful blood components and immune cells to infiltrate the brain and cause neuroinflammation in conditions like multiple sclerosis, stroke, and neurodegenerative diseases (Gryka‐Marton et al. 2025). Suppressing central inflammation helps preserve BBB integrity and limit cytokine‐induced neurovascular damage (Jin et al. 2023).

#### 4.1.3 Behavioral and Cognitive Modulation

##### 4.1.3.1 Effects on Learning, Memory, and Attention

VNS also has notable effects on cognitive processes, including learning, memory, and attention. VNS alters brain neurotransmitters, such as γ‐aminobutyric acid (GABA), glutamate, 5‐HT, dopamine (DA), and NE, thereby affecting excitability in memory‐associated pathways. These alterations enhance alertness, promote synaptic transmission, and reinforce memory‐related processes (Olsen et al. 2023). For example, in vivo studies show that VNS can increase hippocampal LTP and brain BDNF expression, strengthening memory encoding pathways (Olsen et al. 2022). Consistent with this, VNS also demonstrates improved learning and recognition memory in animal models (Jung et al. 2024; Altidor et al. 2021). Human studies have reported improved attention, arousal, memory, and decision‐making performance in patients receiving VNS therapy (Olsen et al. 2023). Notably, neuroimaging evidence indicates that VNS engages fronto‐parietal and attention networks (Han, Shim, et al. 2024), which are responsible for sustained attention, novelty detection, working memory, and problem‐solving (Vincent et al. 2008). This implies that VNS can alter the brain toward an “alert” state, recruiting dorsal attention and central executive networks to enhance cognitive processing. Even in a sleep‐deprived state, VNS has demonstrated improved reaction time and attention (McIntire et al. 2021).

##### 4.1.3.2 Effects on Emotional Regulation and Mood Disorders

In addition, VNS exerts a profound influence on the limbic circuits and large‐scale networks involved in emotion and mood regulation. Owing to its impact on key regions like the amygdala, hippocampus, ACC, and PFC (Nemeroff et al. 2006), VNS has been explored as a treatment for refractory depression, anxiety, and PTSD. Imaging studies indicate that VNS can tone down hyperactive limbic areas often implicated in mood disorders (Ressler and Mayberg 2007). In epilepsy patients, positron emission tomography (PET) scans during acute VNS demonstrated a significant reduction in metabolic activity in the amygdala, hippocampus, and cingulate gyrus—all regions tied to mood regulation (Nemeroff et al. 2006). Similarly, fMRI with VNS showed deactivation of limbic and temporal lobe structures, accompanied by mood‐enhancing effects (Kraus et al. 2007). Further, our own work demonstrated an acute reduction in salience network activity when subjects were challenged with painful stimuli (Lerman et al. 2019). This suggests that VNS may normalize the overactivity of limbic regions seen in depression or PTSD, thereby restoring emotional balance; however, it is likely that learned emotional regulation must be paired with an exogenous therapy, such as Prolonged Exposure (PE) therapy as shown by Souza et al. (2019) and now with emerging results from Powers et al. (2025). Similarly, VNS therapy was found to modulate the brain default mode network connectivity coupled with regions like the precuneus, orbital frontal cortex, parahippocampus, insula, and dorsal ACC, and their changes correlated with a reduction in Hamilton Depression scores (Fang et al. 2016). By suppressing overactive limbic activity while modulating default mode network functional connectivity, VNS may facilitate the shift from internally focused, self‐referential processing toward a more externally engaged, emotionally balanced state.

Beyond depression, VNS shows promise in modulating fear and stress‐related circuits relevant to PTSD and anxiety. The ventromedial PFC (vmPFC)—amygdala circuit, crucial for the extinction of fear, is specifically strengthened by VNS (Szeska et al. 2020). In a human study, participants receiving VNS during extinction training showed reduced return of fear responses, indicating that vagal stimulation can facilitate the formation of safety memories. Thus, across mood and anxiety disorders, VNS modulates limbic and paralimbic networks, including the amygdala‐hippocampal complex, the salience network, and medial prefrontal regions, to promote better emotional regulation (Szeska et al. 2020).

##### 4.1.3.3 Effects on Reward Processing and Decision‐Making Circuits

VNS also interacts with the brain's reward and decision‐making circuitry, which involves the mesolimbic DA system and prefrontal‐striatal networks. Long‐term VNS in rats elevates extracellular DA levels in the PFC and nucleus accumbens despite reducing DA neuron firing in the ventral tegmental area (VTA) (Manta et al. 2013). This supports a model in which VNS can upregulate mesolimbic DA transmission, a key chemical signal for reward and motivation. Similarly, human fMRI has shown that VNS activates the NTS and dopaminergic midbrain region as well as the striatum (Neuser et al. 2020). Although the precise mechanism is still being unraveled, it is widely accepted that VNS triggers a cascade from the brainstem that ultimately boosts catecholamine release (e.g., NE and DA) in higher circuits (Gargus et al. 2025; Manta et al. 2013).

Beyond nuclei neurotransmitter effects, VNS produces widespread effects on the brain's reward‐related networks. Functional imaging reveals that multiple key regions in the reward network are modulated by VNS, including the PFC, ACC, insula, amygdala, and striatum (Conway et al. 2012; Wang et al. 2018). The insula is thought to integrate internal body states with reward value, and VNS‐driven insular activation may thus influence how motivational significance is assigned to stimuli (Chen et al. 2021; Teckentrup and Kroemer 2024). Changes in prefrontal‐striatal connectivity with VNS suggest a tuning of the circuit that underlies reward evaluation and decision‐making (Wang et al. 2018; Zhang, He, et al. 2022). The ACC, a region central to evaluating outcomes and effort, also appears to be engaged: in animal models, VNS was found to enhance neural synchrony between the ACC and amygdala, coinciding with improved decision‐making performance (Cao et al. 2016). Overall, VNS can recalibrate functional networks in the limbic and frontal regions, aligning brain network dynamics in a way that favors adaptive reward processing.

In parallel, accumulating evidence suggests that VNS also influences core aspects of reinforcement learning, the process by which organisms learn from reward feedback. Both rodent and human studies report that VNS is associated with enhanced reward learning and adaptive behavioral adjustment (Oehrn et al. 2022). Mechanistically, this was accompanied by elevated brain BDNF in the mPFC and reversal of cocaine‐induced synaptic changes in the infralimbic cortex, indicating that VNS promotes neuroplasticity that reinforces goal‐directed learning over habitual drug reward memories (Driskill et al. 2024). Interestingly, VNS can bias decision‐making strategies in favor of more beneficial choices, as demonstrated in the rodent gambling task (Cao et al. 2016) and human behavior in the prisoner's dilemma task (Oehrn et al. 2022). These findings align with the notion that VNS primes the brain's reward circuit toward optimistic, goal‐directed choices, possibly by enhancing dopaminergic tone in individuals with initially lower DA activity.

#### 4.1.4 Pain and Nociception Management

VNS engages descending inhibitory pathways in the CNS that are fundamental to pain modulation. Afferent vagal signals relayed via the NTS can activate pain‐control centers in the brainstem, including the thalamus, hypothalamus, PBN, periaqueductal gray (PAG), and the LC (Shao et al. 2023). Through these connections, VNS taps into the endogenous analgesia network. Notably, stimulation of vagal afferents has been shown to drive descending serotonergic and noradrenergic pathways that project from the brainstem down to the spinal cord dorsal horn. Colloquially termed the descending antinociceptive systems (DAS), inputs to the PAG relay through the rostral ventromedial medulla (RVM), descend in the dorsal lateral funiculus, and project to interneurons of the dorsal horn (DH). While the serotonergic DAS largely utilizes the neurotransmitter 5‐HT, opioidergic and GABAergic mechanisms contribute at the supraspinal and segmental levels (Cui et al. 1999). The PAG, a well‐known opioidergic pain center, does not have direct projections to the DH, and instead relays its descending signals through the RVM (Behbehani and Fields 1979). A spinal relay for all descending non‐noradrenergic pain inhibition, the RVM is comprised of the nucleus raphe magnus and nuclei of the reticular formations (Newman 1985). Both the PAG and RVM receive ascending inputs from DH projection neurons, creating a feedback loop. The PAG and RVM are highly innervated by pain‐modulating centers, receiving projections from cortical and subcortical structures, thus contributing to conscious, stress, and emotional responses to pain (Dong et al. 1999; Hardy 1986; Helmstetter et al. 1998; Sakata et al. 1989). Collectively, these pathways release 5‐HT and NE in the spinal cord, which inhibit the transmission of nociceptive signals by hyperpolarizing the dorsal horn neurons and presynaptically suppressing incoming pain fiber activity (Dorr and Debonnel 2006; Shao et al. 2023).

In addition to direct neuronal inhibition of pain, VNS exerts anti‐inflammatory effects that can mitigate pain (see Section 4.2.1), especially in chronic conditions with an inflammatory component. Reducing systemic and local inflammation, VNS can directly alleviate pain caused by inflammatory processes. For example, in conditions like RA, inflammatory bowel disease (IBD), or neuropathic pain, where low levels of cytokines (TNF‐α at 5 pg/mL) sensitize nociceptors (Gold and Gebhart 2010). Indeed, clinical trials of VNS in disorders such as RA have demonstrated decreased cytokine levels and improvements in pain and joint swelling, linking vagal neuromodulation to tangible anti‐inflammatory benefits (Koopman et al. 2016). Similarly, studies in other chronic pain conditions have found that VNS can enhance pain‐control mechanisms, for example, by boosting Conditioned Pain Modulation (CPM), which is the body's intrinsic capacity to inhibit pain in one area when another area is stimulated (Pacheco‐Barrios et al. 2024). Thought to gauge DAS function and corresponding to diffuse noxious inhibitory control (DNIC), CPM is a phenomenon whereby a second noxious stimulus applied elsewhere decreases pain perception from the initial pain location (Le et al. 1992; Le Bars et al. 1979). Depending on the size of the electric field and stimulation strength, VNS‐induced DNIC and CPM may contribute to its antinociceptive effects. Additionally, the commonly deployed 10–50 Hz stimulation frequency blocks C‐fiber activity, which may also provide pain relief effects (Zhang, Chen, et al. 2024).

In summary, by recruiting brainstem analgesic circuits and attenuating inflammation, VNS modulates nociceptive processing at multiple levels of the neuroaxis, underlying its potential as an adjunct for chronic pain management.

### 4.2 Spleen/Immune System

#### 4.2.1 Cholinergic Anti‐Inflammatory Pathway

VNS activates a neural reflex that suppresses systemic inflammation via a spleen‐mediated circuit. Efferent vagal fibers originating in the DMV interface with sympathetic neurons in the celiac ganglion, which in turn project to the spleen. This constitutes an efferent cholinergic arm of the inflammatory reflex that can be activated in the brain through muscarinic acetylcholine receptor (mAChR)‐mediated mechanisms triggered by mAChR ligands and acetylcholinesterase (AChE) inhibitors (Pavlov and Tracey 2012). Central muscarinic cholinergic activation enhances efferent vagal cholinergic outflow. It facilitates downstream anti‐inflammatory signaling, with these central mAChRs serving as key regulators of the CAP without requiring peripheral muscarinic involvement (Pavlov et al. 2006). Following vagal stimulation, the splenic sympathetic nerve terminals release NE into the spleen. NE binds to β2‐adrenergic receptors on a specialized subset of splenic CD4+ T cells that express choline acetyltransferase (ChAT). The activation of these cholinergic T cells triggers their secretion of ACh, providing a non‐neuronal source of ACh in the spleen. ACh released from these T cells then binds to α7 nicotinic acetylcholine receptors (α7nAChR) on splenic macrophages. Activation of α7nAChRs on macrophages initiates an anti‐inflammatory intracellular cascade: it recruits and activates the JAK2/STAT3 pathway while inhibiting nuclear factor‐κB (NF‐κB) translocation and activity within these cells. In addition to the T cell‐dependent relay, NE release from the sympathetic nerve terminals directly binds to β‐adrenergic receptors on macrophages. This molecular cascade further inhibits the NF‐κB activation (Freire et al. 2022; Brinkman 2024). As a result, the production of proinflammatory cytokines (e.g., TNF‐α, IL‐1β, and IL‐6) is suppressed, anti‐inflammatory cytokines (e.g., Interleukin‐10 [IL‐10], Interleukin‐4 [IL‐4]) are upregulated, and macrophages undergo a phenotypic shift from proinflammatory M1 state toward anti‐inflammatory M2 state (Jin et al. 2023). In addition, VNS also exerts anti‐inflammatory effects beyond the spleen, notably influencing hepatic and enteric immune cells in the liver and intestine (see Section 4.7, Figure 6). VNS modulates local inflammatory responses through vagal efferents and enteric cholinergic neurons, activating α7nAChRs expressed on resident immune cells, including macrophages, dendritic cells, mast cells, and hepatic Kupffer cells (Wang, Zhan, et al. 2021). Taken together, this vagal anti‐inflammatory mechanism is often termed the “cholinergic anti‐inflammatory pathway” (CAP), and it serves as the efferent arm of the inflammatory reflex controlling immune responses (Pavlov et al. 2003; Murray and Reardon 2018) (Figure 4). The splenic and gastrointestinal components of CAP are sometimes described as distinct pathways, and whether or not they can be separably activated by precision neuromodulation technologies (including optogenetics, miniaturized electrical, and targeted ultrasound stimulators) remains an active area of study.

### 4.3 Endocrine System

#### 4.3.1 HPA Axis and Neuroimmune‐Endocrine Integration

The VN serves as a critical communication hub between the nervous and endocrine systems. Vagal afferents relay visceral information to central autonomic circuits that govern hormone release, while vagal efferents provide direct neural input to peripheral endocrine glands. VNS can thus modulate hormonal axis and help maintain endocrine homeostasis under stress and other challenges.

VNS profoundly influences the HPA axis, which controls stress hormone secretion. The NTS forms direct connections with CRH neurons in the PVN, modulating CRH secretion into the portal circulation. This vagally activated pathway stimulates adrenocorticotropic (ACTH) hormone release from the anterior pituitary, ultimately driving cortisol production in the adrenal cortex (Herman 2018; Camici et al. 2024) (Figure 4). This vagally mediated reflex is part of the body's adaptive response to stress and inflammation: cortisol elevations triggered via vagal afferents help terminate excessive inflammation and restore internal balance (Camici et al. 2024). For example, a recent study reported that taVNS significantly inhibited the salivary cortisol response under acute mental stress (Cuberos Paredes et al. 2025).

Notably, chronic VNS can also suppress an overactive HPA axis in clinical populations. In patients with refractory depression, chronic VNS therapy normalized an initially exaggerated pituitary–adrenal response. These patients showed abnormally high ACTH and cortisol responses to CRH challenge, but after 3 months of VNS, the CRH‐induced ACTH release and cortisol levels were reduced to healthy control concentrations (O'Keane et al. 2005). Similarly, in patients with epilepsy who had elevated baseline cortisol, long‐term VNS led to a significant reduction in serum cortisol toward normal levels (Majoie et al. 2010). These findings in humans indicate that VNS can reduce HPA axis hyperactivity, likely by re‐balancing hypothalamic CRH output and enhancing negative feedback control of cortisol secretion.

Taken together, VNS highlights the close neuroimmune–endocrine integration. The actions of vagal afferents and efferents illustrate a feedback loop: immune activation stimulates vagal afferents, which drive endocrine and neural suppression of inflammation. VNS can thus modulate cytokine levels and has been shown to rebalance immune mediators in humans alongside normalizing cortisol (Majoie et al. 2010; Silverman and Sternberg 2012). In summary, the current literature supports the concept that VNS stabilizes the internal hormonal milieu and prevents maladaptive overactivation of endocrine responses. Further work to explore cortisol release signaling via the enteric nervous system is now being explored by the Defense Advanced Research Projects Agency (DARPA) CoasterChase program; it is a fundamental research initiative focused on understanding and modulating the warfighter stress response through neurostimulation of the enteric nervous system, potentially enhancing VN signaling.

#### 4.3.2 Other Hormonal Interactions

Beyond the HPA axis, VN activity interacts with other neuroendocrine axes through autonomic reflexes and central information. One prominent example is the hypothalamic–pituitary–thyroid (HPT) axis. The thyroid gland receives parasympathetic innervation; vagal fiber densely innervates the thyroid blood vessels of the thyroid follicles. Experimental stimulation of the vagal nerve increases the thyroid blood flow and enhances hormone release in rats (Hotta et al. 2017). Conversely, interrupting vagal innervation impairs thyroid activity—for instance, surgical transection of the vagus/inferior laryngeal nerve leads to a drop in circulating thyroxine levels in rats (Fekete and Lechan 2014). Although direct human studies are limited, the principle of vagal modulation of the HPT axis suggests that VNS might influence metabolism and energy balance via effects on thyroid output. Another axis under vagal influence is the hypothalamic–pituitary–gonadal (HPG) axis. Evidence shows that vagal afferents convey information that the brain uses to regulate reproductive hormones. In animal models, disrupting vagal signaling has pronounced effects on gonadal function: bilateral vagotomy in female rats delays the onset of puberty and blunts ovarian steroid response (Linares et al. 2013). Thus, the VN can influence reproductive endocrine homeostasis in animals, though mechanistic human data are still emerging.

### 4.4 Cardiovascular System

#### 4.4.1 Heart Rate Control and Autonomic Balance

**FIGURE 5:**
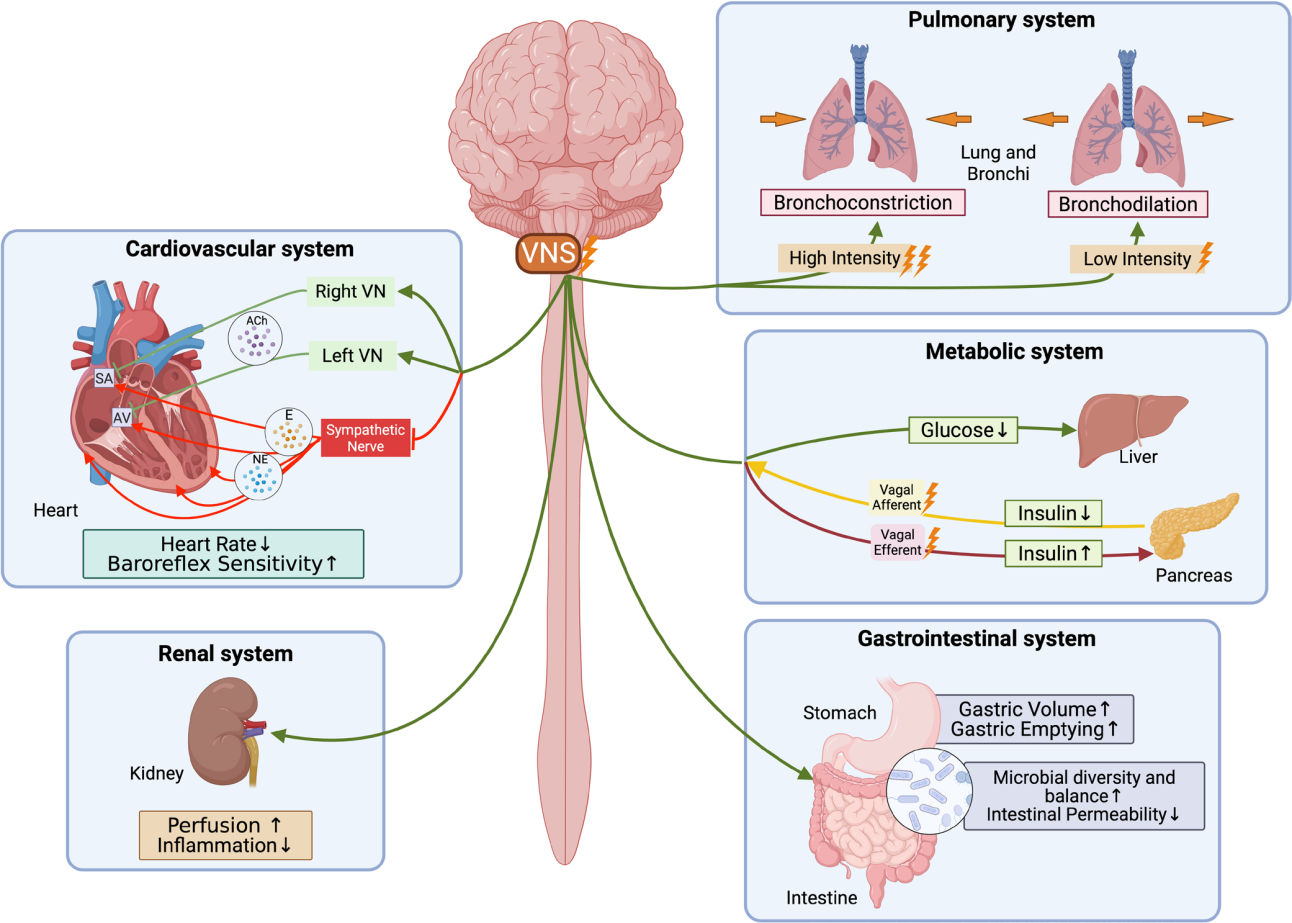
Mechanistic overview of the major effects of vagus nerve stimulation (VNS) on physiological systems. In the *cardiovascular system*, parasympathetic nerves release acetylcholine (ACh) onto the sinoatrial (SA) and atrioventricular (AV) nodes, resulting in decreased heart rate, while sympathetic nerves release norepinephrine (NE) and epinephrine (E), which increase heart rate and contractility. VNS promotes parasympathetic activity and suppresses sympathetic tone, thereby collectively reducing heart rate and enhancing baroreflex sensitivity, which promotes balanced autonomic regulation. In the *pulmonary system*, the intensity of VNS determines its effect: High‐intensity stimulation promotes bronchoconstriction, while low‐intensity stimulation supports bronchodilation. In the *metabolic system*, VNS reduces hepatic glucose production and differentially modulates insulin secretion, with afferent vagal activation suppressing insulin release and efferent vagal stimulation promoting insulin secretion. In the *gastrointestinal system*, VNS increases gastric receptive relaxation and enhances gastric emptying, while promoting microbial diversity and balance. In the *renal system*, VNS enhances renal perfusion and attenuates inflammation.

A well‐established effect of VN signaling is HR reduction due to ACh release at the sinoatrial (SA) node. The release of ACh counterbalances the sympathetic nervous system's accelerating effects (mediated by NE and epinephrine) on HR (Levy et al. 1993). Upon release, ACh binds to M2 muscarinic receptors expressed on pacemaker cells, subsequently activating the ACh‐dependent potassium current. This activation leads to membrane hyperpolarization. Concurrently, ACh modulates the hyperpolarization‐activated current. These dual mechanisms collectively contribute to the suppression of pacemaker automaticity (DiFrancesco et al. 1989). In addition, ACh can inhibit intracellular calcium transients via a cyclic adenosine monophosphate (CAMP)‐dependent signaling pathway, which also contributes to pacemaker slowing (Zhang and Mazgalev 2011; van Borren et al. 2010). Notably, the VN's influence is anatomically specialized: while both left and right branches innervate the SA and atrioventricular (AV) nodes, their distribution is asymmetrical. The right VN predominantly controls the SA node, the heart's primary pacemaker, while the left VN exerts stronger control over the AV node, which coordinates the timing between atrial and ventricular contractions (Ardell and Randall 1986). In contrast, the sympathetic nervous system elevates HR by releasing NE and epinephrine, which bind to beta‐adrenergic receptors on the SA node. This interaction increases the rate of depolarization, leading to faster heartbeats. Additionally, sympathetic activity enhances AV node conduction and myocardial contractility, collectively boosting cardiac output. VNS counters this by activating afferent fibers that engage the NTS in the brainstem, which inhibits sympathetic outflow via the rostral ventrolateral medulla and reduces NE release (Elamin et al. 2023); simultaneously, vagal efferents release ACh that dampens beta‐adrenergic signaling at the cellular level (Brack et al. 2013) (Figure 5). Furthermore, VNS can decrease adrenal catecholamine secretion, lowering circulating epinephrine and NE levels (Deuchars et al. 2018). Studies across multiple species, including rats, pigs, and humans, have demonstrated that VNS effectively decreases HR (Agarwal et al. 2016; Buschman et al. 2006; Yuan et al. 2017). The bradycardia effect, along with slowed AV conduction, underlines the use of VNS in controlling certain tachyarrhythmias (Capilupi et al. 2020). The net result of these actions is a decrease in cardiac output and oxygen demand, which can be beneficial in conditions of excessive cardiac workload. Importantly, the VNS intensity needs to be fine‐tuned to recruit B‐fibers to induce the bradycardia effect (Qing et al. 2018) with stimulation frequency optimized to ~30 Hz, as low‐intensity stimulation at 1 Hz is inefficient at reducing heart rate even when B‐fibers are activated (Ahmed et al. 2021).

Given its role in heart rate control, VNS is believed to exert a restorative influence on autonomic balance by increasing parasympathetic tone and concomitantly reducing excessive sympathetic activity (Deuchars et al. 2018). Electrical activation of vagal efferents directly enhances cardiac parasympathetic output, while afferent signaling to the NTS inhibits central sympathetic outflow (Olshansky et al. 2008). This shift toward parasympathetic dominance is quantified in heart rate variability (HRV) enhancement: VNS has been shown to significantly increase HRV indices, indicating elevated vagal modulation of HR (Machetanz et al. 2021). This effect on autonomic state balancing by VNS has been purported to stabilize cardiovascular regulation and has been associated with improved clinical profiles and outcomes in cardiovascular diseases (Jardine et al. 2005; Pahuja et al. 2023). Though it's generally accepted that large current preclinical VNS lowers HR, outcomes in human studies are heterogeneous. Some trials report substantial autonomic gains, while others show minimal or even negative effects. Table 1 summarizes its impact on HR and HRV in subjects with various health conditions. Multiple factors may contribute to the variable outcomes, including the VNS approach (iVNS vs. taVNS vs. tcVNS), laterality of stimulation (left vs. right), stimulation parameters (Machetanz et al. 2021; Atanackov et al. 2025), population characteristics (age, sex, disease state), treatment protocol (acute vs. chronic), and study design (pre vs. during vs. post, active vs. sham vs. control). Additionally, research has shown that the VNS effect on HRV is influenced by circadian rhythms and baseline physiological status, introducing further variables that require careful control in experimental stages (Geng, Yang, et al. 2022).

**TABLE 1:** Vagus nerve stimulation (VNS) effect on heart rate and various heart rate variabilities under health and different medical conditions in human studies.

Study authors	VNS approach, pulse width, frequency, duty cycle, amplitude	Sample size (sex, age)	Stimulation protocol	Study design	HR	SDNN	RMSSD	pNN50	LF	HF	LF/HF	Total power	SD1	SD2	Other
*Health*															
Clancy et al. (2014)	taVNS, 200 μs, 30 Hz, 10–50 mA	48 (24 F, 20–62 years)	Acute (Active/Sham, 15 min)	Active (During vs. Pre)	↓				NS	NS	↓	NS			MSNA: ↓
				Sham (During vs. Pre)	↓				NS	NS	NS	NS			
Antonino et al. (2017)	taVNS, 200 μs, 30 Hz, 10–50 mA	13 (13 M, 23 ± 1 years)	Acute (Active, 15 min)	Active (During vs. Pre)	↓				NS	NS	↓				
				Sham (During vs. Pre)	NS				↑	NS	NS				
De Couck et al. (2017)	taVNS, 250 μs, 25 Hz Study 1: mean 0.7 mA	Study 1: 30 (15 F: 23–58 years)	Study 1: Acute (Left/Right/Sham, within‐subject crossover, 10 min each, 5 min apart)	Right (During vs. Pre)		↑	NS		NS	NS	NS				
				Left (During vs. Pre)		NS	NS		NS	NS	NS				
				Sham (During vs. Pre)		NS	NS		NS	NS	NS				
	Study 2: mean: 1 mA	Study 2: 30 (15 F: 30–60 years)	Study 2: Subacute (1 h Right taVNS)	Start/Middle/End vs. Baseline		NS	NS		NS	NS	NS				
Bretherton et al. (2019)	taVNS, 200 μs, 30 Hz, 2–4 mA	Study 1: 14 (9 M, 69 ± 2 years)	Study 1: Acute, 15 min taVNS, 1 week break, 15 min Sham	Active vs. Sham											BRS: ↑
		Study 2: 48 (22 M, 65 ± 1 years)	Study 2: Acute, 15 min taVNS	During vs. Pre	↓	↑	↑	↑	↑	NS	NS	↑	↑	↑	BRS: ↑ ΔRR: ↑
				Post vs. Pre	↓	↑	NS	NS	↑	↑	NS	↑	NS	↑	
		Study 3: 26 (9 M, 64 ± 1 years)	Study 3: Chronic, V1 (15 min taVNS), daily for 2 weeks, V2 (15 min taVNS)	V2 vs. V1	NS	↑	↑	↑	NS	NS	NS	↑	↑	↑	Post ΔRR: ↑
				During vs. Pre	↓	↑			↑			↑		↑	
				Post vs. Pre	↓	↑			NS			↑		↑	ΔRR↑
Forte et al. (2022)	taVNS, 200–300 ms, 25 Hz, 30 s on–off. 1.2 ± 0.4 mA	28 (23 F, 23 ± 3 years)	Acute (Active or Sham, within‐subject crossover, 10 min each, 1 week apart)	Active (During vs. Pre)		↑	↑		↑	↑	NS				
				Sham (During vs. Pre)		NS	NS		↑	NS	↑				
				Active vs. Sham		↑	↑		NS	↑	↓				
Geng, Liu, et al. (2022)	taVNS, 250 μs, 20 Hz,	14 (8 M, 23 ± 1 years)	Acute (Active/Sham, within‐subject crossover, 5 min each, 3 days apart)	Active (During vs. Pre)		↑	↑	↑	NS	↑	NS				
				Sham (During vs. Pre)		NS	NS	NS	NS	↓	NS				
				Active vs. Sham		↑	↑	↑	NS	↑	NS	NS			
Geng, Yang, et al. (2022)	taVNS, 250 μs, 25 Hz, 5–35 mA	Study 1: 27 (13 M, 24 ± 1 years)	Study 1: Acute (Morning and Evening, 10 min each)	Morning (During vs. Pre)	↓	↑	↑	↑	NS	↑	↓	↑	↑	↑	
				Evening (During vs. Pre)	NS	NS	↑	NS	NS	↑	NS	NS	NS	NS	
				Morning vs. Evening	NS	NS	↑	↑	NS	↑	NS	NS	↑	NS	
		Study 2: 16 (8 M, 24 ± 1 years)	Study 2: Acute (Active, 4 × 5 min stim, 4 × 5 min post)	During 5 min/10 min vs. Pre			↑			↑	NS				
				During 15 min/20 min vs. Pre			NS			NS	NS				
Kaduk et al. (2025)	taVNS, 250 μs, 25 Hz, 30 s on–off	36 (18 F, 24 ± 3 years)	Acute (Active/Sham, Left/Right 30 min, different days)	Active (During vs. Pre)	↓	↑	↑			NS	↑				
				Active vs. Sham	NS	↓	↓			↓	NS				
				Left vs. Right	NS	NS	NS			NS	NS				
Gauthey et al. (2020)	taVNS, 200 μs, 5/20 Hz, mean 1.2–1.5 mA	28 (28 M, 27 ± 4 years)	Acute (5 Hz Active/25 Hz Active/Sham, within‐subject crossover, 10 min, continuous)	5 Hz vs. Sham	NS	↓	NS		↓	NS	↓				
				20 Hz vs. Sham	NS	↓	NS		↓	↓	NS				
				5 Hz (During vs. Pre)	NS	NS	NS		NS	NS	↑				
				20 Hz (During vs. Pre)	NS	NS	NS		NS	NS	NS				
Šinkovec et al. (2023)	taVNS, 2 ms, 20 Hz, 1 s on–off, 90/130 μA	50 (24 F, 20–39 years)	Acute (0/90/130 μA, within‐subject crossover, 20 min, 5 min apart)	0 μA vs. 90 μA vs. 130 μA	NS					NS					
Borges et al. (2019)	taVNS, 200–300 μs, 25 Hz, 30 s on–off	61 (16 F, mean 23 years)	Acute: 10 min each session, 5 min between sessions Study 1: Acute (0.5/1/1.5 mA)	During First 5 min vs. Pre			↑								
				0.5 mA vs. 1 mA vs. 1.5 mA			NS								
			Study 2: Set intensity 1 mA/Free Intensity (mean 1.78 mA)	During vs. Pre			↑								
				Set vs. Free			NS								
			Study 3: Active vs. Sham, Set vs. Free	During vs. Pre			↑								
				Active vs. Sham			NS								
				Set vs. Free			NS								
Brock et al. (2017)	tcVNS GammaCore	20 (13 F, 23–56 years)	Acute: 120 s Bilateral tcVNS	Post 90 min vs. Pre	↓										CVT: ↑↑
				Post 24 h vs. Pre											CVT: ↑
*Neurological disorders*															
Hirfanoglu et al. (2018)	iVNS, 500 ms, 30 Hz, 30 s on, 5 min off, 1.5 ± 0.5 mA	20 (9 F, 12 ± 4 years, 4–17 years)	Chronic Daily continuous, 12 months	Post 6 months vs. Pre	↓	↑	↑	↑	↑	↑	NS	↑			
				Post 12 months vs. Pre	NS	↑	NS	NS	NS	NS	NS	↑			
				Post 6 months vs. 12 month	NS	NS	NS	NS	↑	NS	NS	↑			
				VNS (Pre/Post 6 month/Post 12 month) vs. Control	↑	↓	↓	↓	↓	↓	NS	↓			
Jansen et al. (2011)	Left iVNS, 500 ms, 30 Hz, 30 s on, 5 min off 0.75–2.5 mA	17 (13 M, 3–16 years)	Chronic daily continuous, 3–36 months	Stage 2 sleep Post vs. Pre	NS	NS	NS	NS	NS	NS	NS	NS			
				Stage 2 sleep Pre vs. Control	↑	NS	NS	NS	NS	NS	SN	NS			
				Stage 2 sleep Post vs. Control	NS	NS	NS	NS	NS	NS	NS	NS			
				Slow wave sleep Post vs. Pre	NS	NS	NS	NS	↑	↓	↑	NS			
				Slow wave sleep Pre vs. Control	↑	NS	NS	NS	↑	↓	↑	NS			
				Slow wave sleep Post vs. Control	NS	NS	NS	NS	↑	↓	↑	NS			
Galli et al. (2003)	iVNS, 500 ms, 30 Hz, 30 s on, 5 min off, 1–1.75 mA	7 (4 M, 34–63 years)	Chronic daily continuous, 36 months	Daytime (Post 1 month vs. Pre)	NS				NS	NS	NS	NS			
				Nighttime (Post 1 month vs. Pre)	NS				NS	NS	NS	NS			
				Daytime (Post 36 months vs. Pre)	NS				NS	NS	NS	NS			
				Nighttime (Post 36 months vs. Pre)	NS				NS	↓	NS	NS			
Garamendi et al. (2017)	Left iVNS, 250 μs, 25 Hz, 1–2.5 mA	15 (13 M, 39 ± 8 years)	Chronic daily continuous, 10–15 months	Post 6 months vs. Pre	NS				NS	NS	NS				
				Post 12 months vs. Pre	NS				NS	NS	NS				
Kamath et al. (1992)	Left iVNS High stim: 500 ms, 30 Hz, 1 mA	4 (3 M, 21–47 years)	Chronic daily continuous, 2 weeks	High stim Post 2 weeks vs. Pre	NS				NS	↑	↓				
	Left iVNS Low stim: 130 ms, 2 Hz, 0.1 mA	4 (3 M, 21–47 years)		Los stim Post 2 weeks vs. Pre	NS				↑	NS	NS				
Ronkainen et al. (2006)	Left iVNS, 500 ms, 30 Hz, 30 s on, 5 min off, 0.25 mA increment to therapeutic level	14 (8 M, 34 ± 9 years)	Chronic daily continuous, 12 months	Pre vs. Control	↑	↓			↓	↓			↓	↓	VLF: ↓
				Post 12 months vs. Control	↑	↓			↓	↓			↓	↓	VLF: ↓
				Post vs. Pre	NS	NS			NS	NS			NS	NS	VLF: NS
Cadeddu et al. (2010)	iVNS	10 (6 M, 18–45 years)	Chronic daily continuous, 7.7 ± 2.3 months	Resting (Post 8 months vs. Pre)		NS	↑	↑	NS	NS	NS				
				Seizure (Post 8 months vs. Pre)					↓	↑	↓				
Schomer et al. (2014)	iVNS, patient‐specific parameter	9 (6 M, 29–36 years)	Chronic daily continuous, 1–14 weeks	Post vs. Pre					↓	NS	↓				
Verrier et al. (2016)	iVNS, 250 μs, 20 Hz, 30 s on, 5 min off, median 0.75 mA	28 (19 F, 19–66 years)	Chronic daily continuous, 2–4 weeks	Post vs. Pre	NS				NS	NS	NS				
Liu et al. (2018)	Left iVNS, 500 μs, 30 Hz, 30 s on, 5 min off, 0.8–2.6 mA	32 (21 M, 6–38 years)	Chronic daily continuous, 12 months	Post vs. Pre	NS	NS	NS	NS	NS	NS	NS	NS			MSE: ↑
				Pre vs. Control	NS	↓	↓	↓	↓	↓	NS	↓			MSE: ↓
Frei and Osorio (2001)	Left iVNS, 500 μs, 30 Hz, 30 s on, 5 min off, 1.25–3.25 mA	5 (4 M, 19–40 years)	Acute	ON vs. OFF	↓					↓					
Zaaimi et al. (2007)	iVNS, 500 μs, 30 Hz, 1.75 mA—2 mA	10 (4 M, 7–18 years)	Acute during sleep	ON vs. OFF	Mix										
Stemper et al. (2008)	iVNS, 500 μs, 30 Hz, 50 s on, 5 min off, 1.44 ± 0.4 mA	21 (11 F, 35 ± 12 years)	Acute	ON vs. OFF	NS				↑	↑					BP: NS
*Psychiatric disorders*															
Gurel et al. (2020)	tcVNS, GammaCore, 27 ± 7.5 V	25 (6 M, 19–70 years)	Acute: 120 s tcVNS	Active vs. Sham	↓				NS	NS	NS				SD1/SD2: ↑
Sperling et al. (2010)	Left iVNS, 500 μs, 15/30 Hz, 0.5–2.5 mA	9 (3 F, 44–65 years)	Acute	Pre vs. Control	↑		↓								
				ON vs. OFF	NS		NS								
*Cardiovascular diseases*															
De Ferrari et al. (2011)	Right iVNS, 2–10 s on, 6–30 s off 1.1–5.5 mA	32 (30 M, 56 ± 11 years)	Chronic daily continuous, up to 12 months	Post 3 months vs. Pre	↓	NS		↑							MSSD: NS
				Post 6 months vs. Pre	↓	NS		↑							MSSD: NS
				Post 12 months vs. Pre	↓			↑							
Premchand et al. (2014)	Left/Right iVNS, 250 μs, 10 Hz, 1.5–3 mA, 14 s on, 66 s off	60 (52 M, 51 ± 12 years)	Chronic daily continuous, 6 months	Post 6 months vs. Pre	↓	↑									
Kumar et al. (2023)	Right iVNS, 250 μs, 5 Hz, 2.4 ± 0.5 mA	52 (36 F, 57 ± 10 years)	Chronic daily continuous, 12 months	Post 12 months vs. Pre	NS	NS	NS	↓	↓	↓					
Nearing et al. (2021)	Left/Right iVNS, 250 μs, 10 Hz, 1.5–3 mA, 14 s on, 66 s off	25 (20 M, 47 ± 2 years)	Chronic daily continuous, 36 months	Post 6 months vs. Pre	↓	NS	NS		NS	NS	NS				
				Post 12 months vs. Pre	↓	NS	↑		NS	↑	NS				
				Post 24 months vs. Pre	↓	NS	↑		↑	↑	NS				
				Post 36 months vs. Pre	↓	↑	↑		↑	↑	NS				
Mbikyo et al. (2024)	taVNS, 20 Hz, 1 mA	21 (15 M, 31 ± 7 years)	Chronic: 1 h/day, 5 days/week, 12 weeks	Active vs. Sham (Post 1 month)	NS										SBP&DBP: ↓
				Active vs. Sham (Post 2 month)	NS										SBP&DBP: ↓
				Active vs. Sham (Post 3 month)	NS										SBP&DBP: ↓
Stavrakis et al. (2020)	taVNS, 200 μs, 20 Hz, 17 ± 13 mA	26 (12 M, 65 ± 15 years)	Chronic: 1 h/day, 6 months	Active vs. Sham	NS	NS	NS	NS	↑	↓	↑				
*Other diseases*															
Tarn et al. (2023)	tcVNS GammaCore	20 (18 F, 60 ± 14 years)	Chronic: twice daily, 54 days	Post vs. pre					NS	NS	NS				
Venborg et al. (2021)	tcVNS GammaCore	15 (13 F, 65 ± 10 years)	Subacute: 3 times/day for 4 days, once on day 5, Bilateral	Post 20 min vs. Pre	↓										CVT: ↑
				Post 24 h vs. pre	NS										CVT: NS
				Chronic: Post 5 days vs. Pre	NS										CVT: NS
Corrêa et al. (2022)	taVNS, 1 ms, 25–5 kHz	26 (16 M, 53 ± 17 years)	Chronic: 90 min/session, twice daily, 2 weeks	Active vs. Sham (Post)					NS	NS	NS				

*Note:* Comparisons are presented as A vs. B, where the arrow demonstrates the comparison of A with respect to B. ↓, A is significantly lower than B; ↑, A is significantly higher than B; NS, No significant difference between A and B. Sample size represents the number of subjects who received active VNS treatment, the Sham group refers to subjects who underwent sham VNS therapy, while the control group refers to matched healthy control participants. The stimulation parameter of transcutaneous cervical (tcVNS) by GammaCore may refer to Figure 1.

Abbreviations: BP, blood pressure; BRS, Baroreflex sensitivity; CVT, cardiac vagal tone; DBP, diastolic blood pressure; HF, high‐frequency power; HR, heart rate; LF, low‐frequency power; MSE, multiscale entropy; MSNA, muscle sympathetic activity; MSSD, mean squared successive differences; pNN50, percentage of successive RR intervals differing by > 50 ms; RMSSD, root mean square of successive differences; SBP, systolic blood pressure; SDNN, standard deviation of normal‐to‐normal intervals; Total Power, total spectral power; V1, visit 1; V2, visit 2; VLF, very low frequency.

Despite the heterogeneity in outcomes, VNS commonly demonstrates a reduction in HR and improvement in HRV when using within‐subject comparison, specifically during‐ versus. pre‐stimulation measurements in acute protocols (Antonino et al. 2017; Bretherton et al. 2019; Forte et al. 2022; Geng, Liu, et al. 2022; Geng, Yang, et al. 2022; Kaduk et al. 2025; Borges et al. 2019; Frei and Osorio 2001), and post‐ vs. pre‐treatment in chronic protocols (Bretherton et al. 2019; Hirfanoglu et al. 2018; Premchand et al. 2014; Nearing et al. 2021; De Ferrari and Schwartz 2011). However, between‐group comparisons (active vs. sham or control) often fail to demonstrate these same benefits (Kaduk et al. 2025; Gauthey et al. 2020; Hirfanoglu et al. 2018; Ronkainen et al. 2006; Liu et al. 2018; Sperling et al. 2010). This discrepancy may occur because within‐subject comparisons tend to control for individual baseline differences and confounding factors by using each participant as their own control, potentially making VNS‐induced changes easier to detect. In contrast, between‐group comparisons must account for inter‐individual variability and may be influenced by factors such as the placebo effect (Kim 2020). Therefore, while VNS appears to produce measurable physiological changes within individuals, establishing therapeutic efficacy through controlled trials remains challenging and requires careful consideration of study design factors that may influence the detection of treatment effects.

In parallel, VNS has reflexive effects on blood pressure regulation. NTS is the central relay for baroreceptor input, and VNS enhances baroreflex sensitivity and can reset the baroreflex set‐point to a lower pressure (Saku et al. 2014). NTS activation by VNS leads to inhibition of the rostral ventrolateral medulla (RVLM). This central reflex simultaneously reduces sympathetic outflow and enhances parasympathetic activity, effectively increasing baroreflex sensitivity (Saku et al. 2014; Agarwal et al. 1990). The immediate outcome is vasodilation and a subsequent reduction in blood pressure (Antonino et al. 2017; Mbikyo et al. 2024; Capilupi et al. 2020).

#### 4.4.2 Cardioprotective Effects

VNS engages several protective pathways that benefit the heart at the cellular and molecular levels. One key mechanism is the activation of the CAP pathway. Through this neuroimmune reflex, VNS significantly reduces circulating inflammatory mediators and suppresses macrophage activation (Elamin et al. 2023; Arya et al. 2023). In both acute (e.g., myocardial infarction [MI]) and chronic (e.g., heart failure) cardiac injury models, these anti‐inflammatory effects translate into less tissue damage (Zhao et al. 2013; Bazoukis et al. 2023). VNS also mitigated oxidative stress in the myocardium. By modulating cardiac redox signaling and promoting nitric oxide (NO), VNS limits the accumulation of reactive oxygen species (ROS) and associated oxidative injury. This includes upregulating antioxidant defenses and improving endothelial function via NO, which together reduce ischemia‐reperfusion (I/R) damage (Bezerra et al. 2017). VNS also mitigates cardiomyocyte pyroptosis by suppressing NLRP3 inflammasome activation and its downstream effectors, as evidenced by reduced myocardial levels of NLRP3, cleaved caspase‐1, and gasdermin D (the pyroptotic pore‐forming fragment), and a corresponding decrease in IL‐1β release (Lu et al. 2023). By blunting this cell death pathway, VNS attenuates myocardial damage in ischemic heart injury and heart failure models, indicating that anti‐pyroptotic effects (via inflammasome inhibition) are an important component of its cardioprotective mechanism (Singh et al. 2025). Additionally, VNS has been shown to trigger anti‐apoptotic pathways in cardiac cells, reducing cell death during stress conditions (Prathumsap et al. 2023; Xuan et al. 2017). Collectively, these actions: (1) anti‐inflammatory, (2) anti‐oxidative, (3) anti‐pyroptotic, and (4) anti‐apoptotic form a multi‐faceted cardioprotective profile for VNS.

### 4.5 Respiratory System

#### 4.5.1 Bronchomotor and Airway Reflexes

Vagal efferents provide innervation to the airways, controlling bronchial smooth muscle, whereas afferent fibers innervate the respiratory tract and mediate protective reflexes that optimize breathing. Efferently, the VN regulates bronchial smooth muscle primarily through parasympathetic cholinergic pathways, where ACh release causes bronchoconstriction via muscarinic receptors (Fryer and Jacoby 1998). Additionally, vagal non‐adrenergic, non‐cholinergic pathways mediate bronchodilation via neurotransmitters such as vasoactive intestinal peptide and NO (Lammers et al. 1992). In contrast, afferent pathways are involved in airway protective reflexes, including the cough reflex and the Hering‐Breuer inflation reflex. The vagal afferent fibers detect airway irritants and lung inflation, triggering protective reflexes like cough to expel harmful substances and the Hering‐Breuer reflex to prevent lung over‐inflation by halting inspiration (Mazzone and Undem 2016; Polverino et al. 2012). These brainstem‐mediated responses coordinate respiratory defenses and regulate breathing patterns through sensory feedback from nociceptive fibers and pulmonary stretch receptors.

The VNS effect on the bronchomotor reflex could be either bronchoconstriction or bronchodilation, depending on the stimulation intensity. In a swine study, high‐intensity or direct vagal stimulation will predominantly drive efferent fibers, releasing ACh onto airway M3 receptors and causing bronchoconstriction (Hoffmann et al. 2012; Buels and Fryer 2012). High‐intensity stimulation above the C‐fiber activation threshold also contributes to apnea side effects (Chang et al. 2020). In contrast, low‐level VNS can achieve the opposite effect via the afferent‐mediated reflex, which may involve NE release and interaction with β2‐adrenergic receptor, relaxing airway smooth muscle. Subthreshold stimulation that primarily excites vagal afferents has been found to inhibit histamine‐induced bronchoconstriction (Hoffmann et al. 2012; Yuan and Silberstein 2016b) (Figure 5). In practical terms, VNS may evoke an autonomic reflex arc, whereby stimulation leads to reflex sympathetic outflow and adrenaline release, which in turn relaxes bronchial smooth muscle.

VNS may also modulate the cough reflex; recent trials in healthy adults showed that altering stimulation frequency and site can change cough reflex sensitivity and potentially improve chronic refractory cough (Ng et al. 2024; Michalowski et al. 2021). On the other hand, experimental evidence suggests that VNS can also modulate respiratory pathways in a therapeutic manner. For example, preliminary studies in animals have explored whether vagal stimulation might ameliorate bronchospasm in conditions like asthma by activating the “anti‐inflammatory” or bronchodilatory reflex circuits (Wu et al. 2024).

#### 4.5.2 Interaction With the Brainstem Respiratory Center

Given the strong influence of vagal sensory input on the respiratory central pattern generator, it is not surprising that VNS can alter respiratory timing and rhythm. Physiological studies showed that VNS could reset the respiratory cycle—for instance, vagal bursts delivered during inspiration tend to prematurely terminate that inspiration (mimicking a stretch receptor signal via the Hering‐Breuer reflex), whereas stimulation during expiration can prolong the expiratory pause (Mazzone and Undem 2016). Further, recent work demonstrates VN action potentials uniformly synchronize with the respiratory cycle in porcine models (Vallone et al. 2021; Sevcencu et al. 2018) and in one recent human microelectrode study (Ottaviani et al. 2020), although synchronized respiratory and cardiac activity may reflect the recording modality deployed (Verma et al. 2022) and recording needle position within somatotopically organized vagal fibers. Recent human observations indicate that VNS disrupts respiratory rhythm control in the NTS, resulting in an increased respiratory rate but reduced respiratory amplitude, tidal volume, and oxygen saturation. Concurrently, stimulation of the recurrent laryngeal nerve branch causes vocal cord adduction, narrowing the upper airway, and triggering obstructive apneas (Parhizgar et al. 2011). There is also a possibility of central adaptation: with repeated or chronic VNS, the brainstem might adjust to the continuous afferent input, potentially raising the threshold for response over time (Vadhan and Tadi 2019). In a case of piglets, strong vagal stimulation may even further activate the superior laryngeal nerve (a branch of the Vagus) and induce apnea and laryngospasm, an extreme illustration of vagal arrest of respiratory rhythms (Lawson 1981). Fortunately, the levels of clinical VNS are titrated to avoid prolonged apnea, but mild breath‐holding or irregular breathing can occur when the vagus is stimulated at higher intensities.

Under normal conditions, inspiration is accompanied by a reflex suppression of vagal cardiac efferent activity (leading to a slight HR increase) and expiration by a restoration of vagal tone (HR decrease); this phenomenon is known as respiratory sinus arrhythmia (RSA). VNS has been shown to disrupt RSA: the normal coupling between respiratory and cardiac cycles was blunted, and RSA magnitude (the difference between inspiratory and expiratory HRs) significantly decreased during vagal stimulation. Essentially, the vagal input imposed by VNS interfered with the timing that normally links heart and lung rhythms; it is an effect the authors suggested could reduce the efficiency of oxygen delivery (Zaaimi et al. 2009). This example underscores that while VNS increases vagal activity, it may also perturb the finely tuned patterns of cardiorespiratory coordination. On the other hand, there is interest in utilizing VNS in controlled ways to improve autonomic‐respiratory integration in certain conditions. Napadow et al. propose timing VNS bursts to specific phases of the respiratory cycle (known as respiratory‐gated VNS) to augment baroreflex sensitivity or treat hyperventilation by using the vagal input to reinforce beneficial reflex timing instead of random disruption (Sclocco et al. 2017). In summary, respiratory modulation by VNS is done by augmenting vagal afferent input to the brainstem respiratory network, engaging the body's natural inspiratory‐inhibitory reflex, and thereby fine‐tuning the timing of the respiratory cycle.

### 4.6 Metabolic System

#### 4.6.1 Mitochondrial Function

VNS has been shown to upregulate pathways that drive mitochondrial biogenesis. Parasympathetic signaling via ACh to AMP‐activated protein kinase (AMPK) through muscarinic M3 receptors and CaMKKβ: this in turn promotes the coactivator PGC‐1α—a master regulator of mitochondrial DNA replication and biogenesis (Liu, Wu, et al. 2024). VNS also engages SIRT1, a NAD^+^‐dependent deacetylase that co‐activates PGC‐1α and other transcription factors. For example, in a rodent study, VNS elevated SIRT1 levels and decreased acetylation of FOXO1 and p53 (targets of SIRT1) in skeletal muscle, consistent with enhanced mitochondrial biogenic signaling (Xin et al. 2022).

By improving mitochondrial content and enzyme expression, VNS can enhance oxidative phosphorylation efficiency and ATP generation. Preclinical studies in heart tissue have shown that VNS or transcutaneous auricular stimulation reverses heart failure‐induced downregulation of electron transport chain genes, leading to improved ATP synthesis (Chakraborty et al. 2024). In a rat model of myocardial infarction, vagal activation helps restore the expression of key metabolic proteins (e.g., upregulating GLUT4 glucose transporters and CPT1α for fatty acid oxidation), thereby providing mitochondria with substrates to optimize ATP production (Liu, Wu, et al. 2024; Luo et al. 2020).

VNS also exerts a powerful regulatory effect on mitochondrial ROS production and antioxidant defenses. Parasympathetic stimulation through ACh dampens excessive mitochondrial ROS generation and helps stabilize mitochondrial membrane potential. In myocardial ischemia studies, VNS reduced oxidative stress damage, partly by activating the Nrf2/HO‐1 pathway, a key cellular antioxidant response, which upregulates enzymes that detoxify ROS and protect cells against oxidative stress and apoptosis (Liu, Wu, et al. 2024; Zhang, Zhang, et al. 2024).

VNS also significantly influences mitochondrial dynamics, a crucial process maintaining mitochondrial health through balanced mitochondrial fission and fusion (Xue et al. 2017). Experimental evidence demonstrates that VNS mitigates stress‐induced mitochondrial fragmentation by modulating key proteins involved in these processes. Specifically, in a rat myocardial ischemia model subjected to β‐adrenergic stress, excessive mitochondrial fission was observed, characterized by upregulation of the fission mediators dynamin‐related protein 1 (Drp1) and mitochondrial fission 1 protein (Fis1), alongside downregulation of the fusion‐promoting proteins mitofusins (Mfn1/Mfn2) and optic atrophy protein 1 (OPA1) (Xue et al. 2017). VNS effectively reversed these molecular alterations, normalizing mitochondrial dynamics and restoring a healthier, interconnected mitochondrial network with elongated rather than fragmented mitochondria. Mechanistically, this benefit is attributed to cholinergic activation of the muscarinic M3 receptor, triggering AMPK signaling. VNS‐induced AMPK activation tilts the balance toward fusion via suppression of Drp1 and induction of mitofusins (Xue et al. 2017).

#### 4.6.2 Glucose Homeostasis

The VN provides a key link between the brain, pancreas, and liver in regulating blood glucose. Vagal efferents innervating the pancreas stimulate insulin release from β‐cells, especially during the initial phase of digestion (Teff 2008). Electrical vagus stimulation on the efferent arm of the VN raises glucagon levels alongside insulin in pre‐clinical models (Ahrén and Taborsky Jr 1986). Such modulation supports glucose balance and may benefit conditions like diabetes by enhancing insulin sensitivity and reducing glycemic variability (Liu, Wu, et al. 2024). However, stimulation of vagal afferents inhibits insulin secretion, suggesting these opposing efferent and afferent vagal effects may be considered, especially when developing VNS as a potential therapeutic approach for metabolic disorders (Meyers et al. 2016) (Figure 5).

Beyond its pancreatic effects, vagal signaling directly modulates hepatic glucose production and uptake. ACh released from vagal efferents binds hepatocyte muscarinic receptors, triggering intracellular pathways that promote glycogen storage and suppress gluconeogenesis (Liu, Wu, et al. 2024). Notably, vagal stimulation regulates liver enzymes involved in gluconeogenesis and glycogenolysis, such as inhibiting phosphoenolpyruvate carboxykinase (PEPCK). This process reduces hepatic glucose output and improves insulin sensitivity in the liver (Liu, Wu, et al. 2024). Hepatic vagal efferents releasing ACh also exert a permissive effect on insulin action, increasing hepatocellular glucokinase activity and thereby potentiating glucose uptake (Hampson and Agius 2007) (Figure 5).

Mechanistic evidence from VN and VNS studies underscores its role in glycemic control. Silverman and colleagues first showed that food intake in a pre‐clinical model increased endogenous VN firing from 5 up to 50 spikes/s when measured with cervical VN cuff recordings (Silverman et al. 2018). In rodents, acute electrical stimulation of the subdiaphragmatic vagus significantly lowers blood glucose levels without increasing plasma insulin (Hoornenborg et al. 2023). Chronic VNS can drive even lasting metabolic improvements. In a study on diet‐induced obese pigs, 12 weeks of continuous bilateral VNS reversed insulin resistance; the treated animals' whole‐body insulin sensitivity and hepatic glucose uptake matched those of lean controls (Malbert et al. 2017). These findings further suggest that the integral vagal pathway is critical in and may directly modulate glycemic control in pathological states by both augmenting peripheral glucose disposal and suppressing hepatic glucose production, an important aspect of postprandial glucose homeostasis.

As an example of new VNS technologies providing unique opportunities for targeting organ‐specific pathways and achieving unique functional outcomes, ultrasound neuromodulation of sensory neurons within the liver was recently shown to improve glucose metabolism in diabetic models. It was shown that the effect was mediated via liver‐hypothalamic pathways that, when activated, modulate glucose uptake in peripheral organs (without directly affecting insulin secretion) (Cotero et al. 2022). A subsequent pilot clinical study demonstrated safety and preliminary results in patients with Type 2 Diabetes (Ashe et al. 2023), providing initial evidence that new targeted approaches to VNS may enable researchers to continue to optimize stimulation parameters and engage organ and tissue‐specific functions and therapeutic outcomes.

In addition, FUS targeting the hepatic system also shows promising results in preclinical models. Cotero et al. demonstrated in mice, rats, and swine that peripheral FUS targeted to the hepatoportal nerve plexus can noninvasively activate vagal and spinal afferent‐dependent liver‐brain neural pathways. Daily peripheral FUS treatment improved glucose tolerance, insulin sensitivity, and peripheral glucose uptake, while multi‐omic profiling revealed FUS‐induced intestinal transcriptomic changes, including enhanced nutrient transport and mitochondrial function (Cotero et al. 2022).

#### 4.6.3 Lipid Metabolism and Energy Balance

The autonomic nervous system orchestrates hepatic lipid metabolism by regulating the balance between lipid synthesis, storage, and breakdown. VNS has been shown to alter the expression of hepatic metabolic regulator genes such as SREBP‐1c, PPARα, and SIRT1, key regulators in energy and lipid metabolism (Liu, Wu, et al. 2024; Manca et al. 2024), and by modulating such regulators, vagal input may limit excessive lipid accumulation in hepatocytes. Indeed, an intact VN appears necessary for certain anti‐steatotic reflexes: leptin's ability to reduce liver fat depends on vagal signaling to stimulate triglyceride export from the liver, further highlighting the vagal role in lipid metabolism (Metz et al. 2022). We speculate that chronic alcohol use and/or alcohol abuse leading to increased gut permeability, causative of increased circulating lipopolysaccharide (LPS) resulting in disrupted vagal afferent signaling, thereby disrupting healthy regulation of lipid metabolism genes such as SREBP‐1c, PPARα, and SIRT1 (Liu, Wu, et al. 2024; Manca et al. 2024). Two hits via direct alcohol upregulation SREBP‐1c and vagal dysfunctional disinhibition may collectively contribute to liver lipid accumulation and potentially liver failure in overabuse of alcohol.

**FIGURE 6:**
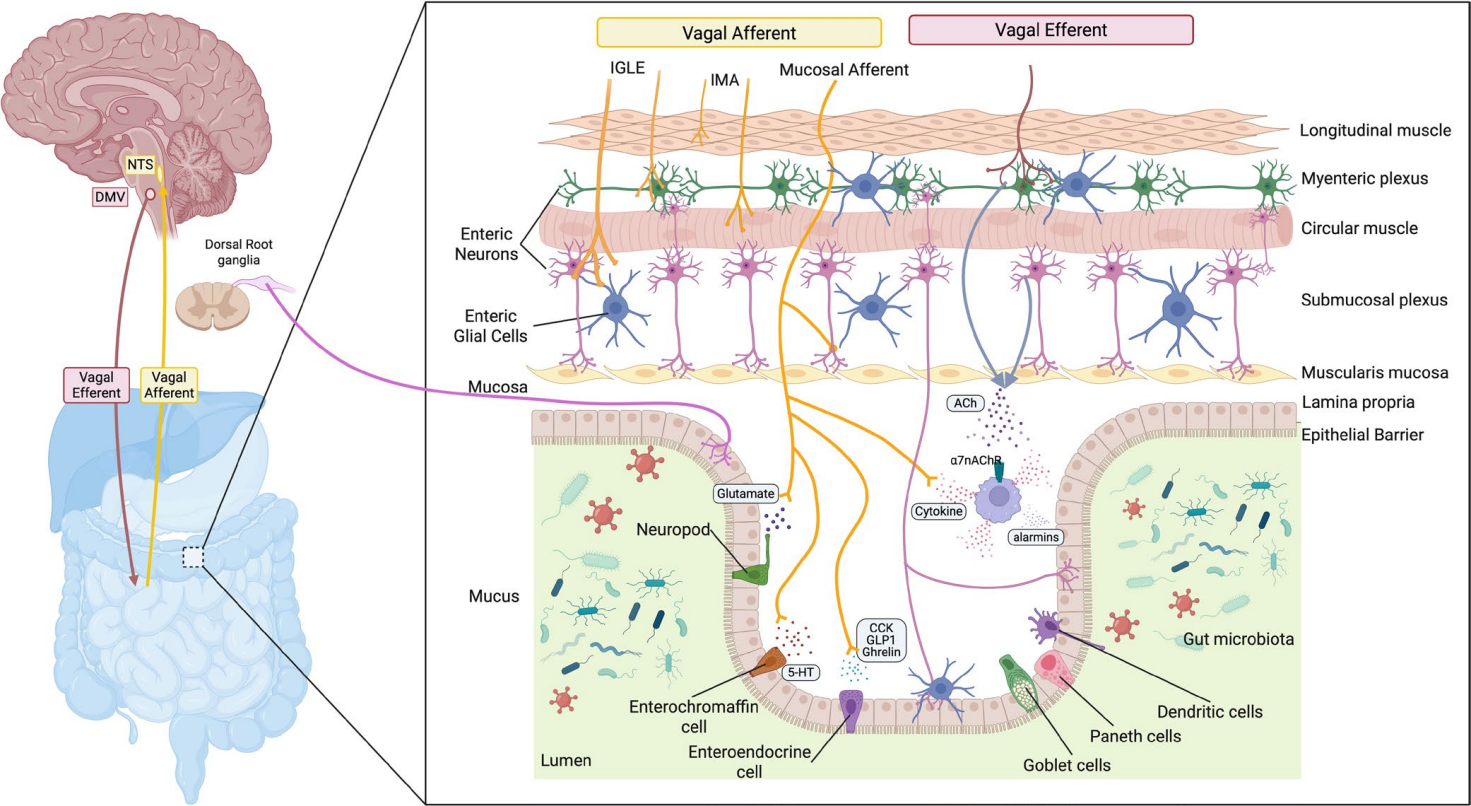
Brain‐gut axis: vagal network in gastrointestinal function and inflammatory control. Vagal afferent fibers (yellow path) transmit mechanical and chemical stimuli from the gut to the nucleus tractus solitarius (NTS) in the brainstem, originating from terminals that include intraganglionic laminar endings (IGLE), intramuscular arrays (IMA), and mucosal afferents. These afferents interact with enteroendocrine cells, enterochromaffin cells, and neuropods, which release neurotransmitters like glutamate, serotonin (5‐HT), and gut hormones in response to luminal stimuli. The gastrointestinal tract also sends sensory afferents to the spinal cord via the dorsal root ganglia (pink path). Vagal efferent fibers (red path), terminating in the myenteric and submucosal plexi, modulate gastrointestinal motility, secretion, and immune function. Epithelial injury can activate the macrophage to release cytokines, which in turn activate the vagal afferent pathway. In response, vagal efferent modulates enteric neurons to release ACh, which binds to α7 nicotinic acetylcholine receptors (α7nAChRs) on immune cells, glia, and epithelial cells, suppressing proinflammatory cytokine release and supporting intestinal immune homeostasis. Enteric glial cells (EGCs) support this network by modulating barrier integrity and inflammatory responses. DMV, dorsal motor nucleus of the vagus; NTS, nucleus tractus solitarius.

VN activity also interfaces with central circuits that govern appetite and energy expenditure, thereby impacting body weight and metabolic homeostasis. A functional gut‐brain axis curtails meal size via satiety signals; for instance, the cholecystokinin (CCK) released during meals activates vagal afferents and triggers satiation by NTS and hypothalamic responses (Owyang and Heldsinger 2011). VNS can potentiate these pathways: vagal stimulation has been observed to suppress food intake by enhancing feelings of fullness and reducing appetite. Studies report that VNS tends to lower ghrelin (hunger hormone) and elevate leptin and peptide levels, indicating a shift toward satiety signals (Gil et al. 2011; Loper et al. 2021) (Figure 6). This neuromodulation of gut‐brain communication can correct dysregulation seen in obesity. Complementing its effect on appetite, VNS can also increase energy consumption by activating brown adipose tissue (BAT). In clinical studies of epilepsy patients with implanted VNS devices, activating stimulation significantly increased resting metabolic rate and altered BAT glucose uptake, and while average BAT activity did not rise dramatically, those with the greatest stimulation‐driven increase in energy expenditure exhibited the largest jump in BAT activity, suggesting a causal relationship (Vijgen et al. 2013). Engaging hypothalamic‐brainstem circuits, VNS influences the full spectrum of energy homeostasis: it curbs appetite through augmented satiety signaling and boosts energy expenditure via metabolic activation.

### 4.7 Gastrointestinal System

#### 4.7.1 Swallowing, Brain‐Gut Axis and Vagovagal Reflexes

Swallowing consists of three sequential phases: oral, pharyngeal (oropharyngeal), and esophageal. While the oral phase is voluntary, the pharyngeal and esophageal phases are involuntary. VN plays a critical role in the coordination between the voluntary and involuntary phases of swallowing. Following mastication and bolus preparation, the bolus is propelled from the oral cavity into the pharynx, where it stimulates mechanoreceptors in the oropharynx whose afferents travel predominantly in the pharyngeal branch of the VN to the NTS to initiate the involuntary phase of swallowing through the VN. In humans, anterior‐posterior tonsillar pillars and the posterior pharyngeal wall are the most sensitive areas for eliciting the swallow reflex. Afferents for the reflex travel to the brainstem by several cranial nerves, that is, the maxillary branch of the trigeminal nerve, glossopharyngeal, and the superior laryngeal branch of the VN (Pommerenke 1928). The VN supplies the superior, middle and inferior pharyngeal constrictors and hence is the principal motor nerve for the pharyngeal phase of swallowing. It is notable that the nerve to muscle fiber ratio, 1:2 to 1:6 for the pharyngeal constrictors is in direct contrast 1:2000 for gastrocnemius muscle and it is even higher than the extraocular muscles (Feinstein et al. 1955). The above allows extremely fine coordination of these muscles that is needed especially for a function like aero‐protective reflex that prevents aspiration into tracheobronchial tree and lungs during swallowing. The superior laryngeal branch of the VN supplies the larynx. Receptors for the aero‐protective reflex are present in the oropharynx as well as in the esophagus, and afferents travel to the brainstem via the superior laryngeal and other branches of the VN (Shaker 2006). Electrical stimulation of the superior laryngeal nerve in animals elicits swallow reflex that is comprised of the pharyngeal and esophageal phases of swallow (Jean 2001). Afferents in the pharyngeal and superior laryngeal branches of the VN travel to the NTS, and a swallow pattern generator located in its vicinity (in the brain stem) coordinates the esophageal phase of swallowing. The efferent nerves fire sequentially to elicit peristalsis in the proximal skeletal and distal smooth muscles of the esophagus and the relaxation of the lower esophageal sphincter. These efferent impulses consist of two distinct types: initial inhibitory that elicit relaxation, followed by excitatory that mediate contraction phases of peristalsis (Gidda and Goyal 1984). Stimulation of a transected peripheral end of cervical VN induces esophageal contractions at the onset and end of stimulation and relaxation of the lower esophageal sphincter during the period of relaxation (Gidda and Goyal 1983; Goyal and Rattan 1975). Cooling of the VN in animal studies blocks swallow‐mediated peristalsis in the esophagus and lower esophageal sphincter relaxation (Reynolds et al. 1985). Gastric distension of the stomach elicits relaxation of the lower esophageal sphincter (LES) and crural diaphragm (transient lower esophageal sphincter relaxation) that is important in belching and vomiting; it is also an important mechanism of gastroesophageal reflux in patients with reflux disease. In animal studies, cooling of the VN prevents transient LES relaxation (Martin et al. 1986). It is thought that vagal efferent nerves synapse with the inhibitory and excitatory motor neurons of the myenteric plexus to elicit the relaxation and contraction phases of esophageal peristalsis (Goyal and Chaudhury 2008). The VN also carries two types of afferent fibers from the esophageal wall: low‐threshold mechanoreceptors that modulate peristaltic reflexes, and high‐threshold nociceptors that transmit pain signals responsible for heartburn and esophageal pain (Sengupta 2006). Recent reviews of VNS for swallowing dysfunction show promising results for oropharyngeal dysphagia across several studies (Florie et al. 2021). However, the evidence remains inconclusive, and randomized, double‐blind, sham‐controlled clinical trials with an adequate sample size are needed.

Moving beyond esophageal innervation, the VN extends its influence to gastric function through the anterior and posterior vagal trunks. The anterior gastric branch of the VN, derived from the left vagus, primarily innervates the anterior aspect of the stomach, including the lesser curvature and pyloric region. It plays a key role in regulating pyloric sphincter tone, coordinating gastric emptying, and contributing to pancreaticobiliary reflexes via its hepatic branch. In contrast, the posterior gastric branch, originating from the right vagus, innervates the posterior fundus and body of the stomach and sends fibers to the celiac plexus, modulating broader visceral and enteropancreatic reflexes. Both branches participate in mediating gastric accommodation, peristalsis, and gastric acid secretion, integrating sensory and motor control over stomach functions (Moore and Dalley 2018).

Vagal afferent neurons innervate the gut from the esophagus to the proximal colon, where they detect a wide range of physiological stimuli, including mechanical signals (e.g., stretch or distension) via the intraganglionic laminar endings (IGLE), and intramuscular arrays (IMA), as well as chemical signals (e.g., nutrients, pH, and gut hormones such as CCK, GLP‐1, ghrelin) through receptors on the mucosa endings (Han et al. 2022; Chuyue et al. 2020; Waise et al. 2018) (Figure 6). All these afferent signals converge and are processed centrally at the NTS. The NTS neurons project to hypothalamic nuclei and limbic structures that govern feeding and autonomic responses, of which circuitry links vagal input to central appetite‐regulating pathways (Rinaman 2010). Such vagal afferent satiety signaling is critical for meal termination and energy homeostasis, and VNS has been shown to amplify these signals—reducing food intake and promoting weight loss in both animal and human studies (Loper et al. 2021; De Lartigue 2016). In parallel, vagal afferent input is relayed via brainstem parabrachial and thalamic pathways to the insular and cingulate cortices, contributing to the interoceptive awareness of GI sensations (Bonaz et al. 2019). Vagal afferent processing in the CNS triggers reflex DMV motor activity.

Vagal efferent fibers descend and directly innervate enteric ganglia in the myenteric plexus (Figure 6), synapsing on enteric neurons that express 5‐HT, vasoactive intestinal polypeptide (VIP), enkephalin (ENK), galanin (GAL), tyrosine hydroxylase (TH), and nitric oxide synthase (NOS) (Kirchgessner and Gershon 1989; Cailotto et al. 2014). Thus, by stimulating the enteric nervous system, vagal efferents can regulate smooth muscle contractility, secretory function, and local immune modulation (Han et al. 2022). Notably, vagal efferent fibers have not been observed in the submucosal plexus, suggesting that vagal modulation of secretomotor responses involves an intermediate synapse in the myenteric plexus. Communication via vagal afferents and efferents underlines the bi‐directional “brain‐gut axis,” enabling CNS regulation of the digestive process (Powley 2021).

The integration of vagal afferent and efferent pathways forms the basis of vagovagal reflexes, which represent fundamental regulatory mechanisms that coordinate GI functions. Key vagovagal reflexes include the receptive relaxation reflex, where esophageal distension during swallowing triggers coordinated lower esophageal sphincter relaxation and proximal gastric fundus relaxation to accommodate incoming food (Travagli et al. 2003); the gastric accommodation reflex, in which gastric distension detected by mechanoreceptors triggers reflexive gastric fundus relaxation to accommodate the meal intake and acid secretion (Vanden Berghe et al. 2009); the gastro‐pancreatic reflex, where gastric acid and nutrients sensing in the duodenum stimulate pancreatic enzyme and bicarbonate secretion (Schwartz et al. 1979). These neural circuits adjust digestive timing, relaxing stomach walls during meals while tightening intestinal entry points based on nutrient detection to balance food processing with absorption needs. In the small intestine, although the enteric nervous system generates basic motor patterns, vagal efferent modulates intestinal peristaltic velocity and rhythm, promoting coordinated segmental contractions for optimal nutrient mixing and transit (Yan et al. 2024). In animal studies, acute vagal afferent supramaximal stimulation can expand gastric volume and impair antral contractility through direct relaxation of gastric smooth muscle, resulting in immediate dilation of the corpus and antral regions of the stomach (Lu et al. 2020). Such expansions persist long‐term with chronic vagal stimulation, further promoting VNS's role in gastric accommodation and nutrient absorption (Dai et al. 2020) (Figure 5). In parallel, vagal efferents are equally pivotal in regulating digestive secretions. Cholinergic vagal fibers innervate the stomach's secretory apparatus, stimulating acid production during digestion. This is evidenced by the fact that vagotomy or vagal blockade (atropine) markedly blunts meal‐induced gastric secretion, whereas vagal stimulation can evoke copious acid production (Sharkey et al. 1991; Sarna and Daniel 1975). Vagal efferents additionally modulate biliary secretion and likely influence intestinal fluid secretion and absorption via enteric reflex pathways (Sharkey and Mawe 2023). In the pancreas, vagal efferents drive exocrine secretion (Mussa and Verberne 2008), and experimental studies have shown that VNS can elicit robust pancreatic secretion of digestive enzymes in rats (Wisdom et al. 1993). Therapeutic VNS harnesses these same cholinergic pathways to modulate gastrointestinal function. By increasing vagal efferent drive, VNS may enhance gastric emptying, promote peristalsis, and improve digestive efficiency, offering potential benefits for conditions such as gastroparesis and constipation (Liu, Lv, et al. 2022).

While conventional VNS enhances vagal signaling to improve digestive function, vBloc therapy takes the opposite approach by employing high‐frequency electrical pulses to create a conduction block of vagal fibers at the gastroesophageal junction. The system delivers intermittent blocking cycles that temporarily interrupt both afferent and efferent vagal transmission, which disrupts key vagovagal reflexes, including gastric accommodation and gastric emptying reflexes (Apovian et al. 2017). This blocking pattern reduces gastric fundus relaxation capacity, leading to reduced meal accommodation and enhanced satiety signaling to the NTS (Mehta 2015; Johannessen et al. 2017). Additionally, vBloc alters gastric motility by disrupting vagal efferent control of gastric smooth muscle, resulting in delayed gastric emptying and prolonged gastric distension signal (Mehta 2015). However, the gut hormones were found unchanged following vBloc therapy in both short‐ and long‐term studies (Johannessen et al. 2017).

#### 4.7.2 Microbiota and Gastrointestinal Inflammation

Beyond its neural and endocrine roles, the VN is a key regulator of intestinal immune homeostasis and gut barrier integrity. Vagal afferents sense inflammatory cytokines, bacterial metabolites, microbial products, and peptides released from activated enteroendocrine cells to central autonomic networks that modulate local immunity and intestinal permeability (Han et al. 2022; Bonaz et al. 2018) (Figure 6). Notably, it has been recently demonstrated that gut inflammatory signals draining into the portal venous system are sensed by hepatic vagal afferents, which relay gut inflammatory signals to the brainstem to trigger vagal motor reflexes that promote immune tolerance (Teratani et al. 2020).

VN's influence on the gut ecosystem extends to shaping the composition of the microbiota and overall mucosal homeostasis. Immune‐regulatory vagal signaling promotes a tolerant environment for beneficial microbes while helping to counteract inflammation‐driven microbial dysbiosis. VNS can beneficially alter the gut microbiome, increasing microbial abundance and diversity (Yan et al. 2024; Liu, Dai, et al. 2024) (Figure 5). VNS has been shown to strengthen the intestinal barrier, thereby reducing the leakage of toxins and bacteria into the tissue (Bonaz et al. 2016). Recent evidence demonstrates that VNS preserves both BBB and intestinal barrier integrity following ischemic stroke by stabilizing mast cells and preventing the release of barrier‐disrupting proteases such as chymase, which would otherwise degrade tight junction proteins, including ZO‐1, Occludin, and Claudin‐5 (Wang et al. 2024). Notably, the anti‐inflammatory role of VNS helps to prevent excessive inflammatory responses to commensal microbes or dietary antigens (Browning et al. 2017).

The vagal anti‐inflammatory reflex in the gut appears to be “hard‐wired” and can function independently of the spleen and CD4 T cells (Matteoli et al. 2014). Rather, vagal efferents target cholinergic myenteric neurons in close contact with muscularis resident macrophages expressing α7nAChR, constituting a direct neuroimmune link to modulate inflammation at the source (Cailotto et al. 2014). However, VNS likely exerts anti‐inflammatory effects in the gut in at least three ways: (a) activating vagal efferent pathways leading to enteric nervous system release of ACh, and a variety of neurotransmitters with immune‐modulatory function (5‐HT, VIP, neuromedin U, NOS) (Populin et al. 2021); (b) stimulating transmitter release from the peripheral ends of afferent neurons, such as tachykinins and calcitonin gene‐related peptide (Holzer 1988); and (c) stimulating afferent neurons that signal to the brain to activate efferent pathways, including CNS sympathetic outputs (Willemze et al. 2018) that activate inhibitory adrenergic β2 receptors on gut macrophages (Matheis et al. 2020) and most gut immune cells (Cervi et al. 2014).

In addition to cholinergic gut macrophage inhibition, VNS may also involve enteric glial cells (EGC) as intermediaries. EGCs express α7nAChR (Langness et al. 2017), and secrete s‐nitrosoglutathione (GSNO), which strengthens the intestinal barrier and reduces inflammation (Savidge et al. 2007). One study showed that chemical depletion of EGCs can diminish the protective effects of VNS in a model of gut I/R (Langness et al. 2017). Additionally, beyond its role in appetite regulation, CCK (particularly CCK‐8) functions as an anti‐inflammatory mediator by reducing neutrophil infiltration, inhibiting NO overproduction, and suppressing NF‐κB‐mediated inflammatory signaling (Meng et al. 2002). In dogs, intestinal cells release predominantly CCK‐58 in response to fatty acid, while VN terminals store CCK‐58 and CCK‐8 but selectively release only CCK‐8 during electrical vagal stimulation, mediating local anti‐inflammatory effects (Chang et al. 2000).

VNS studies in humans have shown promising reductions in GI inflammation and improvements in conditions marked by immune dysregulation (see Section 5 below) (Bonaz et al. 2019). In summary, VNS engages vagovagal and intrinsic GI neuroimmune reflexes that reinforce the intestinal barrier, suppress excessive inflammation, and foster a balanced microbiome, which are key elements of GI physiological homeostasis.

### 4.8 Renal System

#### 4.8.1 Renal Blood Flow

The VN has demonstrated important effects on renal blood flow. A recent mouse study by Cheng et al. provides robust anatomical evidence for parasympathetic nerve fibers innervating the main renal artery, its segmental branches, and the renal pelvis, suggesting the presence of a brain‐kidney axis mediated through the VN (Cheng et al. 2022). Functionally, VNS may enhance blood flow by inhibiting sympathetic overactivity and renin‐angiotensin‐aldosterone system (RAAS) activation. These mechanisms may collectively promote renal perfusion and mitigate cardiac‐renal interaction in heart failure patients (Wu, Liao, et al. 2023). VNS has been shown to reduce renin and angiotensin II levels in animal models, suggesting direct regulatory effects on renal hemodynamics (Wu, Liao, et al. 2023). Clinically, acute taVNS led to a significant reduction in serum aldosterone levels in healthy subjects, though the result did not differ significantly from sham treatment. This suggests that short‐term VNS may exert limited or context‐dependent (age, sex) effects on renal hormonal output in non‐pathological settings, and the mechanism requires further investigation (Veiz et al. 2023).

#### 4.8.2 Renoprotective Mechanisms

Beyond hemodynamics, VNS engages neuroimmune pathways that protect the kidney from injury. The immunomodulation effect of splenic CAP appears to extend to the kidneys (Nakamura and Inoue 2020). In the preclinical model of acute kidney injury (AKI), VNS markedly dampens renal inflammatory responses. For example, vagal stimulation prior to the kidney I/R injury blunted the typical surge in circulating cytokines and attenuated AKI (Inoue et al. 2016). Similarly, targeted VNS also reduced renal injury and inflammation in mice with systemic lupus erythematosus (Shimoura et al. 2023).

In addition to immunomodulation, VNS downregulates renal sympathetic output, which further contributes to renoprotection. Excessive sympathetic activation is known to worsen kidney injury, causing renal vasoconstriction, ischemia, and activation of fibrotic pathways (Dibona and Kopp 1997). By restoring vagal tone, VNS inhibits this harmful sympathetic drive. The resulting improvements in renal perfusion and decreased oxidative stress can limit acute ischemic damage and slow chronic injury progression (Figure 5). Recent mechanistic studies in mice reveal that VNS activates two distinct neuroimmune circuits converging on the spleen to mediate renoprotection: the classical CAP via vagal efferent, and a novel vagosympathetic reflex where vagal afferents activate C1 neurons that modulate splenic nerve activity to the generation of α7nAChR‐positive splenocytes, including macrophages, T and B Cells (Tanaka et al. 2021; Inoue et al. 2016). Both pathways require spleen‐dependent mechanisms, and VNS provides kidney protection even when systemic inflammation remains unchanged (Tanaka et al. 2021). In summary, VNS exerts renoprotective effects through a dual mechanism of damping neurogenic stress (sympathetic and RAAS activity) and suppressing inflammation via the CAP and activating specialized neuroimmune circuits through splenic immune cells (Tanaka et al. 2021; Inoue et al. 2016; Hilderman and Bruchfeld 2020).

## 5 Clinical Applications

Table 2 summarizes mechanistic evidence of VNS effects on medical conditions discussed in this section.

**TABLE 2:** Preclinical and clinical evidence of vagus nerve stimulation (VNS) effects on medical conditions through different mechanisms.

	Efficacy evidence	Inefficacy evidence	Brain circuit modulation	Neural plasticity and synaptic remodeling	Autonomic balance	Anti‐inflammatory pathways	Cardio‐protection	GI regulation	Endocrine system regulation	Metabolic regulation
*Neurological disorders*										
Epilepsy	Lim et al. (2022)^d^ Abbasi et al. (2021)^d^ Melese et al. (2024)^d^ Kong et al. (2024)^d^ Ben‐Menachem et al. (1994)^a^ Handforth et al. (1998)^a^	Bauer et al. (2016)^a^ ^,^ ^b^	Clifford et al. (2024)^d^	Ben‐Menachem et al. (1995) Coa (2022)	Genç et al. (2024)^b^	Majoie et al. (2010)			Majoie et al. (2010)	
Stroke rehabilitation and motor recovery	Abdullahi et al. (2023)^d^ Jiang et al. (2020)^d^ Du et al. (2024)^d^ Dawson et al. (2021)^a^ Dawson et al. (2023)^a^		Fan et al. (2025)^d^	Meyers et al. (2018)^c^	Wang et al. (2025)^a^	Du et al. (2024)^d^ Zhao et al. (2019)^c^				
Traumatic brain injury	Neren et al. (2016)^d^ Zhang, Li, et al. (2022)^d^ Hakon et al. (2020)		Neren et al. (2016)^d^	Pruitt et al. (2016)^a^ Smith et al. (2005)^a^		Tang et al. (2020)^a^		Bansal et al. (2012)^a^		
Disorder of consciousness	Briand et al. (2020)^d^ Dong et al. (2023)^d^ Zhou et al. (2023)^a^ Noé et al. (2020)	Yifei et al. (2022)	Yu, Yang, et al. (2017) Dong and Feng (2018)^a^	Yu et al. (2021)	Osińska et al. (2022)					
Alzheimer's and Parkinson disease	Vargas‐Caballero et al. (2022)^d^ Shan et al. (2025)^d^ Evancho et al. (2024)^d^ Broncel (2024)^a^ Zhang et al. (2023)^a^	Lench et al. (2023)^a^	Murphy et al. (2023)^a^ Marano et al. (2024)^a^ Zhang et al. (2023)^a^ Farrand et al. (2017)^a^	Kaczmarczyk et al. (2018)^c^ Mondal et al. (2024)^a^		Cai et al. (2019)^a^ Yu et al. (2023)^a^ Mondal et al. (2024)^a^		Kaut et al. (2019)	Merrill et al. (2006)	
Tinnitus	Yakunina and Nam (2021)^d^ Tyler et al. (2017)^a^	Kreuzer et al. (2014)^b^	Yakunina and Nam (2021)^d^	Engineer et al. (2013)^a^						
Migraine, cluster headache and chronic pain	Song et al. (2023)^d^ Reuter et al. (2019)^d^ Costa et al. (2024)^d^ Duff et al. (2024)^d^ Shao et al. (2023)^d^ Shi et al. (2021)^a^ Gaul et al. (2016)^a^ Tassorelli et al. (2018)^a^ Diener et al. (2019)^a^ Najib et al. (2022)^a^		Zhang et al. (2021)^a^	Cornelison et al. (2020)^a^ Shi et al. (2021)^a^	—	Boström et al. (2019) Shi et al. (2021)^a^				
*Psychiatric disorders*										
Depression	Bottomley et al. (2020)^d^ Aaronson et al. (2017)^b^ Conway et al. (2025)^a^	Rush et al. (2005)^a^	Fang et al. (2016)^b^ Nahas et al. (2007) Conway et al. (2013) Armitage et al. (2003)	Carpenter et al. (2004)^a^ Furmaga et al. (2011)^a^ Conway et al. (2012)	Sperling et al. (2010)	Lespérance et al. (2024)			O'Keane et al. (2005)	
Anxiety disorder and PTSD	Srinivasan et al. (2024)^a^ Bremner, Gurel, Jiao, et al. (2020)^a^	Grolaux (2019)	Wittbrodt et al. (2020)^a^ Li et al. (2020)^b^	Shin et al. (2019)^a^	Gurel et al. (2020)^a^	Bremner, Gurel, Jiao, et al. (2020)^a^			—	
Attention‐deficient/hyperactivity disorder	Zhu et al. (2022)^d^ Yildiz et al. (2024)^a^		—	—						
Autism spectrum disorder	Zhu et al. (2022)^d^ Levy et al. (2010)^b^		—							
Substance use disorder	Ward et al. (2020)^d^ Gazi et al. (2022)^a^ Wang, Xu, et al. (2021)^a^			Wang, Xu, et al. (2021)^a^		Yue et al. (2023)^a^				
Eating disorder	Melis et al. (2020)									
Obsessive‐compulsive disorder	Zhu et al. (2022)^d^ George et al. (2008)									
*Inflammatory and autoimmune diseases*										
Sepsis	Wu, Zhang, et al. (2023)^a^ Kohoutova et al. (2019)^a^	Kox et al. (2015)^b^			Castel et al. (2023)^c^ Kohoutova et al. (2019)^a^	Wu, Zhang, et al. (2023)^a^ Borovikova et al. (2000)^a^			Kohoutova et al. (2019)^a^ Borovikova et al. (2000)^a^	Kohoutova et al. (2019)^a^
Arthritis	Peterson et al. (2024)^a^ Brock et al. (2021)^b^	Baker et al. (2023)^a^			Jensen et al. (2022)^b^	Koopman et al. (2016) Marsal et al. (2021) Brock et al. (2021)^b^			Tang et al. (2018)	
Inflammatory bowel disease	Sahn et al. (2023)^a^ Pikov (2023)^d^				Sahn et al. (2023)^a^	D'Haens et al. (2023) Sinniger et al. (2020)		Meroni et al. (2021)^a^	Youssef et al. (2024)^a^	
Systemic lupus erythematosus	Aranow et al. (2021)^a^				Jensen et al. (2022)^b^	Aranow et al. (2021)^a^			Aranow et al. (2021)^a^	
Systemic sclerosis	Bellocchi et al. (2023)^a^				Bellocchi et al. (2023)^a^	Bellocchi et al. (2023)^a^				
Primary Sjögren's syndrome	Tarn et al. (2023)^a^		Tarn et al. (2023)^a^		Tarn et al. (2023)^a^	Tarn et al. (2019)				
*Cardiovascular diseases*										
Atrial fibrillation	Stavrakis et al. (2020)^a^ Stavrakis et al. (2017)^a^				Stavrakis et al. (2020)^a^	Stavrakis et al. (2017)^a^ Stavrakis et al. (2020)^a^	Stavrakis et al. (2020)^a^ Shen et al. (2011)^c^			
Myocardial infarction and reperfusion injury	Kruchinova et al. (2025)^a^ Yu, Huang, et al. (2017)^a^				Chen, Zhou, et al. (2016)^a^ Lu et al. (2023)^a^	Yu, Huang, et al. (2017)^a^ Calvillo et al. (2011)^a^ Lu et al. (2023)^a^	Shinlapawittayatorn et al. (2013)^a^ Katare et al. (2009)^a^ Nuntaphum et al. (2018)^a^ Chen, Zhou, et al. (2016)^a^ Lu et al. (2023)^a^			Nuntaphum et al. (2018)^a^ Katare et al. (2009)^a^ Shinlapawittayatorn et al. (2014)^a^
Heart failure	Premchand et al. (2014)^b^ Stavrakis et al. (2022)^a^	Zannad et al. (2015)^a^ Gold et al. (2016)^a^			Kumar et al. (2023)^b^ Zhang et al. (2009)^a^	Stavrakis et al. (2022)^a^	Zhang et al. (2009)^a^		Zhang et al. (2009)^a^	Chakraborty et al. (2024)^a^
Hypertension	Mbikyo et al. (2024)^a^				Annoni et al. (2019)^a^					
*Respiratory diseases*										
Asthma	Miner et al. (2012) Steyn et al. (2013)					Sévoz‐Couche et al. (2024)^a^				
Acute respiratory distress syndrome	Taha et al. (2024)^d^ Tornero et al. (2022)^a^ Corrêa et al. (2022)^a^ Li et al. (2021)^a^				Corrêa et al. (2022)^a^	Tornero et al. (2022)^a^ Corrêa et al. (2022)^a^ Li et al. (2021)^a^			Corrêa et al. (2022)^a^	
*Gastrointestinal and metabolic disorders*										
Obesity	Fadel et al. (2023)^d^ Ikramuddin et al. (2014)^a^ Apovian et al. (2017)^a^ Sarr et al. (2012)^a^	Hua et al. (2024)^d^	Gil et al. (2011)^a^ Biraben et al. (2008)^c^		Dai et al. (2020)^a^			Matyja et al. (2004)^c^ Dai et al. (2020)^a^	Gil et al. (2011)^a^ Apovian et al. (2017)^a^ Dai et al. (2020)^a^	
Diabetes	Yin et al. (2019)^a^ Li et al. (2014)^a^	Kufaishi et al. (2025)^a^				Okdahl et al. (2024)^a^			Li et al. (2014)^a^ Malbert et al. (2017)^a^	
Gastrointestinal disorder	Veldman et al. (2025)^d^ Shi et al. (2024)^a^ Wu et al. (2021)^a^ Zhu et al. (2021)^a^		Hou et al. (2023)^a^		Hou et al. (2023)^a^ Zhu et al. (2021)^a^	Hou et al. (2023)^a^		Zhu et al. (2021)^a^ Li et al. (2025)^a^ Hou et al. (2023)^a^	Hou et al. (2023)^a^	
*Renal disease*										
Acute kidney injury	Inoue et al. (2016)^a^ Deng et al. (2022)^a^ Wang et al. (2020)^a^					Inoue et al. (2016)^a^ Deng et al. (2022)^a^ Wang et al. (2020)^a^				Deng et al. (2022)^a^ Wang et al. (2020)^a^
*Other disease*										
Long COVID	Khan et al. (2024)^d^ Pfoser‐Poschacher et al. (2025)^b^ Zheng et al. (2024)			—	Pfoser‐Poschacher et al. (2025)^a^	Verbanck et al. (2021)			Pfoser‐Poschacher et al. (2025)^b^	

*Note:* This table summarizes key mechanistic pathways on the target medical condition only. The table features representative studies selected for mechanistic demonstration. The presented evidence serves as an illustrative synthesis rather than an exhaustive catalog of the VNS literature. —: There is a theoretical mechanism but limited evidence on target medical populations.

^a^ Randomized controlled trial.

^b^ More than or equal to 30 participants.

^c^ Animal study.

^d^ Systematic review or meta‐analysis.

### 5.1 Neurological Disorders

#### 5.1.1 Epilepsy

The Treatment of epilepsy by VNS involves a complex neuroanatomical pathway that begins with direct stimulation of the NTS via afferent VN. The NTS serves as the primary hub, projecting to other brainstem nuclei, including LC and PBN (Hachem et al. 2018). In VNS‐treated patients, direct evidence of LC has been observed in the form of increased P3 event‐related potential amplitude during stimulation, a proxy for heightened NE release in the brain (Carron et al. 2023). The LC is connected to the DRN, from which noradrenergic and serotonergic neurons project with widespread effects on cerebral networks and are postulated to be key to the VNS response. The PN is closely connected to the NTS, LC, and DRN, and has long‐ranging projections to the hypothalamus, thalamus, and amygdala. These regions then connect to the ACC, PFC, and insula, which are all implicated in VNS response (Clifford et al. 2024; Henry et al. 1998; Ko et al. 1996; Liu et al. 2003; Zhu et al. 2020; Narayanan et al. 2002) (Table 3). The neurochemical change and brain network activation are considered fundamental to VNS's antiseizure properties. VNS triggers the widespread release of neuromodulators that tilt the balance toward inhibition. Notably, VNS increases central GABA while reducing excitatory amino acids (glutamate/aspartate) levels, consistent with enhanced inhibitory tone (Ben‐Menachem et al. 1995). Elevated NE and 5‐HT induced by VNS are also implicated in seizure suppression, as these transmitters can dampen hyperexcitable cortical circuits and raise the seizure threshold (Berger et al. 2021). Over time, repeated VNS may induce more enduring structural and functional changes that counter epileptogenesis and strengthen seizure resistance. Brain plasticity enhancement and synaptic remodeling have been noted in limbic circuits with chronic VNS (Coa 2022); for instance, VNS in epileptic animal models promotes neurogenesis in the hippocampal dentate gyrus and other molecular alterations that could reorganize the hyperexcitable network (Sen et al. 1998). In humans, functional neuroimaging provides evidence of lasting network reorganization under VNS. PET blood‐flow studies show that VNS acutely activates a distributed set of deep and cortical regions, with increased perfusion in the thalamus, hypothalamus, and insular cortex, and relative reduction in limbic regions like the amygdala and hippocampus, and some of these changes persist after months of therapy (Carron et al. 2023; Henry et al. 1998; Sen et al. 1998). Evidence from neurophysiological measurements, including Electroencephalography (EEG) and Magnetoencephalography (MEG), shows that VNS consistently modulates cortical network dynamics, with effects varying by frequency band, brain state, and clinical responsiveness (Table 4). During acute stimulation, VNS typically induces cortically desynchronization, reflected by reduced Phase Lag Index (PLI), particularly in clinical responders (Sangare et al. 2020; Bodin et al. 2015; Vespa et al. 2021; Qin et al. 2024). This desynchronization is accompanied by increased integration in slow wave (delta and theta) connectivity, coupled with reduced integration in fast wave (alpha and beta) connectivity, implying a transient reset of pathological hypersynchronized network activity known to potentiate epileptiform foci (Lanzone et al. 2022; Li et al. 2023). VNS‐mediated desynchronization is most prominent in responders during non‐rapid eye movement (NREM) sleep, accompanied by decreased network integration metrics such as global efficiency (Vespa et al. 2021). Over months to years of therapy, VNS leads to gradual remodeling of cortical networks. Studies report a sustained decline in interictal epileptiform discharges, prolonged spike‐free intervals, and reduced paroxysm frequency, indicating durable suppression of epileptogenic activity (Wang et al. 2009; Koo 2001). With chronic use, VNS also induces progressive desynchronization of brain network activity and promotes increased functional segregation (Babajani‐Feremi et al. 2018; Fraschini et al. 2013; Marrosu et al. 2005; Coa et al. 2022) (Table 4). In animal models, chronic VNS increases seizure thresholds, as quantified by amygdala electrode current to evoke a seizure, preventing the progressive lowering of threshold seen in untreated epileptic rats (Alexander and McNamara 2012). These findings suggest that chronic VNS consolidates the acute desynchronizing effects into a stabilized, more balanced, and efficient network topology, which may underline its sustained seizure‐reducing and neuromodulatory effects (Carron et al. 2023; Ding et al. 2023). Additionally, EEG studies show that VNS induces long‐lasting neuroplastic changes by modulating neurotrophin expression, stimulating hippocampal LTP, increasing synaptic activity, promoting neurogenesis, and potentially inhibiting apoptosis, particularly in pathological conditions (Coa 2022). VNS also engages homeostatic inflammatory pathways: in patients who respond to VNS, peripheral inflammatory cytokines shift favorably (e.g., proinflammatory IL‐6 level falls, and anti‐inflammatory IL‐10 rises), a change hypothesized to create a more seizure‐resistant milieu (Majoie et al. 2010). However, VNS effect on HRV is complex, as evidenced by the intricate relationship between VNS parameters and different HRV measures (Table 1) (Genç et al. 2024).

**TABLE 3:** Invasive vagus nerve stimulation (iVNS) effects on epilepsy and depression from functional imaging.

	Epilepsy								Depression				
Study/neuroimaging	Henry et al. (1998) PET				Ko et al. (1996) PET	Liu et al. (2003) BOLD fMRI	Zhu et al. (2020) BOLD fMRI	Narayanan et al. (2002) BOLD fMRI	Mu et al. (2004) BOLD fMRI	Bohning et al. (2001) BOLD fMRI	Conway et al. (2006) PET	Zobel et al. (2005) SPECT	Nahas et al. (2007) BOLD fMRI
Stimulation parameter	High: 500 μs, 30 Hz, 30 s on, 5 min off Low: 130 μs, 1 Hz, 30 s on 180 min off				30 Hz, 2 mA	250 μs, 30 Hz, 30 s on, 66 s off	250 μs, 30 Hz, 30 s on, 5 min off	250 μs, 30 Hz, 30 s on, 30 s off	130/250/500 μs, 20 Hz, 13.6 s on, 41 s off	500 μs, 20 Hz, 13 s on, 103 s off	500 μs, 20 Hz, 30 s on, 5 min off	500 μs, 20 Hz, 30 s on, 5 min off	Not disclosed for treatment
Sample size (sex, age)	10 (6 M, 23–51 years)				3 (3 M 25–54 years)	5 (4 M, mean: 32 years)	15 (12 M, 19 ± 12 years)	5 (2 M, 6–20 years)	9 (7 M, 27–60 years)	9 (3 M, 35–58 years)	4 (4 F, age not disclosed)	12 (6 M, 31 ± 11 years)	9 (6 F, 47 ± 6 years)
Study design	Acute: 20 h		Chronic: 3 months		Acute during	Acute during	Chronic: 3 months	Acute during	Acute during	Acute during	Chronic: 3 weeks	Chronic: 4 weeks	Chronic: up to 20 months
	High	Low	High	Low									
Medulla	↑B								↓L				
Cerebellum	↑B	↑B	↑B	↑B	↑L Inf.	↑B	↑B Sup.		↓R Inf. Med.		↑L		↓L
Thalamus	↑B	↑B	↑B	↑B	↑R	↑B		↑B				↓R	
Hypothalamus	↑B	↑B	↑B	↑B						↑L			
Hippocampus	↓B	↓B							↓L			↓L	
Amygdala	↓B	↓B								↑L		↓B	
Cingulate gyrus	↓B Post.	↓B Post.				↑B	↑ B Cingulum Mid. ↓ L Ant.		↓R Ant.		↑B Ant.	↓R Post.	
Basal ganglia								↑L					
Insula	↑B	↑B				↑B	↓L	↑B	↑L				↓R
Orbitofrontal	↑B	NS							↑R Ant.	↑B	↑B		
Prefrontal									↑L		↑	↑L	↓
Frontal	↑B Inf.	↑R Inf.					↓L		↓R Inf. Med.		↑R Sup. Med.	↓L Med.	↓R Med. L
Temporal	↑R				↑R Post.	↑ Med.		↑R	↑L Mid. ↓R. Sup.	↑L	↓B	↓B	↓R
Parietal	↑B Inf.	↓B Inf.	↑B Inf.	↑B Inf.		↑B			↑R Sup.	↑B	↓R		↓B
Occipital						↑B	↑ L Cuneus			↑B			↓L
Postcentral gyrus	↑R	↑R	↑R	↑R				↑L	↓R				
Caudate									↑L			↓	
Putamen					↑L				↑L				

Abbreviations: Ant., anterior; B, bilateral; BOLD fMRI, blood oxygenation level dependent functional magnetic resonance imaging; Inf., inferior; L, left; Med., medial; PET, positron emission tomography; Post., posterior; R, right; SPECT, single photon emission computed tomography; Sup., superior.

**TABLE 4:** Invasive vagus nerve stimulation (iVNS) effects on epilepsy and depression from neurophysiological measurements.

Authors	Measurement approach	Demographic (responsiveness, sex, age)	Treatment length	Study design	Metrics	Key findings
*Epilepsy*						
Sangare et al. (2020)	EEG	35 subjects (20 R: 12 M, 42.8 ± 3.3 years; 15 NR: 8 M, 47.3 ± 3.0 years)	VNS treated for a median of 7 years	Acute ON vs. OFF	PLI	For R, synchronization (PLI) during ON periods was significantly lower than that during OFF periods in delta, theta, beta bands, and broad band For NR, no significant difference between ON and OFF periods Variation of seizure frequency with VNS correlated with PLI OFF/ON ratio in delta, theta, beta bands, and broad band
Bodin et al. (2015)	EEG	19 subjects (7 M, 14–54 years)	VNS treated for least 6 months	Acute ON vs. OFF, R vs. NR	PLI	The R had a significantly lower global synchronization level (PLI) in the broadband EEG than NR, especially marked in delta and alpha bands Across patients, EEG synchrony decreased (PLI) during ON compared to OFF
Vespa et al. (2021)	EEG	24 subjects (11 R: 4 M 34.1 ± 13.9 years, 13 NR: 4 M 33.2 ± 14.6 years)	R: 67.2 ± 61.5 months therapy, NR: 68.2 ± 56.6 months therapy	Acute ON vs. OFF, Awake vs. N2 NREM sleep	wPLI, GE, Band‐power	In the theta band, R showed significantly greater VNS‐induced desynchronization (wPLI) and decreased network integration (GE) during sleep compared to NR, but not during wakefulness For the band power analysis, no significant differences were detected between R and NR groups, ON vs. OFF conditions
Lanzone et al. (2022)	EEG	18 subjects (6 F, 43.9 ± 12.6)	VNS implanted within 4 years	Acute preVNS vs. VNS vs. postVNS	Band‐power, SWI, BtwC, GE	No significant differences found in EEG band power between the three VNS phases, and between R and NR Breakdown of Delta band activity during VNS (SWI) and persist several seconds postVNS Theta band: Decreased network integration (SWI) during VNS and recovery in the post‐VNS period Slow bands (Theta and Delta) connectivity becomes more integrated after VNS, but singular nodes appear to be less central in the network (GE, BtwC) Fast bands (Alpha and Beta) showed more scattered changes, alpha band showed decreased GE and BtwC during VNS
Wang et al. (2009)	EEG	8 subjects (4 M, 17–41 years),	Pre‐operation—24 months	Acute VNS activation vs. deactivation vs. reactivation, pre‐operation 3/6/12/24 months	IED	Significant progressive decreased in the number of IED on EEG with time staring 6 months Significantly more IED during VNS deactivation compared to VNS activation and reactivation
Germany Morrison et al. (2024)	EEG	28 subjects (13 M, 18–65 years, 13 R, 4 PR, 11 NR),	VNS treated for at least 6 months	Acute ON vs. OFF Dose‐ranging	wPLI, Band‐power	Lower stimulation intensity (0.75–1.25 mA) achieves the maximal desynchronizing EEG effect R and PR showed clear desynchronizing effects around their clinical intensity, while NR did not R showed distinct EEG patterns during ON phase compared to NR ○ Higher beta band power in frontrocentral region ○ Delta band changes were most significant at lower intensity ○ Alpha band differences appeared at 1.25 mA
Li et al. (2023)	MEG	14 subjects (7 M, 32 ± 8 years)	NA	Acute ON vs. OFF DRE vs. Health	wPLI CC, CPL SWI	DRE patients showed significantly increased functional connectivity (wPLI) compared to healthy controls across theta, alpha, beta, and gamma bands DRE patients exhibited more regular architecture and worse network efficiency in the theta, alpha and beta bands (CC, CPL, SWI) During VNS therapy, VNS significantly decreased functional connectivity (wPLI) in theta and alpha bands, and increased the network efficiency (CC, CPL, SWI) in the theta band
Qin et al. (2024)	sEEG	12 pediatric (3 R, 3 M; 1 PR, 1 M; 5 NR, 5 M; 3 WR, 2 M)	VNS treatment for 0.8–3.6 years	Acute ON vs. OFF R vs. PR vs. NR vs. WR	wPLI	There was a progressive trend of increased brain network synchronization as response changed from mild to WR for low‐gamma band across whole‐brain network WR showed significantly higher brain network synchronization at Epileptogenic zone and epileptogenic zone to propagation zone in both low‐gamma and high‐gamma bands
Koo (2001)	EEG	21 subjects (16 M, 14.1 ± 7 years)	Pre‐operation—12 months	Baseline/1/3/6/12 months	Waveform	In patients with active baseline epileptiform activity, the EEG showed clustering/synchronization of epileptiform activity after treatment. Over time, increased spike‐free duration and reduced paroxysm frequency and duration In patients with less active baseline EEGs, no obvious clustering/synchronization, but a significant progressive reduction in total spike counts at 3, 6 and 12 months. Several patients became spike‐free at 6 or 12 months
Ding et al. (2023)	EEG	10 subjects (5 M, 19.23 ± 7.37 years, 10 R)	Pre‐operation—24 months	Pre‐operation vs. 24 months	wPLI Degree, CC, CPL, GE	Increased functional connectivity (wPLI) between frontal and parieto‐occipital regions in the delta and beta bands, deceased connectivity within the frontal and between the frontal and parieto‐occipital regions in beta and gamma band after VNS therapy More efficient network organization in delta, theta and alpha bands and less efficient network in the gamma bands (D, CC, CPL, GE) after VNS therapy
Coa et al. (2022)	EEG	10 subjects (6 M, 27–61, 4 R)	Pre‐operation—12 months	Pre‐operation vs. 12 months, R vs. NR	PLI, Aperiodic parameters	After treatment, global and regional synchronization (PLI) decreased in delta and gamma bands and increased in alpha band for R, while NR showed no significant PLI changes R showed lower neuronal excitability (offset and exponent of the aperiodic parameters), while NR showed increased
Marrosu et al. (2005)	EEG	11 subjects (7 M, 26–44 years)	Pre‐operation—12 months	Pre‐operation vs. 12 months	Coherence, Interdependence Measure, Band‐power	VNS decreases synchronization (Coherence, Interdependence measure) of theta band VNS increases gamma power spectrum and synchronization (Coherence, Interdependence measure)
Fraschini et al. (2013)	EEG	10 subjects (4 M, 32–57 years, 5 R),	Pre‐operation—5/6 years	R vs. NR	PLI	R show significant desynchronization in the gamma band after 5 years of treatment; the desynchronization was not observed in other frequency bands
Babajani‐Feremi et al. (2018)	MEG	23 pediatric (14 R: 7 F 4.1 ± 4.6 years; 9 NR: 6 F, 6.1 ± 7.4 years)	VNS treated for at least 12 months	R vs. NR vs. Control	Modularity, Transitivity CPL	Epilepsy patient showed lower brain network segregation (modularity and transitivity) than healthy control. This lower segregation pattern also observed in NR as compared to R No difference was found for functional integration (CPL)
*Depression*						
Armitage et al. (2003)	EEG	7 subjects (7 F, 28–55 years)	Pre‐operation—10–12 weeks	Pre‐operation vs. 10–12 weeks	Sleep stage	Improvement on sleep architecture, decreased awake time and stage 1 sleep, increased stage 2 sleep The amplitude of ultradian sleep EEG increased by over 200%, particularly for delta rhythms
Kavakbasi et al. (2021)	MEG	1 subject (1 F, 60 years)	NA	Acute preVNS vs. VNS vs. postVNS	Band‐power	Reproducible reduction of alpha amplitude after the end of stimulation period in wide‐spread areas, but not during active stimulation

Abbreviations: BtwC, Betweenness Centrality; CC, clustering coefficient; CPL, characteristic path length; DRE, drug‐resistant epilepsy; EEG, electroencephalography; GE, global efficiency; IED, interictal epileptiform discharge; MEG, magnetoencephalography; NA, not available; NR, non responder; NREM, non‐rapid eye movement; PLI, Phase Lag Index; PR, partial responder; R, responder; sEEG, stereoelectroencephalography; SWI, Small World Index; wPLI, Weighted Phase Lag Index; WR, worse responder (seizure increase).

Clinically, VNS is an established adjunct therapy for drug‐resistant epilepsy: the iVNS was first FDA‐approved in 1997 for refractory epilepsy after pivotal trials demonstrated significant seizure reduction with active stimulation versus sham. Other than the invasive approach, the taVNS approach has also demonstrated promising outcomes with meaningful seizure reduction and better tolerability (Bauer et al. 2016). However, clinical outcomes show variability across patients, with response rates typically ranging from 40% to 65% (Abbasi et al. 2021; Clifford et al. 2024), indicating that while many patients benefit from iVNS therapy, a substantial proportion may experience limited improvement. This heterogeneous outcome is influenced by multiple interacting factors. Age at implantation shows mixed results in the literature, with some studies suggesting potentially better responses in younger patients (< 18 years) (Colicchio et al. 2010, 2012), while others report no clear age‐related difference or even favorable outcomes in older patients (Labar 2004). Seizure types also influence iVNS outcomes: simple‐partial seizures and auras demonstrate the most prominent responses to iVNS, while generalized tonic‐clonic seizures show the poorest result (Englot et al. 2011a). Epileptic falls represent a specific challenge, with Casazza et al. demonstrating that retropulsive tonic seizure showed the best outcomes, while tonic‐postural seizure had the worst result in response to iVNS (Casazza et al. 2006). iVNS has also been shown to be more efficacious in non‐idiopathic partial epilepsy than in non‐idiopathic and idiopathic generalized epilepsy (Bodin et al. 2016). In addition, the duration of epilepsy prior to VNS implantation appears inversely correlated with treatment success, suggesting that earlier intervention may enhance iVNS efficacy (Englot et al. 2011a, 2011b). Underlying epilepsy etiology also plays a role; one study reports that patients having lesional, particularly ischemia and tuberous sclerosis, show better outcomes (Colicchio et al. 2012) while another study reports non‐leisional epilepsy has better seizure reduction (Englot et al. 2016). Nonetheless, Tanganelli et al. reported that regardless of the type of seizure, iVNS is ineffective in patients with severe encephalopathy and high‐seizure frequencies (Tanganelli et al. 2002). Importantly, VNS demonstrates a cumulative therapeutic effect, with seizure reduction rates progressively improving from 46% at 3 months to 56% at 1 year and 62% at 2 years post‐implantation (Englot et al. 2011a). Similarly, a study of 21 epilepsy patients showed progressive increases in spike‐free intervals and decreases in epileptiform activity over 12 months of VNS treatment, with those having active baseline epileptiform activity demonstrating significant synchronization of spikes followed by desynchronization (*p* < 0.05) and progressive decreases in spike duration and frequency (*p* < 0.01), while those with less active baseline EEGs showed progressive decreases in spike numbers with increasing significance over time (*p* < 0.004 at 3 months, *p* < 0.008 at 6 months, and *p* < 0.004 at 1 year) (Koo 2001). In a long‐term study, VNS was demonstrated to be effective over a period up to 17 years, and some patients responded only after 4 or more years, emphasizing that lack of early improvement does not imply ineffectiveness (Chrastina et al. 2018). Additionally, optimal parameter settings for iVNS are not well defined and are typically individualized through titration (Abbasi et al. 2021; Thompson et al. 2021). Collectively, these findings underscore that the VNS response is influenced by multiple complex and interacting factors, highlighting the need for individualized treatment approaches and careful patient selection to optimize therapeutic outcomes.

#### 5.1.2 Stroke Rehabilitation Motor Recovery

VNS exerts pro‐plasticity that is highly relevant for post‐stroke recovery. By activating brainstem centers (NTS, LC, dorsal raphe, etc.), VNS triggers a widespread release of neuromodulators like NE, ACh, and 5‐HT that facilitate synaptic plasticity and cortical reorganization (Hu et al. 2024). This neuromodulator surge creates a brain state favorable to relearning motor skills after stroke, promoting the formation of new connections. Preclinical studies demonstrate that pairing VNS with rehabilitative training can markedly amplify synaptic connectivity in impaired motor circuits (Meyers et al. 2018; Hu et al. 2024). Such stimulation‐associated plasticity appears to target the use‐dependent cortical representation of movement. VNS paired with forelimb exercise in rats led to substantial expansion of the motor cortex map for the trained forelimb (Hulsey et al. 2016). By engaging these plasticity pathways, VNS can effectively “prime” the injured brain to rewire itself (Hulsey et al. 2016). Furthermore, VNS‐induced plasticity is not limited to motor pathways; even long‐established sensory deficits can be improved. For example, a case study reported that chronic stroke survivors with severe hand sensory loss experience significant restoration of sensation after undergoing VNS tactile retraining (Kilgard et al. 2018). In addition, VNS also exhibits an anti‐inflammatory role in alleviating cerebral I/R injury reducing resultant ischemic penumbra (Du et al. 2024; Zhao et al. 2019) and promotes parasympathetic activity to alleviate cardiac dysfunction post stroke in animal models (Wang et al. 2025).

Growing clinical evidence supports the therapeutic value of VNS as an adjunct to stroke rehabilitation: VNS‐REHAB trial demonstrates superior improvements in upper‐limb function (active *N* = 53 vs. sham *N* = 55): 5 vs. 2.4 increment on Fugl‐Meyer Assessment Upper Extremity (FMA‐UE) score (*p* = 0.001) (Dawson et al. 2023). In 2021, the FDA approved VNS therapy for improving upper‐limb motor function in chronic ischemic stroke patients. Approximately 73% of patients who received VNS in early trials maintained a clinically significant improvement in arm motor scores 1 year after therapy (Hu et al. 2024). While most clinical trials have focused on ischemic stroke, there is growing evidence that VNS can aid recovery in hemorrhagic stroke as well (Cummins et al. 2024; Hays et al. 2014), potentially by promoting neuroplasticity and improving cerebral blood flow and oxygen metabolism. In addition, early‐phase trials report enhanced motor gains and functional recovery with noninvasive approaches, offering a potentially accessible alternative for broader stroke populations (Wu et al. 2020). While VNS provides beneficial effects in improving motor function for stroke rehabilitation, its impact on fine motor skills, such as the box and block test and the Nine‐Hole Peg test remains limited (Jiang et al. 2020).

#### 5.1.3 Traumatic Brain Injury

Still at its early stage, the VNS effect on traumatic brain injury (TBI) is primarily studied in animal studies. Systematic reviews by Zhang et al. and Neren et al. summarized the potential neuroprotective mechanisms of action of VNS on TBI (Neren et al. 2016; Zhang, Li, et al. 2022). Primarily, VNS mitigates oxidative stress by reducing oxidative markers like malondialdehyde (MDA), enhancing antioxidant enzymes, including glutathione (GSH), superoxide dismutase (SOD), and catalase (CAT) in the brain, and diminishing neuronal apoptosis (Tang et al. 2020). VNS activates α7nAChR on microglia to inhibit proinflammatory cytokines, thereby preventing neuroinflammation‐induced secondary brain tissue damage, edema, and cell necrosis following traumatic injury (Tang et al. 2020; Schimmel et al. 2017). Additionally, VNS may contribute to anti‐inflammatory pathways through a ghrelin‐mediated mechanism where VNS stimulates ghrelin release while ghrelin reciprocally activates VN, leading to reduced proinflammatory cytokines (Bansal et al. 2012). Of particular note, VNS preserves BBB integrity, reducing edema through lowered vascular permeability and downregulation of Aquaporin (AQP‐4) (Lopez et al. 2012). This preservation of BBB integrity if treated early may further reduce the likelihood of comorbid TBI‐related diseases such as post‐traumatic headache and potentially associated mental health disorders (da Silva Fiorin et al. 2024). Furthermore, VNS enhances neurotransmitter signaling, particularly promoting GABAergic activity, thereby reducing glutamate‐mediated excitotoxicity, improving consciousness and enhancing cognitive function (Neren et al. 2016; Zhang, Li, et al. 2022). VNS has also been demonstrated to decrease intracranial pressure in animal studies, which may potentially improve TBI outcomes (Neren et al. 2016).

VNS has shown early promise for enhancing recovery after TBI. A prospective pilot clinical trial by Shi et al. investigated the use of iVNS in twelve patients with severe TBI and impaired consciousness over 18 months and demonstrated that vagal stimulation promotes arousal and improves consciousness (Shi et al. 2013). More recently, a 2020 feasibility study using taVNS in five patients with diffuse axonal injury observed clinical improvements in the revised Coma Recovery Scale (CRS‐R) in three out of five patients after 8 weeks of treatment (Hakon et al. 2020).

#### 5.1.4 Disorders of Consciousness

VNS engages several neurobiological pathways that can counteract brain injury‐induced impairments in arousal, cognition, neural integrity, and disorders of consciousness (DoC). In a case study from a vegetative patient, iVNS promotes alertness and attention by enhancing theta band activity in multiple brain regions, including the right inferior parietal cortex and parieto‐temporal‐occipital border, as well as bilateral occipito‐parietal, inferior temporal, and fronto‐central regions, implying that VNS may reorganize large‐scale brain connectivity (Corazzol et al. 2017). Stimulation increases metabolic activity in the thalamus and enhances neural firing in brain regions associated with arousal and alertness (Corazzol et al. 2017). An fMRI analysis also revealed that taVNS enhanced connectivity within the default mode network in a vegetative female patient (Yu, Yang, et al. 2017). In addition, VNS can upregulate wake‐promoting peptides like orexin‐A in the PFC (Dong and Feng 2018). By stimulating these arousal networks, including the ascending reticular activating system (ARAS) through the LC and raphe nuclei in the upper brainstem, VNS is thought to reinvigorate the thalamocortical circuits that underpin consciousness, which is evidenced by increased thalamic and cortical activation after VNS (Briand et al. 2020).

In parallel, VNS may help neural reorganization and recovery of DoC after brain injury by enhancing neuroplasticity (Biggio et al. 2009), activating neurotrophic factors (Follesa et al. 2007), and augmenting cerebral blood flow to injured brain regions, improving perfusion and delivering oxygen/glucose to support recovery (Yu et al. 2021). Another benefit of VNS in brain injury is its influence on neuroinflammatory and neuroprotective pathways. Secondary injury processes after TBI, including excitotoxicity, inflammation, oxidative stress, and brain cell apoptosis, often exacerbate neural damage and impede recovery. VNS can dampen these harmful cascades via the VN's anti‐inflammatory reflex (Tang et al. 2020; Dong et al. 2023). In addition, a case report revealed a longitudinal increase in HRV‐HF power throughout the 6‐month taVNS treatment, suggesting enhanced parasympathetic control that paralleled improved conscious level (Osińska et al. 2022).

Early clinical evidence suggests that VNS is potentially effective in treating patients with DoC, including etiology from TBI, hemorrhage, stroke, hypoxic ischemic encephalopathy, and diffuse axonal injury (Dong et al. 2023). However, its effectiveness varies significantly depending on the patient's baseline consciousness (Dong et al. 2023). Case reports show that both iVNS (Corazzol et al. 2017) and taVNS (Osińska et al. 2022) may help on conscious recovery, as measured by CRS‐R scores. Notably, patients with minimally conscious state (MCS) tend to show more CRS‐R improvement compared to those in vegetative state/unresponsive wakefulness syndrome (VS/UWS) (Zhou et al. 2023; Noé et al. 2020). One pilot study reported no significant improvement in CRS‐R scores immediately following treatment, but observed notable changes in brain activity patterns: MCS patients showed alterations in both delta and beta frequency bands across multiple brain regions and enhanced connectivity between different brain areas, while VS patients demonstrated more limited changes confined to localized brain regions (Yifei et al. 2022). However, the field lacks large, high‐quality randomized controlled trials (RCT) with adequate follow‐up periods and statistical rigor. More robust, multicenter studies are needed to validate VNS as a treatment for DoC.

#### 5.1.5 Alzheimer's and Parkinson's Diseases

VNS has also gained interest as a neuromodulatory therapy for neurodegenerative disorders like Alzheimer's disease (AD) and Parkinson's disease (PD), especially in symptomatic stages of these illnesses. Both AD and PD are characterized by progressive neuronal loss and dysregulation of neurotransmitter systems, leading to cognitive and motor impairments. With limited effective pharmacotherapies for reversing cognitive decline in AD or halting PD progression, VNS offers a non‐pharmacological approach to engage broad brain networks and neurochemical pathways that counteract pathological neurodegenerative disease mechanisms (Vargas‐Caballero et al. 2022; Broncel 2024; Zhang et al. 2023; Eissazade et al. 2025).

In AD, LC degeneration and basal forebrain cholinergic deficits contribute to attentional and memory impairments, and VNS‐driven bursts of NE and ACh may acutely improve alertness, working memory, and learning by engaging the frontoparietal attention network (Kaczmarczyk et al. 2018; Aniwattanapong et al. 2022). Similarly, in PD, early cognitive deficits and other non‐motor symptoms are believed to be due to LC degradation, which often precedes the degeneration of dopaminergic neurons of the substantia nigra (Rietdijk et al. 2017). Although VNS does not directly reverse the primary DA deficit, the enhanced noradrenergic and cholinergic tone can modulate basal ganglia and cortical circuits and, in turn, improve the hallmark motor symptoms of PD (Farrand et al. 2017; Mondal et al. 2024). Another key mechanism of VNS in neurodegeneration is its anti‐inflammatory and neuroplastic effects. In AD and PD, chronic neuroinflammation accelerates neuronal injury and proteinopathies. Preclinical studies of AD indicate that VNS can inhibit inflammatory cascades like the NLRP3 inflammasome in the hippocampus, resulting in fewer amyloid‐β deposits and improved neuronal survival (Cai et al. 2019; Yu et al. 2023). Likewise, in PD models, VNS drives central anti‐inflammatory signals and the release of neurotrophic factors (like BDNF) that improve locomotor recovery and neuronal resilience (Evancho et al. 2024; Mondal et al. 2024; Eissazade et al. 2025).

In AD, initial clinical evidence from iVNS has shown encouraging results. Sjögren et al. demonstrated that 7 of 10 AD patients showed improvement or stability in cognitive measures (Alzheimer's Disease Assessment Scale‐Cognitive Subscale [ADAS‐Cog] and Mini‐Mental State Examination [MMSE]) after 6 months of iVNS treatment (Sjögren et al. 2002). An extended follow‐up of the study with 17 patients after 1 year revealed that 41% and 71% of the patients improved in ADAS‐cog and MMSE tests, along with evidence of lowered tau protein level in cerebrospinal fluid, an important biomarker for cognitive improvement (Merrill et al. 2006). In a taVNS study, a larger RCT including 60 patients with mild cognitive impairment due to AD also demonstrated improvement in Montreal cognitive assessment‐basic score (MoCA‐B) after 12 weeks of therapy compared to the sham group (*p* = 0.033), with the taVNS group also showing significant improvement in immediate recall (*p* < 0.001) and delayed recall (*p* < 0.0001) (Wang et al. 2022). In contrast, Mertens et al. demonstrated no effect of taVNS on verbal memory performance in either older or younger groups of healthy volunteers (Mertens et al. 2020). Vargas‐Caballero et al. attribute this negative result to insufficient LC modulation due to the stimulation protocol, highlighting the importance of adequately activating the LC‐noradrenergic network to achieve memory enhancement effects (Vargas‐Caballero et al. 2022). Importantly, Helmstaedter et al. documented impaired figural memory in 11 epilepsy patients following high‐intensity acute VNS. Despite the reversible nature of the effect and study limitations including small sample size and absence of sham control, the findings highlight the need to systematically select VNS parameters in each treated population and further underscore the need for controlled studies in the AD population (Broncel 2024; Helmstaedter et al. 2001).

In PD, VNS has shown modest but consistent improvements in motor symptoms, especially on gait parameters (Shan et al. 2025; Zhang et al. 2023). The non‐motor effects of VNS in PD remain unclear due to the lack of studies in this area (Shan et al. 2025; Evancho et al. 2024). Some trials report improvements in cognition—specifically in executive function, processing speed, and inhibitory control (Lench et al. 2023; Marano et al. 2022) while others report no impact on cognition (Mondal et al. 2024) or decline in verbal fluency (Lench et al. 2023). The impact of VNS on other non‐motor symptoms in PD, including mood, fatigue, sleep, and GI disturbance, is vastly understudied with mixed findings; some studies indicate VNS improves non‐motor symptoms (Mondal et al. 2024; Kaut et al. 2019) while others report no impact (Lench et al. 2023). Collectively, it undergirds the need for future research focusing on the impact of VNS on non‐motor symptoms in PD (Evancho et al. 2024).

#### 5.1.6 Tinnitus

Tinnitus is primarily caused by maladaptive neural plasticity in the auditory cortex, potentially resulting from reduced output from the affected cochlear region that leads to loss of lateral inhibition and increased neural synchrony in the central auditory system. This neural reorganization can occur due to factors like hearing loss, noise exposure, or neurological changes that disrupt normal auditory processing and create a persistent phantom sound perception (Yakunina and Nam 2021). By pairing auditory stimuli with VNS‐driven neuromodulator release, it is possible to direct plastic changes in auditory pathways, rewiring aberrant circuits and suppressing the phantom percept in rats and humans (Yakunina and Nam 2021; Engineer et al. 2013; Conlon et al. 2022; De Ridder et al. 2014). The therapy also reduced pathological functional connectivity between auditory regions and limbic areas that are linked to tinnitus distress. Uniquely, auricular stimulation may directly influence auditory brainstem structures via the somatosensory‐auditory connections: the medullary somatosensory nuclei project to the dorsal cochlear nucleus. This means that stimulating the ear's vagal fibers can modulate activity in the cochlear nucleus, a key hub in tinnitus generation, providing a plausible anatomical route for taVNS to ameliorate tinnitus at its source (Yakunina and Nam 2021). Additionally, VNS affects limbic structures, with neuroimaging studies consistently showing deactivation of the amygdala, hippocampus, and cingulate cortex, which may address tinnitus distress, given that neuroimaging studies have supported the abnormally strong connection between the auditory and limbic systems in tinnitus patients (Yakunina and Nam 2021). However, while promising, current VNS tinnitus treatment research is still preliminary: most studies have methodological limitations, small sample sizes, and lack robust clinical evidence (Yakunina and Nam 2021). Alternative approaches leveraging trigeminal nerve stimulation or somatosensory nerve stimulation paired with auditory stimuli, known as bimodal neuromodulation, have achieved greater success and evidence in recent years, with the first FDA‐approved bimodal device treatment for tinnitus occurring in 2023 (*Lenire Tinnitus Treatment Device* developed by Neuromod Devices, FDA ref.: DEN210033) that has demonstrated efficacy and safety in several large‐scale clinical trials and recent real‐world patients (Conlon et al. 2022; McMahan and Lim 2025; Boedts et al. 2024; Markovitz et al. 2015).

#### 5.1.7 Migraine, Cluster Headache and Chronic Pain

Primary headache disorders have been a focus of VNS application. VNS can modulate trigeminovascular pathways implicated in migraine and cluster headache, thereby altering pain signaling in the brainstem. An FDA‐cleared device in 2015, *GammaCore*, offers tcVNS for the treatment and prevention of cluster headache, following its demonstration of significantly reducing headache attacks in the PREVA study (active *N* = 45, control *N* = 48: −5.9 vs. −2.1 attacks per week, *p* = 0.02) (Gaul et al. 2016). Mechanistically, in preclinical models, VNS inhibited cortical spreading depression, a known trigger for migraine aura, and potentially alters neurotransmitter dynamics, including enhancing GABAergic and 5‐HT transmission and reducing glutamate levels (Chen, Ay, et al. 2016; Ayata and Lauritzen 2015). fMRI further suggests that VNS modulates key brain regions such as the motor‐related thalamus subregion, ACC, and postcentral gyrus (Zhang et al. 2021). Additionally, transcutaneous VNS likely exerts its effects through skin pain receptors and brain stem‐mediated descending inhibitory pain pathways, dampening trigeminal nerve excitability (Song et al. 2023; Tassorelli et al. 2018; Diener et al. 2019). In cluster headache, the VNS works through two primary mechanisms: first, by inhibiting vagal afferents to the trigeminal nucleus caudalis (TNC), which reduces pain signal transmission, and second, by modulating inhibitory neurotransmitter release, specifically decreasing glutamate levels in the TNC (Gaul et al. 2016). Notably, the EVENT study (active *N* = 39 vs. sham *N* = 29) showed that VNS effectiveness for chronic migraine is time‐dependent: minimal benefit was observed during the initial 2‐month period (−1.4 vs. −0.2 monthly headache days, *p* = 0.56), but participants achieved reduction on an average of 7.9 monthly headache days (*p* < 0.01) after 8 months of therapy, indicating that sustained prophylactic use is necessary for optimal treatment (Silberstein, Calhoun, et al. 2016). However, the PREMIUM RCT (active *N* = 165, sham *N* = 167) for episodic migraine prevention showed mixed results: the primary endpoint analysis found tcVNS reduced mean monthly migraine days by 2.26 days compared to 1.8 days with sham treatment over 12 weeks (*p* = 0.15) in the intent‐to‐treat population. The authors attributed this to inadvertent vagal activation in the sham device and suboptimal patient adherence. Post hoc analysis of treatment‐adherent patients (> 67% adherence, 83.6%–83.8% of participants) revealed significant superiority of tcVNS over sham for monthly migraine days (−2.27 vs. −1.53, *p* = 0.043), headache days (−2.85 vs. −1.99, *p* = 0.045), and acute medication use (−1.94 vs. −1.14, *p* = 0.039) (Diener et al. 2019). The subsequent PREMIUM II RCT (active *N* = 56, sham *N* = 57), employing a completely inactive sham device to address PREMIUM I's methodological limitation, similarly failed to achieve statistical significance for the primary endpoint (−3.12 vs. −2.29 monthly migraine days, *p* = 0.2329), likely due to COVID‐19‐related early termination with 60% reduced enrollment. However, PREMIUM II demonstrated a significant responder rate with 44.87% of active participants achieving ≥ 50% reduction in migraine days versus 26.81% with sham (*p* = 0.0481) (Najib et al. 2022). Both trials emphasize that long‐term, consistent tcVNS application with high adherence is critical for therapeutic success, particularly in patients with migraine with aura.

In an fMRI healthy control study, tcVNS stimulation produces a triphasic post‐stimulation pattern with mid‐phase activation (7–9 min) of the hypothalamus, amygdala, and pain‐modulatory brainstem regions, including the PAG. This ~10‐min delayed hypothalamic activation correlates with an 11‐min delayed onset of cluster headache relief observed in clinical trials, while deactivation of the spinal trigeminal nucleus caudalis provides a mechanism for reducing trigeminal nociception in headache disorders (Frangos and Komisaruk 2017).

Intriguingly, the MOXY study (*N* = 24) revealed that while tcVNS significantly reduced migraine severity and frequency in female patients, salivary IL‐1β levels paradoxically increased after treatment, suggesting that VNS may provide symptomatic relief without resolving underlying neuroinflammatory processes, particularly in patients with aura, where cortical spreading depression continues to activate glial cells and perpetuate immune responses (Boström et al. 2019). Additionally, given that HRV is decreased in headache patients, VNS may help restore autonomic balance, though further validation is required (Koenig et al. 2016).

For chronic pain, VNS engages multiple pathways to modulate pain perception, including recruitment of descending inhibitory pathways, attenuation of central sensitization, plasticity improvement in brainstem pain centers, and neuro‐immune interaction, which collectively dampen nociceptive signaling (Shao et al. 2023). With tcVNS, patients report some discomfort at the highest voltages; this painful sensation could potentially recruit additional DNIC circuits. Of note, this pathway of activating the DNIC has also revealed successful treatments for migraine with the recent FDA clearance of the *Nerivio* device (Theranica, Israel; FDA initial ref.: DEN180059), which is applied on the patient's upper arm for acute and/or preventive treatment of migraine with or without aura.

On the basis of two systematic reviews examining VNS for chronic pain management, the therapy demonstrates promising outcomes (Costa et al. 2024; Duff et al. 2024). Costa et al. reviewed 15 studies of 684 participants, which showed VNS produces a small to moderate effect size for reducing pain, with both taVNS and tcVNS showing similar effectiveness and larger effect sizes compared to the control group (Costa et al. 2024). Duff et al. reviewed 42 studies and found that paVNS demonstrated superior effectiveness compared to transcutaneous methods, particularly for chronic pain, abdominal pain, and migraine conditions (Duff et al. 2024).

### 5.2 Psychiatric Disorders

#### 5.2.1 Depression

VNS profoundly impacts brain networks associated with mood regulation through targeted modulation of key neural regions. Chronic VNS therapy demonstrates significant changes in brain activity, notably reducing hyperactivity in vmPFC, a region typically overactive in depressed patients (Nahas et al. 2007). The insula activation from VNS is dynamic and severity‐dependent, with acute VNS producing greater right insula activation in more severely depressed participants. VNS also impacts limbic structures, particularly the amygdala and hippocampus, which are central to fear and stress responses (Nemeroff et al. 2006; Fang et al. 2016; Nahas et al. 2007; Mu et al. 2004; Bohning et al. 2001; Conway et al. 2006; Zobel et al. 2005) (Table 3). Beyond regional effects, VNS also influences large‐scale brain networks that are dysregulated in depression. Notably, the default mode network, a network associated with self‐referential thought (i.e., rumination) and often hyperactive in depression, appears to be modulated by VNS, and these connectivity changes correlated with symptom improvement (Fang et al. 2016). MEG revealed that acute VNS induced a significant reduction in alpha activity across widespread cortical regions immediately following stimulation, potentially representing an antidepressant mechanism given the established association between elevated alpha power and depressive/anxious symptoms (Kavakbasi et al. 2021). Chronically, VNS could improve sleep architecture by restoring the strength of ultradian sleep EEG rhythms and acting as a pacemaker for neural synchronization (Table 4) (Armitage et al. 2003). Neuroimaging studies reveal that VNS produces dynamic changes in brain metabolic activity, with initial right insula activation followed by gradual deactivation of regions associated with negative emotional processing. The mechanism is characterized by a non‐linear response, where brain network modifications occur slowly over months, suggesting a unique approach to treating depression that differs from rapid pharmacological interventions (Conway et al. 2013). Neurochemically, clinical and preclinical research suggests that VNS modulates multiple neurotransmitter systems involved in the pathogenesis of depression, including NE, 5‐HT, GABA, and glutamate (Ressler and Mayberg 2007; Müller et al. 2018). In addition, VNS influences the neuromodulator system relevant to mood, particularly DA and ACh. Emerging evidence links VNS to modulation of midbrain dopaminergic circuits that govern motivation and reward functions, often blunted in depression (Manta et al. 2013; Conway et al. 2012).

Depression has been associated with a dysregulation of central ACh signaling, sometimes described as an “adrenergic‐cholinergic imbalance” in mood disorders (Janowsky et al. 1983). VNS likely influences central cholinergic circuits indirectly via its brainstem connections (Horinouchi et al. 2024). More importantly, by dampening proinflammatory cytokine production, VNS addresses the inflammatory underpinnings of depression, a condition intrinsically connected to a persistent state of systemic inflammation (Das 2007). For long‐term consideration, recent pilot studies found that chronic VNS therapy led to a significant reduction in several plasma cytokines after 6 months to 4 years of treatment (Lespérance et al. 2024; Kavakbasi et al. 2024). Lastly, VNS appears to normalize the hyperactive HPA axis characteristic of depression, reducing elevated stress hormone levels and restoring typical neuroendocrine stress system responses through mitigating exaggerated ACTH release (O'Keane et al. 2005). The same study found that certain elevated cortisol measures in these patients also declined to the normal range during VNS treatment, and notably, the degree of HPA axis attenuation correlated with clinical improvement in chronic depression (O'Keane et al. 2005).

Therapeutically, VNS gained FDA approval in 2005 as a groundbreaking adjunctive treatment for severe treatment‐resistant depression, offering a novel therapeutic approach for patients who have not responded to conventional interventions (Nemeroff et al. 2006). While early clinical trials showed modest short‐term results, a long‐term study (*N* = 795: 494 VNS + treatment as usual, 301 treatment as usual alone) revealed more significant benefits: cumulative 5‐year response rates reached 67.6% versus 40.9% (*p* < 0.001), with 43.4% of patients achieving full remission versus 25.7% (*p* < 0.001) from previously unresponsive depressive episodes, and median time to response being 12 months versus 48 months (*p* < 0.001) (Aaronson et al. 2017). Conway and colleagues in 2013 indicated that the antidepressant effects of VNS are typically delayed and many patients responded to treatment after 6 months. In these treatment‐resistant depressive patients, VNS led to a reduction in cerebral glucose metabolism in right dorsal lateral PFC at 3 months, with levels returning to baseline after 12 months of treatment, highlighting early reduction possibly facilitating later increases in dopaminergic activity (Conway et al. 2013). The recent RECOVER trial by the Conway group in 2025, a large RCT with 493 patients (251: VNS + treatment as usual, 244: sham + treatment as usual) having an average of 13.3 failed antidepressant treatments, found that while 12‐month VNS treatment did not meet its primary Montgomery‐Åsberg Depression Rating Scale (MADRS) endpoint (18.9% vs. 16.3%, *p* = 0.137) over 12 months, but revealed significant benefits on multiple secondary measures, including Clinician Global Inventory‐Impression (CGI‐I) ratings (26.7% vs. 18.2%, *p* = 0.004) and self‐reported Quick Inventory of Depressive Symptomatology (25.2% vs. 19.8%, *p* = 0.049) (Conway et al. 2025). Most notably, partial response rates (≥ 30% symptom reduction) were more sensitive to treatment effects, with CGI‐I response reaching 53.8% vs. 39.8% (*p* < 0.001), suggesting that achieving partial improvement represents a clinically meaningful goal. In aggregate, it highlights the need for further research to identify optimal patient profiles across their disease span, that is, early depression versus chronic depression, that may then guide treatment parameters (Conway et al. 2013).

Of note, although VNS is FDA‐approved as adjunctive therapy for both epilepsy and depression, it has been explored as monotherapy for epilepsy (Rose and Tao 2011), whereas it typically remains combined with pharmacotherapy for depression.

#### 5.2.2 Anxiety Disorders and PTSD

VNS engages multiple pathways that counteract the neurobiological dysregulation seen in anxiety disorders, including generalized anxiety disorder (GAD), panic disorder, social anxiety disorder, and PTSD. These conditions are marked by hyperactive fear circuitry, chronic stress hormone imbalances, autonomic dysregulation, and often elevated inflammation.

Anxiety disorders and PTSD are characterized by overactivity in fear‐related brain regions, notably the amygdala and related limbic areas, coupled with insufficient top‐down regulation by the PFC. VNS has demonstrated the ability to suppress hyperactive limbic circuitry and strengthen prefrontal inhibitory pathways. Importantly, VNS appears to enhance the vmPFC‐amygdala circuit involved in fear extinction (Peña et al. 2014; Szeska et al. 2020; Wittbrodt et al. 2020; Li et al. 2020). In a preclinical model, VNS‐treated rats show significantly less freezing after a single extinction training session, and VNS reverses the typical synaptic plasticity pattern in the vmPFC to amygdala pathway, converting LTD into LTP, which indicates strengthened safety‐signal transmission. Such plasticity in the vmPFC‐amygdala pathways facilitates the formation of new non‐fearful associations, a critical process for recovery in PTSD and anxiety (Peña et al. 2014), and has been proven to have a long‐term inhibitory effect in humans (Szeska et al. 2020). In parallel, the surge of NE, ACh, 5‐HT, and BDNF primes the fear circuit for plasticity and learning: By boosting synaptic plasticity in the basolateral and amygdala and hippocampal formation, VNS may promote extinction of fear memories and reorganization of emotional networks (Peña et al. 2014; Shin et al. 2019). The Nobel et al. study provides direct experimental validation, showing that VNS paired with extinction training enabled PTSD model rats to achieve fear remission by day 5 while the sham group never reached remission, and importantly, prevented fear reinstatement following stress reminder, demonstrating long‐term strengthening of extinction memories (Noble et al. 2017).

In addition, patients with GAD or panic disorder often exhibit elevated baseline cortisol or an exaggerated stress hormone release, whereas PTSD can involve a maladaptive cortisol profile and blunted feedback sensitivity. Through HPA‐axis activity modulation, vagal stimulation can influence CRH release and downstream cortisol secretion, providing a brake on stress hormone output (Camici et al. 2024; Bremner, Gurel, Wittbrodt, et al. 2020). Another hallmark of GAD and PTSD is autonomic dysregulation—typically a dominance of sympathetic activity and relative vagal underactivity. Clinically, this manifests as tachycardia, palpitations, sweating, hyperventilation, and heightened startle reactivity during anxiety and panic episodes, whereas VNS directly provides an autonomic balancing (Gurel et al. 2020; Bremner, Gurel, Wittbrodt, et al. 2020). In conditions like panic disorder, where sudden surges of sympathetic activation trigger acute attacks, this balancing influence of VNS may prevent the cascade of cardiovascular and respiratory symptoms. In PTSD and GAD, increased vagal tone correlates with better emotion regulation and lower baseline anxiety; accordingly, VNS‐induced gains in HRV are viewed as therapeutic biomarkers. Similar to depression, emerging evidence links GAD and PTSD with a state of low‐grade inflammation—patients often exhibit elevated proinflammatory cytokines and inflammatory biomarkers. Heightened inflammation can exacerbate anxiety by affecting neurotransmitters and brain circuits (Bremner, Gurel, Jiao, et al. 2020; Bremner, Gurel, Wittbrodt, et al. 2020). The CAP pathway of VNS provides a direct effect of attenuating systemic inflammatory response. A recent RCT in patients (*N* = 30) with PTSD provides evidence of VNS's anti‐inflammatory action in a clinical anxiety context. Bremner et al. found that VNS blocked stress‐induced surges in inflammatory cytokines: active tcVNS significantly blocked stress‐induced IL‐6 (*p* = 0.046) and IFN‐γ (*p* = 0.032) elevations in PTSD patients exposed to personalized traumatic scripts, whereas the sham stimulation group showed marked cytokine increases (Bremner, Gurel, Jiao, et al. 2020). The inflammation‐modulating effect of VNS may yield calmer brain physiology: elevated inflammatory signals can activate microglia and HPA‐axis stress responses, while VNS is likely able to reduce neuroinflammation‐mediated perpetuation of central and peripheral inflammation, thereby reducing perpetual HPA‐axis stress responses.

#### 5.2.3 Attention‐Deficit/Hyperactivity Disorder

Attention‐deficit/hyperactivity disorder (ADHD) is characterized by impairments in attention regulation, impulse control, and executive function, linked to disrupted fronto‐cortical and fronto‐striatal circuits. Both pediatric and adult ADHD patients show functional abnormalities in the prefrontal network (e.g., reduced dorsolateral prefrontal and ACC activity); in aggregate, it is thought these abnormalities undermine cognitive control, key for focus required for task switching (Zhi et al. 2024). A key mechanism of VNS in ADHD is the activation of the LC‐NE system to optimize cortical arousal. This NE surge increases excitatory drive in widespread cortical and subcortical regions and specifically activates the PFC, which is underactive in ADHD (Zhi et al. 2024). Vagal stimulation activates cholinergic neurons in the basal forebrain alongside the LC, increasing cortical ACh that enhances signal encoding and plasticity in the PFC and hippocampal circuits. This neuromodulation facilitates LTP and strengthens synaptic connectivity, particularly in memory‐related (e.g., *N*‐back) and attention‐related (psychomotor vigilance task) tasks (Zhi et al. 2024). Electrophysiologically, VNS increases cortical arousal and attenuates alpha oscillations, which are linked to the default mode network (Chen, Lu, and Hu 2023). This reduction may help improve attention and cognitive performance, especially in ADHD patients who often have an overactive default mode network (Duffy et al. 2021).

In parallel, animal studies demonstrate that chronic VNS elevates extracellular NE levels in the PFC and hippocampus, with corresponding activation of post‐synaptic α2A receptors in pyramidal neurons, which are the same receptor pathways targeted by ADHD medications like guanfacine to strengthen prefrontal function. NE stimulation of α2A receptors suppresses cAMP‐mediated ion currents and enhances PFC microcircuits, improving the efficacy of synaptic inputs and sustaining working memory representation (Zhi et al. 2024). In addition to noradrenergic pathways, VNS influences dopaminergic transmission and fronto‐striatal circuits that are relevant to ADHD's motivational and executive deficits. By modulating the midbrain DA system, VNS may help normalize the DA underactivity observed in ADHD (Del Campo et al. 2011). Importantly, VNS has been found to engage key nodes of the brain's executive and reward networks, which are often dysregulated in ADHD (Del Campo et al. 2011).

VNS also has an acute, positive effect on response inhibition to suppress impulse actions. During the Go/No‐GO trial, VNS increased activation of the right inferior frontal gyrus, a critical node for motor response inhibition. This suggests vagal stimulation improves the recruitment of frontal inhibitory circuits that ADHD patients often underutilize (Zhi et al. 2024). VNS also modulates cognitive flexibility, the ability to shift attention and adapt to changing rules or feedback, which may be beneficial for ADHD patients. Evidence indicates that VNS can enhance cognitive flexibility in tasks requiring set‐shifting and reversal learning. These pro‐flexibility effects are thought to result from VNS engaging LC‐NE‐innervated PFC and hippocampus circuits, which are crucial for processing new information and updating behavioral strategies based on situational demand (Zhi et al. 2024). Clinically, VNS has been shown to improve attention and alleviate symptoms of depression in ADHD individuals. taVNS combined with mobile‐assisted games and relaxation exercises significantly improved sustained attention parameters in young adults at high risk for ADHD in a RCT (active *N* = 30, control *N* = 30), with the active group showing significant improvement in reaction time (*p* = 0.001), missed target (*p* = 0.013), and non‐target responses (*p* = 0.001), while the control group only showed significant improvement in non‐target responses (*p* = 0.013) (Yildiz et al. 2024).

#### 5.2.4 Other Psychiatric Disorders

##### 5.2.4.1 Autism Spectrum Disorder

VNS has been explored in individuals with autism spectrum disorder (ASD), mostly in those with co‐occurring refractory epilepsy (van Hoorn et al. 2019). A retrospective registry analysis of patients with ASD (*N* = 77) and intractable epilepsy without ASD (*N* = 315) who received implanted VNS reported seizure reductions comparable to those in non‐ASD patients, along with some psychosocial benefits. Notably, caregivers observed improvements in mood and overall quality of life (*p* = 0.04) at 12 months post‐VNS. Although these findings come from open clinical data (and primarily target epilepsy), they suggest that chronic VNS might positively influence affective or behavioral symptoms in ASD. Dedicated trials in ASD without epilepsy are still lacking, and thus conclusions remain tentative (Levy et al. 2010).

##### 5.2.4.2 Substance Use Disorder

Promising early work provides support that noninvasive VNS may alleviate withdrawal‐related symptoms in opioid use disorder (OUD) and in alcohol use disorder (AUD). In OUD, a double‐blind, sham‐controlled (active *N* = 10, sham *N* = 11) study showed reductions in behavioral and physiological symptoms of opioid withdrawal, including subjective opioid withdrawal (*p* = 0.047), pain (*p* = 0.045), distress (*p* = 0.004), and lower heart rate (*p* = 0.026), following tcVNS compared to sham stimulation (Gazi et al. 2022). A subset of this cohort also participated in neuroimaging scans, which demonstrated increased activation in prefrontal brain regions in response to opioid cues following active tcVNS (Rahman et al. 2025). In 2017, the FDA approved a percutaneous VNS (*NSS‐2 Bridge* Device) for the treatment of opioid withdrawal. In individuals with AUD (*N* = 30), taVNS was associated with reductions in alcohol craving (*p* < 0.05) and increased parasympathetic tone (Treiber et al. 2024). Furthermore, a study evaluating taVNS for protracted alcohol withdrawal symptoms in 114 men (active *N* = 58, sham *N* = 56) reported improved depression symptoms and sleep quality as well as increased plasma BDNF levels (*p* < 0.001) following 4 weeks of treatment (Wang, Xu, et al. 2021). Moreover, our group's recent double‐blind, sham‐controlled clinical trial (active *N* = 9, sham *N* = 10) provides support that tcVNS can achieve reductions in adverse alcohol‐related consequences as well as psychosocial functional limitations in addition to improvements in depressive symptoms (Patient Health Questionnaire‐8: *p* = 0.04, Brief Inventory of Psychosocial Functioning: *p* = 0.01, etc.) (Klaming et al. 2025). Ongoing clinical trials are expected to provide further insight into the efficacy of VNS as a treatment for substance use disorder and the underlying neural and physiological mechanisms of action.

##### 5.2.4.3 Eating Disorder

Research into VNS for eating disorders (such as anorexia nervosa) is in the early stages. An open‐label pilot case series applied taVNS in 15 adult women with longstanding eating disorders (9 Anorexia Nervosa, 5 Bulimia Nervosa, 1 Binge Eating) and comorbid mood symptoms. Participants self‐administered taVNS for 4 h daily over 9 weeks. By the study's end, significant improvements in depressive and anxiety symptoms were noted and there were also modest increases in body mass index among underweight patients (an average 5%–11% BMI gain in those with anorexia/bulimia) (Melis et al. 2020). Given that patients with severe anorexia nervosa exhibit high rates of bradycardia (up to 36%) (Guinhut et al. 2021) and elevated vagal tone (30% higher than healthy subjects) (Kollai et al. 1994), it is notable that no VNS‐mediated averse cardiac outcomes were reported in this study (Melis et al. 2020). This absence of vagal stimulation‐related cardiac adverse events suggests that taVNS may influence disease severity through non‐cardiac pathways, potentially via modulation of reward circuitry.

##### 5.2.4.4 Obsessive‐Compulsive Disorder

A pilot study investigated the use of VNS in patients with treatment‐resistant obsessive‐compulsive disorder (OCD). In the study, seven patients with OCD received adjunctive VNS therapy alongside their existing medications. During the acute 12‐week phase, three of these patients exhibited a ≥ 25% reduction in Yale‐Brown Obsessive Compulsive Scale (Y‐BOCS) scores (George et al. 2008). More work is needed to determine if VNS will have any effect on OCD or OCD‐related disorders.

### 5.3 Inflammatory and Autoimmune Diseases

#### 5.3.1 Sepsis

The vagal anti‐inflammatory mechanism through the CAP pathway potently suppresses the cytokine surge characteristic of sepsis, which has been demonstrated in both animal and human models (Murray and Reardon 2018; Borovikova et al. 2000). Notably, clinical data from a RCT in 20 septic patients (active *N* = 10, sham *N* = 10) showed that daily tcVNS significantly lowered circulating proinflammatory TNF‐α and IL‐1β (*p* = 0.002) while boosting anti‐inflammatory IL‐10 and IL‐4 levels (*p* < 0.01) (Wu, Zhang, et al. 2023) after 7 days of treatment, which confirms that VNS can shift the balance of circulating cytokines toward an anti‐inflammatory profile in the acute sepsis conditions. However, human studies of vagal anti‐inflammatory effects showed mixed results, as demonstrated by a controlled study where transvenous VNS during experimental human endotoxemia failed to modulate plasma cytokine levels, leukocyte cytokine production capacity, or neutrophil phagocytosis (active *N* = 10, sham *N* = 20, *p* > 0.05) (Kox et al. 2015). This variability in human responses suggests that factors such as clinical context, stimulation parameters, timing of stimulation, and patient selection may be critical determinants of therapeutic efficacy.

Beyond direct cytokine effects, VNS helps rebalance autonomic outflow in septic shock, which can stabilize hemodynamics and protect organ function. Sepsis is often marked by sympathetic overdrive and vagal withdrawal, contributing to tachycardia, vasodilation, and arrhythmias. Vagal stimulation restores parasympathetic tone, countering sympathetic hyperactivity and improving indicators like HRV (which inversely correlates with inflammatory severity) and attenuating septic shock‐induced cardiac injury (Sloan et al. 2007; Yuchao et al. 2023). Another important aspect of VNS in critical illness is promoting a return to immune homeostasis after the initial hyperinflammatory phase. The HPA axis activation by VNS leads to a surge of endogenous cortisol. The vagus effectively triggers this neuroendocrine feedback loop, helping to globally tamp down inflammation and prevent it from overshooting (Bonaz et al. 2019). By engaging both the cholinergic reflex and HPA axis, VNS facilitates a coordinated resolution of systemic inflammation. In addition, VNS also prevents the sepsis‐induced suppression of cardiac mitochondrial Complex II and Complex IV respiratory activity and normalized oxygen consumption in a pig study (Kohoutova et al. 2019).

Notably, timely vagal stimulation in septic models not only blunts early cytokine storms but also avoids driving the immune system into prolonged suppression. A mouse study has observed that activating the vagal anti‐inflammatory pathway during early sepsis promotes faster immune recovery (e.g., restoration of normal white blood cell function), rather than causing an immune‐paralyzed state (Mac et al. 2025). This balanced immunomodulation is critical in sepsis and critical illness—it helps quell damaging inflammation while preserving the capacity to fight infection. However, it is notable that there are no controlled studies that compare the effects of early‐stage VNS sepsis treatment versus later‐stage sepsis VNS treatment; it is the author's opinion that this work must be completed to effectively treat sepsis and infection‐related inflammatory disorders such as acute respiratory distress syndrome (ARDS).

#### 5.3.2 Arthritis and Inflammatory Joint Disease

Chronic inflammatory arthritides such as RA and psoriatic arthritis are driven by excessive cytokine release and sustained synovial inflammation. VNS engages neuroimmune circuits that can counteract this pathological immune response through the CAP pathway. Proinflammatory cytokines such as TNF‐α and IL‐1 are central mediators of arthritis pathogenesis, driving synovial tissue inflammation and joint damage (Dayer 2002). Alongside cytokine changes, patients with arthritis receiving VNS have shown a reduction in inflammatory biomarkers like C‐reactive protein (CRP) (Marsal et al. 2021). In RA patients (*N* = 17), iVNS was shown to inhibit peripheral cytokine production and significantly reduce TNF‐α, IL‐6, and IL‐1β levels, with associated decreases in swollen joint counts and disease activity scores. Therapeutic benefits are reversibly linked to device activation, as TNF‐α and CRP rebounded when VNS was discontinued and decreased again upon reactivation, providing direct evidence of the anti‐inflammatory effect of VNS (Koopman et al. 2016). However, clinical outcomes have been mixed. A recent RCT of 113 RA patients found that 12‐week taVNS failed to demonstrate significant clinical benefit compared to sham treatment including American College of Rheumatology criteria and CRP levels (*p* > 0.05) (Baker et al. 2023). Notably, in July 2025, the FDA approved an iVNS technology by SetPoint Medical for treating RA, after its demonstration of safety and feasibility from the RESET‐RA study (Peterson et al. 2024). More recently, SecondWave Systems Inc. has shown initial pilot data for splenic ultrasound treatments in reducing RA Disease Activity Score‐28 for RA with CRP (DAS28‐CRP) scores in 10/13 patients treated (*p* < 0.003) unpublished data; the device is now in multi‐site randomized double blinded controlled phase 1 clinical trials to treat RA.

VNS has also shown some promising therapeutic effects in other inflammatory joint diseases. In a proof‐of‐concept study, psoriatic arthritis patients (*N* = 20) receiving 5 days of tcVNS showed a significant reduction in disease activity scores (*p* = 0.002) and CRP levels by 20% (*p* = 0.03). In the same study, patients with ankylosing spondylitis (*N* = 17) exhibited significant decreases in IFN‐γ (*p* = 0.02), IL‐8 (*p* = 0.03), and IL‐10 (*p* = 0.008) (Brock et al. 2021), with improved cardiac vagal tone observed in both disease groups (*p* < 0.05) (Brock et al. 2021).

#### 5.3.3 Inflammatory Bowel Disease

Similar to other inflammatory conditions, VNS demonstrates a targeted anti‐inflammatory mechanism through the CAP. Specifically, in animal models, VNS selectively reduces proinflammatory cytokine production in the gut mucosa, effectively mitigating inflammatory responses while preserving anti‐inflammatory cytokines such as IL‐10 (Fornaro et al. 2022). Studies in Crohn's disease patients (*N* = 7) have directly shown this cytokine modulation. After months of implanted VNS therapy, patients exhibited marked reductions in key inflammatory cytokines in plasma and gut tissue (Sinniger et al. 2020). At the cellular level, VNS also limits immune cell trafficking to the gut. In rodent colitis models, vagal stimulation led to fewer monocyte and neutrophil infiltrates in the colon and lowered inflammatory gene expression in macrophages (Meroni et al. 2021).

Beyond cytokines, VNS exerts beneficial effects on the enteric nervous system and the integrity of the intestinal barrier. Enhancing vagal activity can normalize disordered gut motility and secretion that often accompany IBD. Patients with Crohn's and ulcerative colitis commonly have autonomic imbalances, which can exacerbate pain and dysmotility, and VNS helps restore vagal signaling in the gut (Fornaro et al. 2022). Increased vagal output can influence the enteric circuit to promote mucosal secretion and microvasculature function, potentially mitigating motility disorder, visceral hypersensitivity, and dysbiosis (Bonaz et al. 2019).

Importantly, VNS helps strengthen the gut barrier, which is often compromised by IBD. The vagal anti‐inflammatory pathway has direct effects on the intestinal epithelium and tight junction integrity. Preclinical studies have shown that vagal stimulation stimulates intestinal epithelial cell regeneration and reduces apoptosis during colitis. In a mouse model of ulcerative colitis, VNS increased proliferative healing of the colonic epithelium and led to a stronger mucus layer, resulting in fewer and milder ulcers (Meroni et al. 2021). Consistently, VNS reduced the rise of intestinal permeability that accompanies inflammation, with mast cell degranulation potentially contributing to the effect (Wang et al. 2024). In healthy volunteers, taVNS was found to significantly protect against acute stress‐induced “leaky bowel,” reducing the increase in intestinal permeability by about half compared to sham stimulation (Mogilevski et al. 2022). However, the evidence of VNS's effect on the HPA axis for the murine urine colitis model is limited (Youssef et al. 2024).

Additionally, a distinct vagal anti‐inflammatory pathway has been identified in the intestine that operates independently of the spleen. In a murine study, Matteolli et al. demonstrated that VNS attenuates surgery‐induced intestinal inflammation and improves intestinal transit through direct interactions between cholinergic myenteric neurons and muscularis resident macrophages expressing α7nAChR, suggesting these intestinal macrophages are the ultimate cellular targets of the GI CAP, independent of the spleen (Matteoli et al. 2014). Beyond traditional VNS approaches, emerging noninvasive neuromodulation strategies are also being investigated for IBD in preclinical models. In a rat model of colitis, noninvasive peripheral FUS of celiac plexus improved stool consistency, reduced gross bleeding, and preserved colon length compared to untreated controls, with associated reduction in inflammatory cytokines including IL‐1β, IL‐6, and TNF‐α (Akhtar et al. 2021).

In an initial open‐label trial of iVNS for Crohn's disease, five of nine patients achieved combined clinical, biological, and endoscopic remission, demonstrating significant improvements in disease activity indices (Sinniger et al. 2020). A subsequent larger prospective study of 16 drug‐refractory patients treated with iVNS (4 patients receiving concomitant biologics) confirmed these findings, showing a clinically meaningful decrease in Crohn's disease activity index (*p* = 0.003), in faecal calprotectin (*p* = 0.015), alongside 46%–52% reduction in serum TNF‐α and IFN‐γ after 16 weeks of treatment (D'Haens et al. 2023). Additionally, the transcutaneous approach also shows some promising results. A prospective trial in 22 pediatric IBD patients (12 Crohn's disease, 10 ulcerative colitis) demonstrated that taVNS significantly reduced intestinal inflammation, with 64.7% of evaluable participants achieving at least a 50% reduction in fecal calprotectin by week 16 (Sahn et al. 2023). A systematic review investigated the beneficial effect of iVNS and taVNS for IBD and concluded that VNS exhibited trends toward reducing inflammation and mitigating sympathetic dominance without long‐term adverse effects (Pikov 2023). Another mechanism review by Payne et al. also suggested that VNS therapy is more appropriate for patients with mild or less‐advanced Crohn's, though the mechanisms underlying treatment non‐responsiveness in certain patients remain unclear (Payne et al. 2019).

#### 5.3.4 Other Inflammatory Diseases

##### 5.3.4.1 Systemic Lupus Erythematosus

Human studies in systemic lupus erythematosus (SLE) suggest that VNS can modulate immune function and alleviate symptoms. In a pilot sham‐controlled trial of taVNS in SLE patients (*N* = 18) with chronic pain, 4 days of stimulation significantly reduced musculoskeletal pain and fatigue compared to sham. Clinical improvements (pain reduction and fatigue improvement) correlated with the amount of vagal stimulation delivered (pain: *p* = 0.05, fatigue: *p* = 0.003). Mechanistically, taVNS led to a marked drop in plasma substance P (a proinflammatory neuropeptide involved in pain signaling), although no immediate significant changes were detected in broader inflammatory cytokines or kynurenine metabolites (Aranow et al. 2021).

##### 5.3.4.2 Systemic Sclerosis

In systemic sclerosis (SSc), a connective tissue disease with prominent inflammation, fibrosis, and pain, VNS has shown immunomodulatory benefits in humans. A recent crossover trial in SSc patients (*N* = 21) applied tcVNS for 4 weeks as an add‐on therapy for chronic pain. VNS was associated with a significant reduction in self‐reported pain (*p* = 0.002). Importantly, serum IL‐6 levels declined with active VNS compared to the sham stimulation period (*p* = 0.029) (Bellocchi et al. 2023).

##### 5.3.4.3 Primary Sjögren's Syndrome

Severe fatigue and systemic immune activation in primary Sjögren's syndrome (pSS) have prompted exploratory VNS studies focusing on mechanistic outcomes. Tarn et al. carried out an open‐label trial; 15 female patients with pSS self‐administered tcVNS twice daily for 4 weeks. The study showed VNS‐related reduction in LPS‐stimulated peripheral blood (TNF‐α: *p* = 0.001, IL‐1β: *p* = 0.005) after VNS treatment (Tarn et al. 2019). Further, VNS was associated with significant improvements in fatigue (*p* = 0.003) and daytime sleepiness (*p* = 0.02) over the treatment period (Tarn et al. 2019). Further, VNS was associated with significant improvements in fatigue and daytime sleepiness over the treatment period (Tarn et al. 2019). A subsequent double‐blind trial in pSS (active *N* = 20, sham *N* = 20) reinforced these findings (Tarn et al. 2023). Patients receiving active VNS showed greater reductions in both physical and mental fatigue scores (*p* < 0.05) by day 54 compared to the sham group. Interestingly, acute VNS was found to modulate EEG alpha rhythms, and the degree of frontal and central alpha reactivity correlated with improvements in fatigue (frontal: *r* = −0.91, *p* < 0.01, central: *r* = −0.71, *p* = 0.02) (Tarn et al. 2023). This suggests that beyond suppressing peripheral cytokine release, VNS may enhance central autonomic networks and relieve fatigue, while this change may also, in part, be due to the VNS anti‐inflammatory effects.

#### 5.3.5 Mixed Evidence

While multiple studies have demonstrated promising anti‐inflammatory effects of VNS across various conditions, a recent systematic review of 36 studies with 1335 participants found no consistent evidence that VNS reduces inflammatory cytokines in humans (Schiweck et al. 2024). The meta‐analysis revealed no statistically significant effects of VNS on key inflammatory cytokines, including TNF‐α, IL‐6, IL‐1β, and IL‐10, with substantial heterogeneity across studies (*I* (Austelle et al. 2024) values often > 75%), reflecting significant variation in patient populations, stimulation parameters, and clinical contexts. Several factors may contribute to this result. Most studies have small sample sizes (5–62 participants per group) and poor to fair quality study design, with many lacking appropriate controls. Importantly, most human studies focused on chronic, treatment‐resistant conditions rather than acute inflammatory events, a key difference from successful animal studies that typically administered VNS prior to or during inflammatory onset. The review demonstrated that VNS appeared more effective in acute rather than chronic inflammatory responses. This finding explains why the clinical successes observed in sepsis and perioperative settings, where VNS intervention coincides with acute inflammatory activation, may not translate to chronic inflammatory diseases (such as long‐Covid (Verbanck et al. 2021; Pfoser‐Poschacher et al. 2025) and reduction of inflammation caused by diabetes (Okdahl et al. 2024)). *Therefore, the timing of intervention appears crucial for therapeutic efficacy, while animal studies demonstrate robust anti‐inflammatory effects when VNS is applied before or during acute inflammatory challenges, human studies mainly investigate VNS in chronic conditions where the immune system may be less responsive to the therapy*.

In addition, results from systematic reviews collect data from many potential VNS applications and disease targets. It should be noted that the mechanism of action of VNS (e.g., activation of either the splenic or gastrointestinal CAP) must also be carefully considered. These neuroimmune pathways have been found to modulate a subset of cytokines (including TNF‐α, IL‐6, and IL‐1), and these may not be the important inflammatory mediators for all current applications/disease targets. In addition, recent evidence suggests that neuroimmune pathways (such as CAP) undergo endogenous alterations during chronic inflammatory events, and constitutive activation may decrease the effectiveness of the protective anti‐inflammatory effects (Rana et al. 2018; Ahmed et al. 2022). This provides further evidence that the timing of stimulation during disease progression may be essential, and that other methods of inhibiting activation within the pathways or mediating adverse compensatory effects of constitutive endogenous activation may be necessary in our approach to treating chronic diseases.

### 5.4 Cardiovascular Diseases

#### 5.4.1 Atrial Fibrillation

Atrial fibrillation (AF) initiation and maintenance are strongly influenced by autonomic tone. Heightened sympathetic activity is pro‐arrhythmic, while parasympathetic influence can either suppress or promote AF depending on stimulation intensity. High‐level VNS, delivered above the bradycardia threshold, activates slow B‐fibers and C‐fibers. During high‐level VNS, ACh and vasoactive intestinal peptides are released. ACh binds to M2 muscarinic receptors, leading to inhibition of the G protein α‐subunit and decreased levels of cyclic adenosine monophosphate and protein kinase. In addition, ACh activates outward potassium current (IKACh), resulting in membrane hyperpolarization and shortened repolarization (Silvani et al. 2016). In contrast, low‐level VNS is delivered below the bradycardia threshold and selectively activates larger A‐fibers and fast B‐fibers, avoiding excessive direct parasympathetic effects on the sinus node (Stavrakis et al. 2017; Park et al. 2012; Kharbanda et al. 2022). This therapeutic VNS is typically applied in a continuous low‐level manner, which can prolong the effective refractory period and slow the AF reentrant circuit without inducing the heterogeneity seen with abrupt vagal bursts (Rast et al. 2024). In canine models, continuous low‐level VNS was shown to suppress stellate ganglion activity and prevent paroxysmal AF episodes by dampening cardiac sympathetic drive (Shen et al. 2011; Park et al. 2012). Notably, this “vagal paradox” (whereby intense discharge can trigger AF, yet low‐level vagal activation is protective) has been shown in multiple experimental studies (Rast et al. 2024). Ludwig et al. recently showed that the cervical vagal nerve complex harbors multiple tyrosine hydroxylase‐positive staining fibers, indicating that sympathetic chain fibers cannot be avoided when stimulation is carried out in the cervical vagus (Deshmukh et al. 2025). Low‐level VNS protective effect likely results from multiple mechanisms, including prolongation of atrial refractoriness, suppression of ectopic triggers, reduced AF duration and burden, and enhanced HRV (Rast et al. 2024). The clinical efficacy of low‐level vagal stimulation has been demonstrated in humans, with taVNS significantly reducing the occurrence of postoperative AF compared to the control group (Stavrakis et al. 2017; Andreas et al. 2019).

In addition, AF is not only a cardiovascular disorder but also involves an inflammatory and structural substrate. Stavrakis et al. randomized 54 patients undergoing cardiac surgery to low‐level iVNS (*N* = 26) or sham control (*N* = 28), finding the therapy reduced post‐operative AF from 36% vs. 12% (*p* = 0.027) and significantly suppressed inflammatory markers, including TNF‐α (time by group interaction *p* = 0.02) and IL‐6 (group effect *p* = 0.03) at 72 h post‐operatively (Stavrakis et al. 2017). Similarly, Andreas et al. randomized 40 cardiac surgery patients to taVNS (*N* = 20) or sham (*N* = 20) for up to 14 days, demonstrating reduced post‐operative AF from 55% vs. 20% (*p* = 0.022), though no significant effects on inflammatory markers (i.e., CRP and IL‐6) were observed at day 7 (Andreas et al. 2019). Extending beyond post‐operative settings to chronic treatment, the TREAT‐AF trial randomized 53 patients with paroxysmal AF to daily 1‐h taVNS (*N* = 27) or sham (*N* = 26) for 6 months, showing 85% lower median AF burden (*p* = 0.011) and significant TNF‐α suppression by 23% (*p* = 0.0093) (Stavrakis et al. 2020). Therefore, by effectively modulating both cardiac rhythm and inflammatory responses, VNS emerges as a promising therapeutic strategy for AF.

#### 5.4.2 Myocardial Infarction and Reperfusion Injury

Acute MI is a leading cause of death and a major contributor to cardiovascular disease mortality (Mechanic et al. 2017). While the expeditious restoration of coronary perfusion represents an important therapeutic strategy for reducing myocardial ischemia injury, limiting infarct size, and improving ventricular dysfunction, the reperfusion process itself paradoxically induces additional myocardial damage through I/R injury (Chen et al. 2020). This pathophysiological dichotomy presents a significant therapeutic challenge, wherein the essential intervention of reperfusion simultaneously triggers molecular cascades that can potentially exacerbate tissue damage and compromise clinical outcomes (Chen et al. 2020). In this context, cumulative evidence shows that VNS exerts cardioprotective effects against myocardial I/R injury through multiple pathways and mechanisms.

Myocardial I/R injury may cause cardiac autonomic nervous system imbalance with reduced cardiac vagal activity and over‐activated sympathetic tone (Jardine et al. 2005). Studies have shown that activation of the sympathetic nervous system is a key contributor to inflammatory reactions and infarct size (Pongratz and Straub 2014; Karlsberg et al. 1979). Therefore, the neuromodulatory effects of VNS, characterized by autonomic balance, play a cardioprotective role in minimizing acute myocardial I/R injury (Uitterdijk et al. 2015). VNS has demonstrated significant efficacy in reducing myocardial infarct size across various animal species, including mice (Katare et al. 2009), swine (Shinlapawittayatorn et al. 2013), and canine (Chen, Zhou, et al. 2016). The cardioprotective effect of VNS on infarct size appears to be independent of its bradycardia effects (Chen, Zhou, et al. 2016; Katare et al. 2009).

Inflammation is also one of the major pathophysiological mechanisms involved in the pathogenesis of myocardial I/R injury (Ottani et al. 2010). Myocardial infarction triggers a complex inflammatory response that is essential for healing and scar formation. When coronary artery occlusion occurs, the reduced blood flow leads to myocardial necrosis, which activates complement and generates free radicals, initiating a cytokine cascade starting with TNF‐α release. If reperfusion occurs, it intensifies the inflammatory reaction, with IL‐8 and C5a playing crucial roles in recruiting neutrophils to the damaged area (Frangogiannis et al. 2002). Therefore, inflammation treatment may be a potential therapeutic strategy for improving clinical outcomes in patients with MI and myocardial I/R injury. Given inflammation's central role in this process, the anti‐inflammatory interventions of VNS emerge as a promising therapeutic strategy for improving clinical outcomes in patients with MI and myocardial I/R injury (Yu, Huang, et al. 2017).

Mitochondria also play a crucial role in MI as they are key regulators of cardiomyocyte contraction, metabolism, inflammation, and cell death. During acute ischemia, cellular hypoxemia switches metabolism from aerobic respiration to anaerobic glycolysis, leading to lactate production, decreased cellular pH, and calcium overload in both cytoplasm and mitochondria. This results in arrested oxidative phosphorylation and ATP depletion, causing decreased myocardial contractility. Upon reperfusion, paradoxically, the reintroduction of oxygen leads to ROS release and promotes further mitochondrial calcium overload, which can trigger the opening of mitochondrial permeability transition pores and cell death despite blood flow restoration (Zhou et al. 2021). VNS has been shown to have multiple cardioprotective effects in pre‐clinical models of I/R injury. VNS decreases cardiac mitochondrial ROS production, prevents mitochondrial membrane depolarization and swelling, improves mitochondrial biogenesis and dynamics (increased mitochondrial fusion and decreased mitochondrial fission), shifts cardiac fatty acid metabolism toward beta‐oxidation, increases levels of cardiac mitochondrial complexes and inhibits the opening of mitochondrial permeability transition in pig (Shinlapawittayatorn et al. 2013; Nuntaphum et al. 2018) and rat (Katare et al. 2009) models. Concurrently, VNS activates pro‐survival cellular pathways in the heart, akin to ischemic preconditioning, by triggering the PI3K‐Akt and JAK2/STAT3 signaling mechanisms that inhibit cell death and protect mitochondrial function (Kakinuma et al. 2005; Uemura et al. 2010; Mioni et al. 2005). These molecular processes reduce cardiomyocyte apoptosis and necrosis, ultimately helping the heart tissue better tolerate ischemic injury and preserve tissue integrity during reperfusion (Xu et al. 2023; Giannino et al. 2024).

Through the above mechanisms, VNS significantly limits infarct size in acute MI models. Meta‐analyses of preclinical studies indicate that VNS can reduce infarcted myocardial area by ~25%–30% compared to controls (Xu et al. 2023). Importantly, VNS also influences the longer‐term remodeling of the heart after an MI. By curbing initial injury and inflammation, VNS leads to smaller scar formation and more viable myocardium, which translates to improved left ventricular function over time (Xu et al. 2023; Giannino et al. 2024). Vagal stimulation has also been shown to preserve microvascular and endothelial function in the infarct border zone, in part by increasing endothelial NO availability and vagal co‐transmitters that support perfusion (Giannino et al. 2024).

In a clinical setting, an RCT of taVNS in ST‐elevation myocardial infarction (STEMI) patients (active *N* = 54, sham *N* = 55) demonstrated that taVNS applied during and after percutaneous coronary intervention significantly reduced hospital mortality (0% vs. 9%, *p* = 0.025) and cardiogenic shock (5.6% vs. 18%, *p* = 0.033) (Kruchinova et al. 2025). Similarly, one study investigated the effect of taVNS in STEMI (active *N* = 47, sham *N* = 48) and found that taVNS applied from catheterization room arrival through 2 h post‐reperfusion resulted in reduced reperfusion‐associated ventricular arrhythmias (*p* < 0.05), serum myocardial injury marker levels (CK‐MB and NT‐proBNP: *p* < 0.05), inflammatory markers (IL‐6, IL‐1β, HMGB1, TNF‐α: all *p* < 0.05) and improved echocardiographic function (LV ejection fraction [LVEF]: *p* = 0.01) compared to sham (Yu, Huang, et al. 2017). These data suggest that taVNS may reduce adverse outcomes following STEMI, possibly mediated by a change in proinflammatory states. However, further clinical studies are needed to validate these findings.

#### 5.4.3 Heart Failure

Heart failure occurs both in the presence of preserved (HFpEF) and reduced (HFrEF) LV systolic function, and is characterized by autonomic imbalance, neurohormonal activation, and chronic inflammation (Bonow et al. 2011). Augmenting parasympathetic tone and reducing sympathetic drive is a core mechanism of VNS in heart failure and underlies the mechanism of core heart failure therapeutics such as beta‐blockers. Chronic HF is marked by excessive sympathetic activation and vagal withdrawal, which correlate with worse outcomes (Pahuja et al. 2023). Clinical studies in HFrEF have demonstrated increased HRV and improved baroreflex sensitivity with VNS, indicating partial reversal of autonomic remodeling (Pahuja et al. 2023). In addition, both HFrEF and HFpEF patients exhibit elevated inflammatory biomarkers, and VNS has demonstrated the ability to reduce circulating cytokine levels through the CAP pathway (Stavrakis et al. 2022).

Beyond autonomic and immune modulation, VNS exerts a direct cardioprotective effect that can improve myocardial function and remodeling. Vagal efferent stimulation releases ACh onto the heart, which slows the sinus rate and AV node conduction. The resulting reduction in HR and prolongation of diastole can enhance ventricular filling and reduce myocardial oxygen demand, benefiting patients with high HRs or ischemic stress (Lund et al. 1986). VNS has also been shown to enhance intrinsic myocardial contractility via secondary messengers: for example, chronic VNS augments NO production in cardiac myocytes, which in turn may improve calcium handling and inotropy (Brack et al. 2007). On a structural level, VNS favorably influences cardiac remodeling. Studies in post‐infarction HF models found that VNS attenuates left ventricular dilation, myocyte hypertrophy, and interstitial fibrosis. Anti‐apoptotic and anti‐oxidative stress pathways are activated by VNS, limiting myocyte cell death and oxidative injury in the failing heart (Bazoukis et al. 2023; Chen, Zhou, et al. 2016).

HF is also perpetuated by neurohormonal overactivity, notably, elevated circulating catecholamines and RAAS activity (Bonow et al. 2011). VNS helps normalize neurohormonal levels through its central and reflex effects on autonomic outflow. Preclinical HF studies indicate that interrupting vagal signaling raises plasma renin, whereas VNS acutely lowers angiotensin II levels (Pahuja et al. 2023; Wu, Liao, et al. 2023). Substantial clinical evidence confirms that medications suppressing sympathetic nervous system activity, with beta‐adrenergic receptor blockers being the prime example, serve as effective long‐term treatments for heart failure patients (Sabbah et al. 2011). Therefore, by restraining the RAAS system, VNS provides a pharmacotherapeutic alternative approach for HF treatment.

However, despite significant pre‐clinical evidence, VNS in heart failure has failed to consistently translate into improved outcomes in clinical trials. An early open‐label single‐arm uncontrolled trial, the ANTHEM‐HF study, evaluated the effect of VNS in patients with New York Heart Association (NYHA) functional class II–III HFrEF (Left VNS: *N* = 31, Right VNS: *N* = 29). Combined analysis at 6 months showed that iVNS resulted in improvements in echocardiographic parameters (e.g., LVEF, LV end‐systolic volume), SDNN, NYHA function class (77% of patients), and 6‐min walk distance (all *p* < 0.01) (Premchand et al. 2014). The ANTHEM‐HF extension study showed that these improvements were sustained at 12 months with continued statistical significance (all *p* < 0.01) and minimal change from the 6‐month endpoint, indicating durable therapeutic effects (Premchand et al. 2016). Unfortunately, the follow‐up RCT (ANTHEM‐HFrEF), which randomized 533 patients to VNS or medical therapy, was terminated early by the sponsor for undisclosed reasons, and the incomplete results have not been published (Konstam et al. 2024). The NECTAR‐HF study was a RCT that randomized patients with HFrEF receiving 6‐month therapy of cervical‐(*N* = 63) or sham‐iVNS (*N* = 32) (Zannad et al. 2015). There were no significant differences in the primary outcome of LV‐end systolic volume, and other secondary outcomes (echocardiographic parameters, serum biomarkers) (*p* > 0.05). It turns out that 88% of patients did not have VNS‐associated heart rate responses, suggesting inadequate activation of vagal fibers in an exploratory analysis of the extended follow‐up (De Ferrari et al. 2017). Subsequently, the INOVATE‐HF study randomized 707 patients with NYHA functional class III HFrEF to iVNS or medical therapy alone (active *N* = 436, control *N* = 271) (Gold et al. 2016). Similarly to NECTAR‐HF, VNS therapy did not reduce the primary composite endpoint of all‐cause mortality or first heart failure event (30.3% vs. 25.8%, *p* = 0.37). Additionally, there were no significant differences in all‐cause mortality (annual rate 9.3% vs. 7.1%, *p* = 0.19); however, there were significant increases in NYHA functional class and 6‐min walking distances (*p* < 0.05) (Zannad et al. 2015). Together, these clinical data suggest that VNS may only improve quality of life but does not significantly enhance clinical outcomes in patients with HF, underscoring the need for additional research to clarify its therapeutic potential. Importantly, Ardell et al. proposed the concept of “neural fulcrum,” which refers to the optimal balance point (based upon fiber recruitment modeled by strength‐duration curves, e.g., see Figure 2) in VNS that maximizes heart rate modulation by large fiber recruitment while preserving autonomic reflexes, thereby not activating small fibers. The insignificant results of clinical trials such as ANTHEM‐HF, INNOVATE‐HF and NECTAR‐HF is primarily attributed to inappropriate electrical stimulation parameters, *which failed to effectively engage the neural fulcrum*, underscoring the crucial need for optimized electrode‐nerve interfaces, the refined stimulation protocol and careful patient selection (Ardell et al. 2017).

#### 5.4.4 Hypertension

VNS regulates hypertension by modulating autonomic circuits, resulting in autonomic balance, enhanced cardiac contractility, and reduced vascular resistance. VNS enhances baroreflex sensitivity, improving the autonomic system's responsiveness to blood pressure fluctuations. For example, in an acute RCT in healthy young men, a significant increase in baroreflex sensitivity (~24%) was observed, though no acute significant change in systolic and diastolic blood pressure (Antonino et al. 2017).

In chronic hypertension, early studies suggest repeated VNS over months may result in significant blood pressure reduction. A RCT of taVNS (*N* = 21) vs. sham (*N* = 19) stimulation in young adults with stage 1 hypertension showed a significant reduction in mean systolic blood pressure at 3 months. Diastolic blood pressure similarly decreased by about 5–7 mmHg with VNS, with little change in the control group. Notably, these benefits appeared within the first month of therapy and persisted through 3 months without significant changes in HR (Mbikyo et al. 2024). These data implicate chronic VNS as a promising neuromodulatory approach to treat hypertension.

### 5.5 Respiratory Diseases

#### 5.5.1 Asthma

Asthma is a chronic inflammatory airway disease characterized by bronchial hyperresponsiveness and airflow obstruction. The VN plays a central role in modulating smooth muscle tone, mucus secretion, and inflammatory responses. Efferent fibers innervate the airway smooth muscles, and their activation can lead to bronchoconstriction, a hallmark of asthma attacks (Kistemaker and Prakash 2019). High‐intensity VNS indiscriminately activates efferent vagal fibers and can provoke bronchospasm. In contrast, carefully tuned VNS at low amplitude engages bronchodilatory pathways without triggering overt cholinergic bronchoconstriction in pigs (Hoffmann et al. 2012). This effect appears to involve selective fiber recruitment: low‐level stimulation avoids activating the efferent vagal neurons that cause bronchoconstriction or bradycardia, instead recruiting fibers that trigger airway relaxation (Hoffmann et al. 2012). Another proposed mechanism is activation of vagal inhibitory non‐adrenergic, non‐cholinergic pathways that release relaxing neurotransmitters in the airways (Hoffmann et al. 2012; Mazzone and Canning 2002). Additionally, vagal afferents mediate protective reflexes such as cough, mucus secretion, and bronchoconstriction in response to irritants. In asthma, these sensory pathways are often hyperactive, leading to exaggerated cough and bronchospastic reflex (Kistemaker and Prakash 2019). Stimulating vagal afferent fibers can alter brainstem reflex circuits and reset cough and bronchoconstrictor reflex thresholds. Human trials have shown that auricular VNS can desensitize cough reflex sensitivity with specific stimulation parameters (Ng et al. 2024). By modulating bronchodilatory and cough reflexes in asthma with careful stimulation parameter design, VNS may reduce the propensity for hyperactive airway and hypersensitive reflex cough.

Beyond neural reflexes, VNS exerts a potent anti‐inflammatory effect in asthma, which is fundamentally an inflammatory airway disease. Preclinical studies indicate that engaging this pathway can suppress allergic inflammation in the lungs. In a house dust mite‐induced asthma model, direct VNS markedly attenuated airway inflammation and hyperactivity (Sévoz‐Couche et al. 2024). VNS also prevented goblet cell hyperplasia and collagen deposition in the airway, signs of suppressed chronic inflammation and remodeling (Sévoz‐Couche et al. 2024). Preliminary clinical studies have shown encouraging results. In one prospective trial, paVNS was utilized in patients with severe asthma exacerbation unresponsive to standard treatment. Patients experienced prompt bronchodilation and improved work‐of‐breathing and dyspnea scores after a 1‐h stimulation session (Miner et al. 2012). The *GammaCore* tcVNS has received FDA emergency use authorization for the acute treatment of asthma exacerbations in COVID‐19 patients and a CE mark for the treatment and prevention of symptoms of reactive airway disease, which includes asthma, bronchoconstriction, exercise‐induced bronchospasm, and chronic obstructive pulmonary disease (Staats et al. 2020), showing the technique is being accepted as a potential therapeutic intervention for respiratory conditions.

#### 5.5.2 Acute Respiratory Distress Syndrome

ARDS is a life‐threatening condition characterized by rapid‐onset respiratory failure due to widespread inflammation and fluid accumulation in the lungs. This leads to severe hypoxemia and impaired gas exchange, often necessitating intensive care and mechanical ventilation. COVID‐19‐associated ARDS (CARDS) is a severe complication of SARS‐CoV‐2 infection, characterized by diffuse alveolar damage, pulmonary inflammation, and microvascular thrombosis. The pathophysiology of CARDS involves a hyperinflammatory response, often referred to as a “cytokine storm,” which contributes to the severity of lung injury and increases the risk of mortality. In models of rat ARDS, VNS dramatically reduced levels of TNF‐α and IL‐1β while increasing the anti‐inflammatory cytokine IL‐10 through the CAP pathway (Li et al. 2021). This vagal CAP mechanism curbs the “cytokine storm” without causing global immunosuppression, thereby limiting inflammation‐induced lung injury (Kaniusas et al. 2020). Moreover, the VN influences the lung's local immune environment through both direct innervation and reflex pathways. VNS has been shown to shift pulmonary immune responses toward an anti‐inflammatory profile, promoting M2 macrophage polarization over proinflammatory M1 in the lungs. It also inhibits inflammatory cell death pathways (e.g., reducing Caspase‐1‐mediated pyroptosis in alveolar cells) via vagal signaling (Li et al. 2021). These neuroimmune effects can preserve alveolar‐capillary integrity and improve lung function during ARDS.

Clinical evidence from a systematic review of four RCTs (*N* = 180 in total) shows that VNS significantly increases IL‐10 levels, though effects on CRP, IL‐6, and cortisol remain inconclusive (Taha et al. 2024). However, one of the RCTs with 52 participants (active *N* = 26, sham *N* = 26) found a significant reduction in inflammatory biomarkers, including CRP, IL‐6, and cortisol (*p* < 0.05) in the treatment group, though no significant difference for the VNS effect on HRV in both active and sham after 7 days of treatment (*p* > 0.05) (Corrêa et al. 2022). In another study investigating the effect of VNS on severe COVID‐19 patients, the VNS group had a lower incidence of deterioration: fewer patients progressed to require mechanical ventilation than in standard care, and none in the VNS group required extracorporeal membrane oxygenation. Though there are only six patients in each group, the trend suggests VNS may help prevent the escalation of respiratory support in ARDS and improve patients' outcomes (Seitz et al. 2023).

### 5.6 Gastrointestinal and Metabolic Disorders

#### 5.6.1 Obesity

VNS engages the gut‐brain axis to influence hunger and satiety signals. Vagal afferent fibers carry sensory feedback from the stomach and intestine (e.g., stretch and nutrient‐related signals) to the NTS, which in turn connects to hypothalamic appetite centers. By electrically activating vagal pathways, VNS can potentiate these satiety circuits; studies have observed that VNS enhances feelings of fullness and suppresses food intake, effectively reducing ghrelin and appetite (Loper et al. 2021; Kozorosky et al. 2022). It reduces food intake by delaying gastric emptying and increasing gastric volume (Dai et al. 2020). In addition, VNS increases energy expenditure via activation of BAT. In a clinical study on epilepsy patients with implanted cervical VNS devices, active stimulation acutely raised resting metabolic rate and glucose uptake in BAT, with a strong correlation between increased energy expenditure and BAT activity (Vijgen et al. 2013). In essence, by engaging the hypothalamic‐brainstem circuits. VNS addresses both sides of the energy homeostasis equation: it curbs appetite via enhanced satiety signaling and simultaneously enhances energy utilization through metabolic activation.

Low‐frequency pulses of iVNS have led to weight loss in patients treated for epilepsy (Vijgen et al. 2013). Importantly, vBloc therapy using high‐frequency alternating current to inhibit vagal signaling has also shown promising results in obesity control (see Section 4.7.1). Two meta‐analyses reviewed VNS approaches for obesity management (Fadel et al. 2023; Hua et al. 2024). Fadel et al. reviewed 15 studies involving 1447 patients and found that VNS therapy, including vBloc, VNS, and paVNS, achieved statistically significant reduction in excess weight loss (mean difference: 17.19%, *p* < 0.001) and BMI (mean difference: 2.24 kg/m (Austelle et al. 2024), *p* = 0.016) compared to controls, with vBloc representing the most extensively studied modality (8 out of 15) (Fadel et al. 2023). In contrast, a separate meta‐analysis of auricular stimulation by Hua et al. examined 15 trials with 1333 patients and found statistically significant but clinically modest reduction in BMI (0.38 kg/m (Austelle et al. 2024), *p* < 0.0001) and body weight (0.66 kg, *p* = 0.005), falling short of the 5% total weight loss threshold considered clinically meaningful for obesity treatment (Hua et al. 2024). Therefore, vBloc therapy may be more effective in controlling obesity.

Notably, the vBloc EMPOWER RCT study involving 294 participants (active *N* = 192, control *N* = 102) did not achieve its primary efficacy endpoint at 12 months, with excess weight loss nearly identical between active (17% ± 2%) and control (16% ± 2%) groups (*p* = not significant) (Sarr et al. 2012). The active treatment delivered high‐frequency blocking (5 kHz, mode 6 mA) while the control group was treated with lower stimulation parameters (1 kHz and 40 Hz, ≤ 3 mA) intended as safety checks. However, subjects with high‐device adherence (≥ 12 h daily) demonstrated a substantial 30% ± 4% (treatment) versus 22% ± 8% (control) excess weight loss, with a significant linear correlation between usage hours and weight reduction (*p* < 0.001). The authors suggested that minimal electrical stimulation delivered to the control group may also be effective and confound the result. In January 2015, the FDA approved the MAESTRO Rechargeable system, a vBloc therapy, for treating obesity, though the device is discontinued in the market since 2022.

#### 5.6.2 Diabetes and Glucose Regulation

Vagal stimulation directly modulates pancreatic islet function. In an experimental study on anesthetized rats, researchers discovered that afferent VNS caused a strong and sustained increase in blood glucose concentration without stimulating insulin secretion, suggesting an inhibition of insulin release. In contrast, efferent VNS significantly raised insulin levels over 120 min of stimulation with only a temporary increase in glucose. The study highlights the differential effects of afferent and efferent vagal nerve stimulation on glucose metabolism, potentially offering insights into future therapeutic approaches for metabolic disorders like type II diabetes (Meyers et al. 2016; Li et al. 2014).

The VN also exerts important control over hepatic glucose metabolism. Cholinergic vagal fibers innervating the liver bind to hepatocyte muscarinic receptors, triggering intracellular pathways that promote glycogen synthesis and suppress gluconeogenic enzymes (Liu, Wu, et al. 2024). In hyperglycemic conditions, parasympathetic activation normally acts as a brake on hepatic glucose release (Meyers et al. 2016). This means vagal stimulation can shift the liver toward storing glucose as glycogen and lower the hepatic output of glucose into circulation. By curbing excessive hepatic glucose production (a common problem in type 2 diabetes), VNS may reduce fasting hyperglycemia and postprandial glucose peaks.

An important indirect mechanism by which VNS improves glucose regulation is through its anti‐inflammatory effect. Chronic low‐grade inflammation is a key contributor to insulin resistance in type 2 diabetes mellitus (T2DM) (Sorski and Gidron 2023). By reducing inflammation‐driven insulin resistance, VNS may improve insulin sensitivity, thereby aiding glucose uptake (Yu, He, et al. 2022). This anti‐inflammatory action of VNS is especially relevant since T2DM is now understood as an inflammatory condition as much as a metabolic one. However, the dedicated controlled human trials for VNS treatment on diabetes are still emerging. A recent RCT with both type 1 (*N* = 70) and type 2 (*N* = 75) diabetes treated with tcVNS (active *N* = 68, sham *N* = 77) found no overall improvement in glucose variability or time in range with either short‐term (1 week, 4 times daily) or long‐term (8 weeks, 2 times daily) treatment. However, subgroup analysis found that after 8 weeks, type 1 diabetes had an 11.6% reduction in coefficient of variation compared to sham (*p* = 0.009) while those with type 2 diabetes showed no significant changes, suggesting potential diabetes‐type‐specific benefits that require further investigation (Kufaishi et al. 2025).

#### 5.6.3 Gastrointestinal Disorders

VNS modulates upper gastrointestinal motor function through multiple vagal pathways that regulate gastric accommodation, pyloric sphincter tone, and peristalsis. VNS engages the brain‐gut axis to restore disordered gastric motor function. Preclinical studies have demonstrated that augmenting vagal activity can correct specific motor deficits. For example, in a rodent model of delayed gastric emptying, VNS increased the pyloric sphincter's opening and enhanced antral peristaltic contractions, resulting in accelerated gastric emptying; it highlights vagal stimulation's role in promoting pyloric relaxation and reducing outflow resistance (Hou et al. 2023; Lu et al. 2018). In healthy volunteers, a 4‐h session of taVNS significantly increased the gastric motility index, mainly by boosting the amplitude of antral contractions (Steidel et al. 2021).

VNS also appears to improve the stomach's reservoir function. Impaired fundic relaxation contributes to early satiety in functional dyspepsia and gastroparesis. By increasing vagal efferent drive to inhibitory enteric pathways, VNS may restore normal accommodation. A recent sham‐controlled trial in patients with functional dyspepsia showed that a single session of taVNS significantly enhanced gastric accommodation, as measured by increased maximum tolerable volume of the nutrient drink test (Zhu et al. 2021). Despite ingesting a larger meal volume, patients had lower postprandial fullness ratings with VNS, suggesting more effective accommodation and reduced visceral hypersensitivity. Notably, VNS in these patients also normalized gastric myoelectrical activity, increasing the percentage of normal slow‐wave rhythms and amplitude of gastric slow waves recorded by electrogastrography (Zhu et al. 2021). Notably, despite ingesting a larger meal volume, patients had lower postprandial fullness ratings with VNS, suggesting more effective accommodation and reduced visceral hypersensivity. Several large RCTs have demonstrated that VNS improves functional dyspepsia symptoms in adults (Shi et al. 2024) and adolescents (Kovacic et al. 2017). Mechanistically, a rat study demonstrated that taVNS improves gastric hypersensitivity via the vago‐vagal pathways by improving autonomic balance, ACh concentration in gastric tissue, suppressing inflammation, inhibiting the overactivated HPA axis, and improving impaired mucosal integrity (Hou et al. 2023).

Although mechanistic studies are still emerging, early clinical results align with these physiological benefits. A systematic review of seven RCTs with 644 patients demonstrates that VNS significantly improved GI disorder symptoms across functional dyspepsia, IBS and IBD (Veldman et al. 2025). In an open‐label pilot on idiopathic gastroparesis patients, 4 weeks of tcVNS led to symptomatic improvement (reduced nausea, bloating, and pain) (Gottfried‐Blackmore et al. 2020). Similarly, taVNS has been associated with relief in dyspeptic symptoms like early satiety and bloating in functional dyspepsia (Shi et al. 2024; Wu et al. 2021; Zhu et al. 2021). For example, Shi et al. explored taVNS with different stimulation frequency settings in patients with functional dyspepsia (25 Hz *N* = 99, 10 Hz *N* = 101, sham *N* = 100). They observed that both treatment groups had significantly higher response rates (81.2% vs. 75.9% vs. 47%, *p* < 0.001) and relief rates (85.1% vs. 80.8% vs. 67%, *p* < 0.05) than the sham group after 4 weeks of treatment (Shi et al. 2024). Notably, both taVNS and paVNS technologies have received FDA clearance in treating IBS (see Section 3.1), making regulatory recognition of this therapeutic approach and indicating a leading role of GI doctors in adopting VNS. While these trials are preliminary, they underscore that augmenting vagal signaling can favorably impact gastric sensorimotor functions.

### 5.7 Renal Diseases

#### 5.7.1 Acute Kidney Injury and Chronic Kidney Disease

AKI is a life‐threatening condition characterized by a sudden decline in renal function triggered by I/R injury, sepsis, nephrotoxic insults, with inflammation, oxidative stress, and apoptosis as key contributors to its pathogenesis. A central mechanism of renal protection is through activation of the CAP. VNS administered prior to renal I/R significantly attenuated AKI and decreased plasma cytokine levels (Inoue et al. 2016). Notably, this protective effect was abolished in animals lacking α7nAChR, or those that underwent splenectomy, underscoring the critical role of α7nAChR‐positive splenocytes in mediating VNS‐induced renal protection (Inoue et al. 2016). Further, VNS influences immune cell phenotypes within the kidney. Studies have demonstrated that VNS shifts macrophage from proinflammatory M1 phenotype to an anti‐inflammatory M2 phenotype, contributing to reduced renal inflammation and injury (Inoue et al. 2016). As mentioned in other inflammatory‐mediated disorders, the utility and or efficacy of VNS may be predicated on treating early in disease progression, as noted above, most positive pre‐clinical VNS effects are dependent on administration of VNS prior to the disease injury, that is, AKI in this case. Although further discussed in Section 6, it is the author's view that early identification of each disease state (AKI or others) by wearable noninvasive multi‐modal sensor modeling will allow for early and or instantaneous treatments, that is, autonomous closed loop systems, thereby allowing the clinician to fully leverage the enhanced efficacy of early VNS.

Oxidative stress is another critical factor in AKI pathogenesis, where excessive ROS production leads to substantial renal tissue damage. The transcription factor Nrf2 upregulates HO‐1 transcription and promotes potent antioxidative effects, which protect renal cells against oxidative stress and apoptosis by modulating the ROS cell death pathway. VNS has been demonstrated to effectively enhance the Nrf2/HO‐1 signaling axis, bolstering the kidney's endogenous antioxidant defense mechanism and mitigating oxidative damage (Deng et al. 2022; Wang et al. 2020). Further, excessive sympathetic activity exacerbates kidney injury by inducing renal vasoconstriction, reducing blood flow, and activating fibrotic pathways (Dibona and Kopp 1997). VNS provides renoprotection by restoring autonomic balance, inhibiting sympathetic hyperactivity, enhancing renal blood flow, and attenuating oxidative stress, thereby preserving renal function and structure against progressive damage.

Chronic kidney disease (CKD) is characterized by progressive loss of renal function accompanied by chronic inflammation, oxidative stress, autonomic imbalance, and fibrotic remodeling (Converse Jr et al. 1992; Xu et al. 2025). Similar to AKI, VNS may also exert anti‐inflammatory, antioxidative, and autonomic imbalance effects on CKD patients, while preclinical and clinical evidence is still emerging (Hilderman and Bruchfeld 2020). Future studies are needed to confirm VNS effects in CKD.

### 5.8 Other Disease

#### 5.8.1 Long COVID

Long COVID, known as “Post‐COVID‐19 Condition,” is characterized by the persistence or emergence of new symptoms at least 3 months following initial SARS‐CoV‐2 infection. This condition can affect individuals regardless of the severity of their initial illness and encompasses a wide range of symptoms, including fatigue, cognitive dysfunction, and respiratory issues. Many long COVID patients exhibit signs of dysautonomia (e.g., tachycardia and breathing irregularities) suggestive of excessive sympathetic drive and reduced vagal tone. VNS has shown effects on alleviating the symptoms of long COVID through multiple mechanisms (Pfoser‐Poschacher et al. 2025; Zheng et al. 2024). VNS may help restore autonomic balance by augmenting vagal efferent activity, normalizing sympathovagal interaction, stabilizing cardiovascular control, and modulating baroceptor sensitivity (Khan et al. 2024; Pfoser‐Poschacher et al. 2025; Soltani et al. 2023). This could help relieve postural orthostatic tachycardia syndrome (POTS), which is commonly reported in long COVID patients (Chakraborty et al. 2023). Another leading hypothesis for long COVID is that persistent immune activation and a cytokine milieu underlie ongoing symptoms such as fatigue and malaise. VNS could suppress proinflammatory cytokines, which may remain dysregulated in long COVID (Khan et al. 2024). In addition, chronic neuroinflammation could dysregulate brain networks, contributing to cognitive impairment (“brain fog”), mood disturbances, and profound fatigue. The neuroplasticity enhancement and synaptic remodeling effects of VNS may increase cerebral perfusion, which counteract the sluggish cortical activity and cognitive slowing seen in post‐COVID symptoms (Khan et al. 2024). Early report suggests that taVNS may reinvigorate underactive brainstem circuits in long COVID patients, helping to restore alertness and cognitive functions (Khan et al. 2024; Colzato et al. 2023).

## 6 Limitations and Future Directions

### 6.1 Limitations

Though VNS has shown therapeutic effects across multiple medical conditions, it faces several important limitations that span biological, technological, and methodological domains. Addressing these constraints is crucial for improving the safety, efficacy, and accessibility of VNS therapy.

#### 6.1.1 Biological Limitations

The VN's anatomy and fiber composition vary significantly between individuals, which contributes to variability in VNS outcomes. Studies of human cadaver VN have found that the number and arrangement of nerve fascicles differ widely across human beings (vagus fascicle count ranging from 5 to 10 in a normal individual) (Ottaviani et al. 2022). Similarly, the proportion of fiber types can differ by person (Yap et al. 2020). This anatomical heterogeneity means that a given set of stimulation parameters may activate different fiber subsets in different patients. One individual's VN may have fibers arranged such that therapy preferentially engages the intended targets, while another's anatomy could lead to more off‐target stimulation. Such differences likely underlie the high variability in clinical response to VNS.

Evidence also shows that the body can adapt to chronic VNS over time, leading to diminished therapeutic effects if not appropriately and regularly tuned for chronic use (Xu et al. 2022). In long‐term stimulation, neural plasticity may reduce the nerve's responsiveness, a phenomenon analogous to tolerance. Clinical experience in epilepsy, for example, suggests that while VNS can be effective, its benefits may plateau or wane in some patients after prolonged use, potentially due to physiological habituation (Koszewski et al. 2003). Therefore, Howland noted that stimulation often needs re‐adjustment over time as tolerance develops with continuous VNS (Howland 2014). Moreover, the nervous system may undergo homeostatic changes (e.g., receptor downregulation or synaptic remodeling) in response to ongoing stimulation, which could reduce VNS efficacy. This intrinsic plasticity of biological systems is recognized as a limitation to sustained open‐loop stimulation. In other words, the vagal circuits learn to compensate for the constant stimulus (Ottaviani et al. 2022). Therefore, the potential for adaptation means that the initial benefits of VNS might not always persist undiminished long‐term without parameter changes.

#### 6.1.2 Technical Limitations

A major limitation of current VNS technology is its inability to selectively stimulate functionally distinct fibers within the VN: it activates a mix of vagal fibers (both afferent and efferent) based largely on fiber size and threshold, rather than targeting specific pathways. For instance, large, myelinated A‐fibers are recruited at a lower threshold, while smaller unmyelinated C‐fibers, often implicated in anti‐inflammatory and nociceptive pathways, require higher stimulation intensities that may exceed tolerability limits. Clinically, this lack of selectivity manifests as off‐target side effects such as voice changes, coughing, or throat discomfort due to unintended activation of nearby somatic branches (e.g., recurrent laryngeal motor fibers) (Fitchett et al. 2021). It also complicates therapeutic interpretation, as the same stimulation may engage excitatory and inhibitory pathways simultaneously or trigger opposing physiological responses. On the other hand, such non‐selective activation not only produces unwanted symptoms but may also limit efficacy, since reducing stimulation intensity to avoid side effects can fail to recruit smaller, high‐threshold C‐fibers that might carry beneficial signals and be crucial in a coordinated CAP (Fitchett et al. 2021). Thus, biological fiber organization imposes an inherent challenge: different functional circuits are intermingled, and their selective access requires high spatial and physiological resolution.

In addition, though iVNS has proven to have the most robust and well‐established benefits in certain disorders, it requires the implantation of the pulse generator and lead, which introduces surgical risk and potential hardware failures. The surgery carries risks such as infection, hematoma, or nerve injury (Révész et al. 2016). Even after successful implantation, the hardware can present complications over time. Studies with long‐term VNS for epilepsy report that lead breakage or lead malfunction occurs in roughly 3% of patients, often requiring surgical lead replacement. Battery depletion is inevitable as well; battery life spans 3–8 years, depending on usage, after which replacement surgery is needed (Révész et al. 2016). Each additional surgery carries a cumulative risk of complications such as infection or scarring around the nerve. There are also rare device malfunctions (e.g., surgical cable break, unexpected stimulator events) reported in a small fraction of patients (Révész et al. 2016). Therefore, the need to balance durable, effective stimulation with these hardware issues is a clear limitation of current VNS therapy.

Another technical limitation of the current VNS technique is the lack of closed‐loop control. Present VNS systems are commonly operated in an open‐loop manner, delivering continuous stimulation at predetermined settings, without real‐time adjustment to the patient's physiological state. In an open‐loop device, parameters (frequency, pulse width, duty cycle, etc.) are fixed and cannot automatically respond to changes such as impending seizure, fluctuations in inflammatory markers, or other biometrics. Consequently, the patient may receive stimulation when it's not needed, and conversely, the device may not ramp up stimulation during critical moments where it would be beneficial. The fixed‐output nature of VNS means it cannot dynamically optimize its stimulation in response to patient needs, which is a clear limitation, especially as VNS is expanded to new indications where timing and dosage of stimulation might critically matter (Ottaviani et al. 2022).

#### 6.1.3 Methodological Limitations

##### 6.1.3.1 Heterogeneity in Clinical Trial Design and Reports

VNS clinical trials have exhibited substantial heterogeneity in their design and reporting, which limits the comparability and generalizability of findings across different indications. First of all, trials have enrolled diverse cohorts—for example, some studies mix pediatric and adult patients or include various etiologies and comorbidities, leading to inconsistent characteristics (Veldman et al. 2025). Even within a single indication like depression, inclusion criteria (e.g., definition of treatment‐resistant depression) often differ, contributing to outcome disparities (McIntyre et al. 2023). Second, VNS studies range from small pilot trials with only a few patients to larger multi‐center studies with thousands. Many early or exploratory trials had limited sample sizes and short follow‐up periods, yielding only preliminary insights. In contrast, some studies extend over years. These differences in scale and duration affect statistical power and the ability to observe long‐term effects (Austelle et al. 2022). Third, there is a lack of uniformity in the use of control groups and blinding. Some trials are double‐blind, sham‐controlled, whereas others are open‐label or uncontrolled case series. Notably, in a depression meta‐view report, the only large RCT reported no significant difference between active VNS and sham treatment, while many uncontrolled studies showed improvements, highlighting how trial design can yield conflicting efficacy results (Martin and Martin‐Sanchez 2012). On the other hand, inadequate blinding or concurrent changes in standard therapies during open‐label VNS can introduce placebo and confounding effects, making it difficult to attribute outcomes solely to VNS (Veldman et al. 2025). Lastly, studies define and measure outcomes differently. In epilepsy trials, primary endpoints might be reduction in seizure frequency or proportion of responders (Lim et al. 2022; Abbasi et al. 2021; Melese et al. 2024; Kong et al. 2024), whereas in depression trials, outcomes include various rating scales such as Hamilton Rating Scale for Depression (HAM‐D) and MADRS or remission rates (Bottomley et al. 2020). Other investigations use biomarkers or quality‐of‐life endpoints. The lack of standardized outcome metrics hampers direct comparison: a positive outcome in one trial might not be defined or assessed in another. For instance, across VNS studies in GI disorders, outcome heterogeneity (different symptom scales and endpoints for each condition) was so pronounced that meta‐analysis could not be performed (Veldman et al. 2025).

The need for standardized trial design and reporting in VNS research is increasingly recognized. For example, the 2025 International Headache Society evidence‐based guidelines for noninvasive neuromodulation devices highlighted significant methodological concerns across existing RCTs, including imprecision due to small sample sizes, inadequate sham control, and parameter variability across studies; the guidelines emphasized that future trials should adhere to consensus standards for neuromodulation device trials (Yuan et al. 2025). The guidelines also highlighted that future trials should adhere to the International Headache Society's consensus standards for neuromodulation device trials. This heterogeneity in patient populations, study design, outcome measures, and stimulation protocols limits meaningful cross‐study comparison and robust meta‐analytic conclusions.

##### 6.1.3.2 Variability in Stimulation Parameters

There is a wide variability in the stimulation protocols used for VNS, reflecting a lack of standardized parameters across both invasive and noninvasive modalities. There is no “standard” VNS dosing protocol. As a result, studies employ a broad range of stimulation parameters, including differences in pulse widths (range from 100–200 μs to 500 μs–500 ms), frequency (range from 5 to 50 Hz; some studies use kHz range VNS for vagal block), amplitude (iVNS range 0.2–6 mA, whereas noninvasive VNS require higher current) and duty cycle (uniform and non‐uniform, short burst or long pause) (Table 1) (Thompson et al. 2021). In addition, the duration of therapy varies significantly across studies. The length of time patients receive VNS in trials ranges from acute single‐session experiments to chronic stimulation over months or years. Short‐term studies may capture immediate neuromodulatory effects but miss long‐term adaptations, whereas prolonged trials incur changes in concurrent treatment and disease course (Table 1). Inconsistent therapy duration makes it hard to determine the timeframe needed for VNS to achieve maximal benefit and complicates comparisons of efficacy across studies.

This considerable variability in stimulation parameters makes it difficult to reproduce and compare results between studies (Olsen et al. 2023; Thompson et al. 2021). Importantly, optimal parameters for VNS, particularly noninvasive VNS, remain undetermined (Yap et al. 2020; Thompson et al. 2021; Badran et al. 2018). While stimulation parameters have a significant impact on therapeutic efficacy, the influence of parameters such as frequency, pulse width, intensity, and duty cycle on clinical outcomes remains incompletely understood. Without a standardized protocol, it is challenging to identify which parameters are truly optimal, as each study's positive or negative results might partly be due to the chosen settings rather than the intrinsic capability of VNS.

### 6.2 Emerging Technologies and Future Directions

#### 6.2.1 Selective VNS

Early efforts to improve VNS specificity have focused on innovative electrode designs and stimulation paradigms that confer spatial selectivity along the nerve. Instead of a standard two‐electrode cuff that stimulates the entire nerve, spatial‐selective VNS uses multi‐contact electrode arrays to direct current to a restricted portion of the nerve's cross‐section (Ravagli et al. 2023). By empowering electrical stimulation in this manner, specific functional fiber groups can be excited while others are relatively spared. Stimulation and in vivo studies in large animals have shown that such multi‐contact cuffs can indeed achieve organ‐specific stimulation. For example, an optimized four‐contact cuff in sheep could drop respiratory rate by 90% without inducing bradycardia or conversely reduce HR by 27% with minimal impact on breathing. To further refine this approach, Fast Neural Electrical Impedance Tomography (FN‐EIT) has emerged as a powerful adjunct tool. FN‐EIT enables real‐time imaging of neural activity across the nerve cross‐section, mapping the spatial origin of action potential, and thereby guiding electrode selection for targeted stimulation (Fitchett et al. 2021; Ravagli et al. 2023).

Another electrical strategy to enhance selectivity is the use of anodal block and directional stimulation techniques. Anodal block involves delivering hyperpolarizing current to a segment of the nerve to locally prevent action potential propagation, thus allowing stimulation in one direction or of one fiber type while blocking others. Research in rats demonstrated that changing the orientation of the stimulus can bias activation toward afferent versus efferent fibers: anode distal stimulation preferentially evoked afferent responses (e.g., changes in breathing rate), whereas reversing the polarity produced predominantly efferent effects (e.g., HR changes) (Ahmed et al. 2020). This directional bias is consistent with anodal blockade of fibers, allowing selective excitation of fibers projecting in the opposite direction.

In parallel with electrode innovations, researchers are exploring novel stimulation modalities that could inherently target specific vagal pathways by their biological or physical mechanism. Specifically, the magnetic stimulation and optogenetic stimulation introduced in the VNS approach section show promising results in increasing selectivity (Jeong et al. 2022; Booth et al. 2021; Fontaine et al. 2021). While far from clinical use, they represent an inventive frontier for achieving selective vagus activation through biophysical means.

Preclinical work in mice by Lim et al. and rats by Puleo et al. recently demonstrated that FUS spleen stimulation effectively reduced inflammatory arthritis and LPS‐induced inflammatory cytokine release, respectively (Zachs et al. 2019; Cotero et al. 2019). The work has now transitioned to humans; FUS spleen stimulation reduced LPS‐spiked blood cytokine production in participants treated with FUS spleen stimulation (Graham et al. 2020; Zanos et al. 2023). These findings underpin the potential of FUS as a transformative tool in immunomodulation therapies that achieve selective vagal efferent and/or afferent circuit activation.

FUS has also been applied to other non‐splenic targets, such as the liver, gastrointestinal tract, and various peripheral ganglia (Cotero et al. 2022, 2020; Ashe et al. 2023). Evidence suggests that FUS modulates nerve pathways within those other targets through a subset of mechanically sensitive ion channels, which are known to have cell, tissue, and organ‐specific expression profiles. The ability to image‐target FUS pulses (Ashe et al. 2023) and design therapies that target specific nerve innervation fields has already been shown to provide unique outcomes compared to traditional electrical VNS (Cotero et al. 2019, 2022, 2020; Ashe et al. 2023), and further study is now warranted of the potential FUS parameter (i.e., ultrasound pulse amplitude, length, frequency, and duration) specific interactions with the different mechanically sensitive ion channel types to reveal the benefits and limitations of nerve fiber and cell specificity of FUS neuromodulation (Duque et al. 2022).

#### 6.2.2 Closed‐Loop VNS

Closed‐loop VNS systems adaptively modulate stimulation in real time based on physiological, neurological, molecular, or behavioral feedback signals. Instead of delivering fixed, pre‐programmed pulses, these systems use biomarkers of the patient's state to trigger or adjust vagal stimulation on demand, potentially providing personalized treatment that accounts for individual variability. Key biomarkers explored for guiding closed‐loop VNS include heart‐derived signals, neural activity patterns, immune markers, respiratory metrics, and measures of patient motion or state.

**FIGURE 7:**
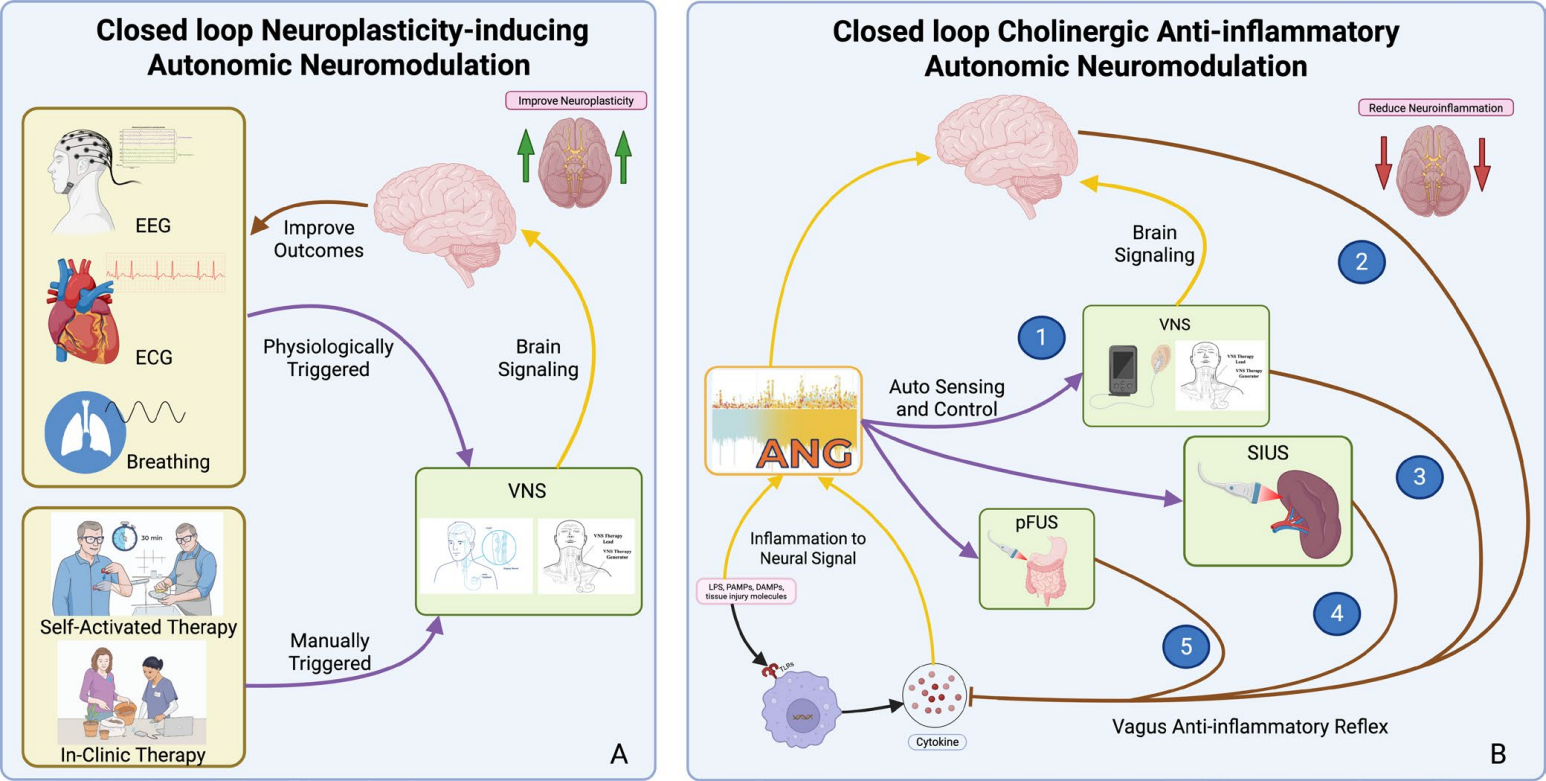
Schematic representation of closed‐loop autonomic neuromodulation paradigms. By definition, closed‐loop neuromodulation requires a continuous sensing modality that continuously modifies the stimulation parameter (yellow arrows = afferent sensing pathway; purple arrows = VNS device control, brown arrows = efferent regulation pathway). (A) Closed‐loop neuroplasticity‐inducing autonomic neuromodulation. VNS can be delivered through two triggering approaches: (1) physiologically triggered stimulation using real‐time biosignals including electroencephalograph (EEG), electrocardiography (ECG), and respiration patterns to initiate VNS based on detected physiological states automatically; or (2) manually triggered stimulation through patient self‐activated therapy or clinician‐administered in‐clinic training session. Both approaches aim to enhance neuroplasticity outcomes through precise timed vagal neuromodulation. (B) Closed‐loop cholinergic anti‐inflammatory autonomic neuromodulation. Future closed‐loop autonomic neuromodulation is under development, in which inflammation is sensed with Autonomic Neurography (ANG) that can then drive autonomic neuromodulation delivery or hone in on the dosage aimed to amplify the body's own vagus‐driven anti‐inflammatory reflex. The system may operate through multiple interconnected components: (1) ANG provides continuous monitoring and auto‐sensing for the neural signature associated with inflammatory states; this sensing then controls VNS delivery to directly modulate brain neuroinflammation; (2) brain signaling modulation in turn regulates descending vagus anti‐inflammatory reflex; (3) VNS exerts direct anti‐inflammatory effects through vagal efferent activation; (4) Splenic Immune Cell Ultrasound (SIUS) modulation activates the cholinergic anti‐inflammatory pathway via splenic neuroimmune modulation; (5) Peripheral focused ultrasound (pFUS) targets the gastrointestinal system to modulate the brain‐gut axis, engaging the vagal anti‐inflammatory reflex. This auto‐sensing and control architecture enables personalized, responsive immunomodulation by dynamically adjusting stimulation parameters based on real‐time inflammatory biomarkers and neural activity patterns.

The transition from open‐loop to closed‐loop VNS has been driven by the recognition that therapeutic efficacy can be substantially enhanced when stimulation is precisely timed to physiological events, utilizing real‐time inputs such as ECG, EEG, or respiration monitoring to trigger or modulate vagal activation at optimal moments (Figure 7A).

HR is a fundamental autonomic indicator and common trigger for closed‐loop VNS. A sudden spike in HR often heralds seizures or acute stress, providing a prompt for therapeutic stimulation. FDA‐approved closed‐loop VNS devices for epilepsy already leverage this: the system continuously detects the patient's heartbeat and delivers vagal stimulation automatically upon a rapid HR increase associated with seizure (Afra et al. 2021). The *AspireSR* “sense and respond” generator, introduced in 2015, was first to feature this automatic stimulation mode, followed by the *SenTiva* generator in 2017. This innovation enhances responsiveness and precision compared to traditional open‐loop systems, providing more effective therapeutic intervention during clinical moments such as tachycardia‐associated seizure (Winston et al. 2021). In addition, continuously tracking HRV allows a system to gauge if the body is under stress or inflammation, and a significant change in HRV can signal the need for adjusting vagal stimulation to rebalance autonomic tone. The rationale is that by boosting vagal output when HRV deteriorates, the system can promote a shift toward parasympathetic activity, which is linked to anti‐inflammatory effects and can help calm the physiological state (Lerman et al. 2025). Additionally, novel techniques are being developed to target the stimulation during specific parts of the cardiac cycle, such as systole‐gated and diastole‐gated (Tischer et al. 2025). A pilot study found that systole‐gated taVNS tends to prolong RR intervals when stimulated after the R peak and shorten them before, whereas for diastole‐gated taVNS, later stimulation onset within diastole tends to result in longer subsequent RR intervals. The systole‐gated taVNS showed the largest cardiovagal modulatory capacity, indicating a more effective method to influence parasympathetic activity. It is theorized that synchronizing vagal stimulation with this period of heightened receptivity enhances its therapeutic impact (Tischer et al. 2025).

EEG monitoring of brain activity is another valuable input for closed‐loop VNS in neurological disorders, especially epilepsy. Seizures are preceded by characteristic EEG patterns that can be detected in real time. By embedding EEG electrodes, in‐development closed‐loop VNS can identify an impending seizure and trigger stimulation at or before ictal onset (Shoeb et al. 2007). By intervening at the electrical buildup stage of a seizure, the closed‐loop VNS potentially prevents full manifestation, thereby improving patients' outcomes. This approach aligns with advancement in closed‐loop taVNS, which uses a real‐time biofeedback mechanism such as EEG, ECG, and humoral signals (Yu, Ling, et al. 2022).

In addition, breathing parameters (rates, depth, rhythm) are tightly coupled to autonomic outflow, and closed‐loop VNS can leverage these signals in a couple of ways. First, respiration can serve as a timing signal: vagal activity synchronizes with RSA. A VNS system can therefore synchronize stimulation with a particular phase of breathing. For example, delivering pulses during exhalation to enhance the targeting effect of VNS on the NTS (Han, Zhang, et al. 2024) as described by Napadow et al. (2012). Such technology is also known as respiratory‐gated VNS, emerging as a novel technique for closed‐loop VNS; however, respiration does not necessarily change with current parameter sets by this group. Second, abnormal respiratory patterns can act as triggers: apneas, hyperventilation, or irregular breathing could prompt VNS if the goal is to stabilize cardiorespiratory function. An example application is in neuropsychiatric disorders, where accelerated breathing indicates rising anxiety; a closed‐loop VNS could detect and stimulate to induce calm (Nayok et al. 2023).

Manually triggered closed‐loop VNS represents a distinct paradigm wherein stimulation is precisely synchronized with rehabilitation movement through real‐time patient or therapist activation (Figure 7A). The *Vivistim* iVNS system (MicroTransponder Inc.) exemplifies this approach, receiving FDA approval in 2021 as the first neuromodulation device indicated for chronic ischemic stroke upper limb rehabilitation. The system uniquely integrates two delivery modes: during in‐clinic sessions, the therapist triggers stimulation pulses via a wireless button at the moment of successful movement; for home‐based therapy, the patient self‐activates exercise sessions by swiping a handheld magnet over the implanted pulse generator. The paired VNS concept exploits the synaptic eligibility trace, a 5–10 s window during which neuromodulator release can convert activated synapses into lasting plasticity changes, while a delayed 15–25 s tone failed to generate plasticity (Engineer et al. 2019). The pivotal VNS‐REHAB RCT demonstrates that VNS nearly doubled the clinical response rate compared to the sham group (47% vs. 24%, *p* < 0.01) after 90 days of therapy (Dawson et al. 2021) and progressive gains reaching 85.7% responders at 3‐year follow‐up with continued home‐based therapy (Francisco et al. 2023).

Novel technologies also show direct, noninvasive measurement of vagal and other autonomic nerve activity through the electric field by surface electrode arrays and the magnetic field by optically pumped magnetometers (Bu, Kurniawan, et al. 2022; Bu, Prince, et al. 2022; Bu et al. 2024). Our group has termed this approach as Autonomic Neurography or ANG. The ANG neural firing activity measured showed a direct correlation to: (1) inflammatory cytokine concentrations elicited by intravenous LPS injection in human participants, and (2) autonomic stress challenges such as a deep breathing challenge and the cold pressor test; in aggregate, direct neural decoding through noninvasive measurements has now been demonstrated in humans (Bu, Kurniawan, et al. 2022; Bu et al. 2024). It is a powerful paradigm shift; VN and other autonomic neuronal (sympathetic chain) signaling itself could be used as a sensor to drive and titrate the neural stimulation response, and it may now pave the way for truly closed‐loop neural immune circuit engineering. The vision is that the closed‐loop system continuously monitors neural firing activity from the VN and activates VNS when inflammatory activity exceeds a threshold, thereby precisely modulating the immune system in real time (Lerman et al. 2025) (Figure 7B). Our groups are currently testing this paradigm by monitoring human ANG prior to LPS intravenous injection whereby we calibrate the amount of FUS spleen stimulation, aiming to temper the inflammatory response optimally. The work highlights the need for immediacy in closed‐loop neuromodulation. For instance, if immediate alteration in afferent signaling (inflammatory or other) indicative of disease progression is identified, instantaneous VNS may be deployed, thereby allowing clinicians to leverage the improved effects of early/instantaneous VNS on disease states (Lerman et al. 2025). Certain use cases may require these autonomous systems; namely, provision of care in austere or under‐resourced environments that may preclude high levels of care, that is, Intensive Care Unit (ICU) level care for the expected prolonged periods of time during medical evacuation.

Additionally, evidenced from peripheral FUS on celiac plexus to ameliorate symptoms in IBD (Akhtar et al. 2021), subclinical gut to brain vagus nerve modulation techniques are being investigated by our groups; closed‐loop systems in which ANG coupled to molecular‐based sensors identify subclinical and or clinical dysbiosis (transmitted from gut to brain) are prime targets in which a user may be notified to engage in neuromodulation and or provided individualized pre‐, pro‐biotic, and or RNA‐Instructed Sequence Replacement (RISR) pathobiont replacement therapies.

#### 6.2.3 Artificial Intelligence‐Guided and Personalized Neuromodulation

Artificial intelligence (AI) is increasingly being leveraged to enhance VNS therapy through personalized, adaptive strategies. Emerging AI‐guided approaches use patient‐specific data and real‐time feedback to tailor VNS parameters for optimal outcomes. One major application of AI in VNS is the optimization of stimulation parameters on an individual basis. Advanced algorithms are capable of navigating complex parameter spaces to identify specific settings that maximize therapeutic benefits. For example, a study utilized reinforcement learning to capture the dynamic relationship between varying VNS parameters and resulting neural and physiological responses in a rat cardiac model, aiming to optimize VNS settings that maintain target set points (Sarikhani et al. 2024). This data‐driven approach can inform personalized “dosing” of VNS. In practice, AI algorithms can even adapt stimulation on the fly: one recent study proposed that AI could adjust VNS intensity in real time based on a patient's pain level (Nandakumar et al. 2024). Similarly, computational modeling combined with AI‐based optimization has been shown to accelerate the design of selective VNS paradigms, helping target specific nerve fibers or functions while minimizing trial‐and‐error in experiments (Secerovic et al. 2025).

AI is also being applied to predict patient‐specific responses to VNS and guide personalized treatment decisions. Machine learning models can analyze pre‐treatment biomarkers and neurological data to forecast which patients are likely to benefit from VNS. For example, researchers have trained classifiers on HRV measures to distinguish likely responders from non‐responders in drug‐resistant epilepsy (Fang et al. 2021). Similarly, Chen et al. identified brain connectivity patterns from scalp EEG and built a machine learning classifier that could prospectively discriminate pediatric epilepsy patients who would respond to VNS therapy (Chen, Wang, et al. 2023). Such predictive analytics enable a more personalized neuromodulation strategy, potentially allowing clinicians to customize VNS parameters or select candidates most likely to respond, thereby improving overall treatment efficiency.

#### 6.2.4 Standardized Protocol, Device Programming, and Clinical Integration

VNS parameters and protocols have historically varied widely across studies and clinical practices, with little consensus on optimal settings (Thompson et al. 2021). Recognizing this inconsistency, recent efforts aim to establish more uniform frameworks for VNS protocol design. For example, an international multi‐disciplinary consensus in 2020 issued reporting standards for transcutaneous VNS research, recommending that future studies uniformly document stimulation parameters, patient criteria, and outcomes (Farmer et al. 2021). Such guidelines encourage harmonization across VNS studies, making findings more comparable.

Next‐generation VNS devices are incorporating more sophisticated programming capabilities to facilitate personalized stimulation. Modern implantable generators (FDA‐approved) now support programmable features like scheduled ramp‐ups and multi‐period cycles. For instance, the latest models allow automated titration of output current over time and distinct day/night stimulation profiles to align with circadian patterns to treat drug‐resistant epilepsy (Afra et al. 2021). Furthermore, device interoperability with the healthcare system is improving. VNS programming platforms now interface with telemedicine and hospital IT infrastructure: a recent proof‐of‐concept demonstrated fully remote VNS programming via secure telehealth, with clinicians adjusting device settings from off‐site (Rossi and Gonzalez‐Martinez 2020). Such capabilities hint at a future where routine VNS adjustments and monitoring can be standardized across distances.

In parallel with technical innovations, the VNS field is investing in clinical integration efforts to ensure the therapy is delivered in a consistent and patient‐centered manner. Multicenter collaborations and data‐sharing consortia are playing a key role in this harmonization. For example, the CORE‐VNS registry has enrolled hundreds of patients across dozens of centers worldwide to systematically collect outcomes and programming data in drug‐resistant epilepsy. By aggregating real‐world data at scale, such registries enable clinicians to identify best practices and converge on effective parameter ranges (Sen et al. 2021). Another critical aspect is clinical training and education. To support uniform adoption of VNS protocols, professional societies have developed a dedicated curriculum for implanting and managing VNS devices (Bieber et al. 2025). This ensures that surgeons are updated on the latest protocol, device features, and safety considerations, reducing inter‐operator variability. In the longer term, the identification of reliable biomarkers and integration of wearable sensors may enable continuous monitoring of patient state to dynamically inform VNS therapy (Austelle et al. 2024; Lerman et al. 2025). In sum, the convergence of consensus protocols, advanced programming devices, and collaborative clinical infrastructure is paving the way for more standardized yet personalized VNS therapy in the future.

#### 6.2.5 Over‐the‐Counter VNS for Wellness

Beyond FDA‐approved/cleared medical devices for specific disease conditions and patient populations, an over‐the‐counter (OTC) VNS device market has emerged for general wellness applications. These consumer‐grade devices commonly employ noninvasive taVNS and tcVNS techniques. For example, *Parasym* has taVNS devices with controllers that are sold in Europe (named as *Nurosym*) and the U.S. Market (named as *NuroPod*). Similar taVNS devices include *Xen*, *ZenoWell*, *tVNS*, and *Dolphin Neurostim*. For tcVNS, *Truvaga* is a handheld device built by electroCore Inc., the same company behind *GammaCore*. *Pulsetto* is a hands‐free, neck‐worn tcVNS device with an App for mode control. Hoolset provides both a handheld tcVNS solution (*Verelief*) and a hands‐free headphone bilateral tcVNS (*Hoolset*) technology. Additionally, *Sensate*, placed on the chest, uses infrasonic chest vibration that claims to tone the VN while specifics are lacking on modality engaged. These wellness devices are marketed for a panapoly of therapeutic purposes, including stress and anxiety management, sleep enhancement, GI motility improvement, cognitive focus optimization, HRV regulation, and hormone rebalance.

However, clinical evidence varies dramatically and is uniformly lacking RCT across devices. For example, *tVNS* (formerly *Nemos*) is the most widely studied taVNS device. It has obtained the CE mark and is available in Europe, yet not in North America. It has been used in studies for conditions such as tinnitus (Kreuzer et al. 2014), epilepsy (Bauer et al. 2016), chronic migraine (Straube et al. 2015), stroke rehabilitation (Redgrave, Moore, et al. 2018), and more. Similarly, *Parasym* devices have been studied in a pilot RCT to treat HFpEF and demonstrated apparent inflammation regulation (Stavrakis et al. 2022). Other studies have explored the device's effects on postural tachycardia syndrome (Stavrakis et al. 2024), chronic HF (Gentile et al. 2025), and long COVID symptoms (Zheng et al. 2024). Additionally, *Dolphin Neurostim* is FDA‐cleared as an OTC transcutaneous electrical nerve stimulation device for relieving pain associated with sore and aching muscles; recently it has also been explored as a taVNS therapy device (Armstrong et al. 2017). *Truvaga* leverages electroCore's extensive *GammaCore* research portfolio, but the consumer wellness device itself lacks independent clinical trials. Despite the growing popularity of consumer VNS devices for wellness, a critical evidence gap exists between marketed claims and peer‐reviewed validation. While devices such as *Parasym* have accumulated some RCT evidence, most OTC devices lack peer‐reviewed clinical validation. Furthermore, stimulation parameters vary substantially across devices, with some (e.g., *Pulsetto* at 4500–5200 Hz) operating far outside the 10–30 Hz frequency range that is commonly studied in taVNS research, raising questions about the mechanism and its equivalence. Therefore, consumers and clinicians should recognize that the regulatory classification as “general wellness devices” exempts these products from demonstrating clinical efficacy.

## 7 Conclusion

VNS represents a powerful and increasingly versatile neuromodulatory strategy capable of influencing a wide range of physiological systems through both central and peripheral mechanisms. The converging evidence across preclinical and clinical studies confirms that VNS exerts its therapeutic effects through modulation of the neurochemical system, facilitation of neuroplasticity, regulation of autonomic and endocrine axes, and engagement of anti‐inflammatory and immunomodulatory pathways. This systemic effect has enabled VNS to treat a diverse spectrum of disorders, including neurological, psychiatric, cardiovascular, immunologic, metabolic, and gastrointestinal conditions. However, challenges remain in standardizing stimulation parameters, improving patient‐specific targeting, and addressing biological variability in treatment response. Advances in closed‐loop systems, multi‐modal continuous sensor suite development, selective fiber targeting, AI‐driven personalization, and standardized protocol promise to refine VNS into a more precise and adaptive therapy. Continued multidisciplinary research is essential to refine VNS technologies and expand their clinical applicability, ensuring that future applications are grounded in mechanistic insight and optimized for therapeutic specificity.

## Funding

This work was supported by BARDA Contract (75A50119C00038), DARPA Contract (W911NF2010027), Katz Neural Sensor Development Fund, and Ginsburg Autonomic Endotyping Fund. Salary support for co‐authors was provided by the Department of Veterans Affairs (R.K.: IK2RX004777) and by British Heart Foundation (O.C.: SI/F/24/21170016).

## Disclosure

All the content in this manuscript was written by the authors. No generative AI or AI‐assisted technology was used.

## Conflicts of Interest

Yifeng Bu and Imanuel Lerman are affiliated with InflammaSense. Hubert Lim serves as Chief Science Officer of the SecondWave System. The remaining authors declare no conflicts of interest.

## Data Availability

Data sharing is not applicable to this article as no datasets were generated or analyzed during the current study.
